# Accelerated, high-quality photolithographic synthesis of RNA microarrays in situ

**DOI:** 10.1126/sciadv.ado6762

**Published:** 2024-07-31

**Authors:** Tadija Kekić, Nemanja Milisavljević, Joris Troussier, Amina Tahir, Françoise Debart, Jory Lietard

**Affiliations:** ^1^ Institute of Inorganic Chemistry, University of Vienna, Josef-Holaubek-Platz 2, 1090 Vienna, Austria.; ^2^ IBMM, University of Montpellier, CNRS, ENSCM, Montpellier, France.

## Abstract

Nucleic acid photolithography is the only microarray fabrication process that has demonstrated chemical versatility accommodating any type of nucleic acid. The current approach to RNA microarray synthesis requires long coupling and photolysis times and suffers from unavoidable degradation postsynthesis. In this study, we developed a series of RNA phosphoramidites with improved chemical and photochemical protection of the 2′- and 5′-OH functions. In so doing, we reduced the coupling time by more than half and the photolysis time by a factor of 4. Sequence libraries that would otherwise take over 6 hours to synthesize can now be prepared in half the time. Degradation is substantially lowered, and concomitantly, hybridization signals can reach over seven times those of the previous state of the art. Under those conditions, high-density RNA microarrays and RNA libraries can now be synthesized at greatly accelerated rates. We also synthesized fluorogenic RNA Mango aptamers on microarrays and investigated the effect of sequence mutations on their fluorogenic properties.

## Teaser

An improved set of RNA building blocks allows for better, faster fabrication of high-density RNA microarrays.

## INTRODUCTION

The structural and functional landscape of RNA is a realm of constant exploration, and its relevance in medicine and biotechnology has recently soared with the advent and market approval of RNA-based therapeutics (*1*, *2*). There is consequently an ever-growing need for synthetic RNA to probe its properties, and methodologies that can also accommodate chemical modifications are particularly essential (*3*). Likewise, throughput-driven approaches are highly sought after because they can deliver nucleic acid libraries of any arbitrary sequence and offer the possibility of running assays in parallel. Large sequence libraries can be assembled into longer, more complex products such as genes or nanostructures (*4*, *5*), while multiplexing assays can quickly return useful information on genome and gene expression (*6*–*8*), protein binding (*9*–*12*), or sequence dependence (*13*, *14*). High-throughput nucleic acid synthesis has been commonplace for DNA because the development of microarrays, particularly those where DNA synthesis takes place in situ on the array surface, yielding up to hundreds of thousands of unique sequences in a single run (*15*). The chemistry of DNA microarray synthesis draws heavily on classical solid-phase synthesis using phosphoramidite building blocks (*16*–*19*), although very recent advances have demonstrated the possibility of adapting de novo enzymatic synthesis to microarray fabrication (*20*, *21*). RNA microarray synthesis has received far less attention due to the technical challenges associated with RNA instability, synthesis efficiency, and the protection group strategy for the 2′-OH function. The latter is the primary reason for the delay in adapting array fabrication techniques to RNA because the 2′-*O*-silyl protection route conventionally used in solid-phase oligonucleotide synthesis is incompatible with silicon and glass surfaces. Solutions to this conundrum aimed to forego the use of phosphoramidites and, instead, to convert DNA microarrays into RNA with suitable polymerases (*22*–*28*). While these in situ transcription approaches require no specialized equipment and are accessible with off-the-shelf consumables, they generally suffer from moderate yields and are limited to canonical DNA and RNA. Phosphoramidite chemistry is currently the only practical gateway to chemical diversity in RNA. To set the stage for RNA microarray synthesis, we turned away from silyl-based protection of the 2′-OH group and instead opted for an acetal ester strategy. We chose the acetal levulinyl ester (ALE) group as acetals alleviate the risk of protecting group migration between 2′- and 3′-OH, and levulinyl moieties can be selectively removed by hydrazine treatment (*29*), making it compatible with microarray surfaces. We developed the corresponding set of RNA phosphoramidites equipped with a photosensitive nitrophenylpropyloxycarbonyl (NPPOC) group (*30*). Photosensitive phosphoramidites are the building blocks needed for in situ microarray synthesis by photolithography, where ultraviolet (UV) light can be directed to key positions on the array surface and selectively remove terminal NPPOC groups on the exposed areas (*31*). With 2′-*O*-ALE phosphoramidites, we extended the domain of maskless array synthesis (MAS) to RNA and assembled >260,000 unique sequences on the same surface (*32*). The technology has the added benefit of enabling the simultaneous synthesis of DNA and RNA, leading to the preparation of chimeric nucleic acid microarrays (*33*).

**Fig. 1.**
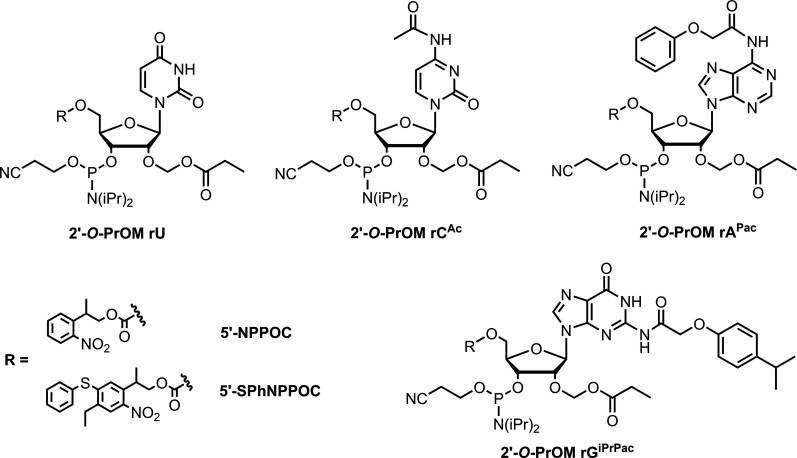
Chemical structures of the 2′-*O*-PrOM RNA phosphoramidites in the 5′-NPPOC and 5′-SPhNPPOC series developed for this study.

Despite synthetic ease and orthogonality, RNA microarray synthesis with ALE monomers suffers from important limitations. At the nucleoside level, the regioselectivity of NPPOC installation is poor, producing mixtures of 5′- and 3′-NPPOC–protected intermediates that are difficult to separate. In photolithography, the relatively long coupling time of ALE monomers—4 min on average, much longer than that of DNA (15 s)—and the incompressible photolysis time of the NPPOC group requiring a full minute of exposure to 365-nm UV light at 100 mW/cm^2^ are major contributors to the total array fabrication time. High-density RNA libraries require over 6 hours of synthesis, which limits not only throughput but also array quality, as repeated exposure to solvents and reagents affects surface integrity (*34*). In addition, we observed substantial RNA degradation, particularly during hydrazine treatment. These drawbacks prompted us to consider an alternative series of RNA monomers that would simultaneously address the speed, throughput, and quality in RNA photolithography. Acetal esters have been developed as 2′-OH protection for conventional RNA solid-phase synthesis, notably with pivaloyloxymethyl and propionyloxymethyl moieties (2′-*O*-PivOM and 2′-*O*-PrOM, respectively). In solid-phase synthesis, the corresponding PivOM and PrOM phosphoramidites couple faster than the ALE variants (3 versus 10 min, respectively) and with high efficiency (*35*–*39*). Given that the coupling kinetics are more favorable on flat reactive surfaces (*31*), we surmised that PrOM, the smallest of the 2′-acetal esters tested thus far, should fare better than ALE and unlock the 1-min coupling time mark for RNA microarrays. In keeping with our intent to lower the fabrication time and increase the throughput, we also considered installing a thiophenyl derivative of NPPOC at the 5′-OH position (SPhNPPOC), an improved photosensitive protecting group that reaches quantitative photolysis 12× faster than NPPOC in DNA photolithography (*40*). Here, we report on the preparation of photoprotected PrOM RNA phosphoramidites (Fig. 1) on a gram scale and describe how the synthesis of high-density RNA microarrays can be greatly accelerated, producing higher-quality RNA along with stronger hybridization signals. With this set of RNA building blocks, complex sequences libraries requiring ~200 coupling cycles are within reach and at a fraction of the synthesis time with legacy RNA monomers.

## RESULTS

### Phosphoramidite synthesis

**Fig. 2.**
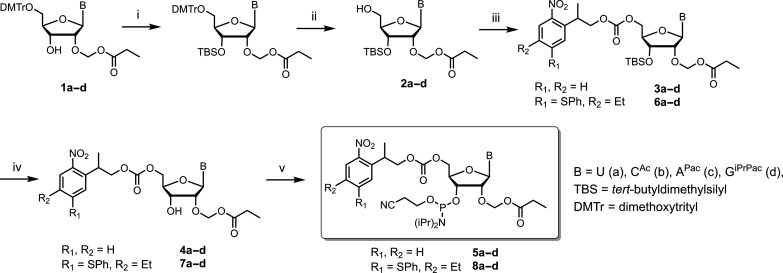
Synthesis route toward the preparation of all 2′-*O*-PrOM RNA phosphoramidites with 5′-NPPOC (R_1_, R_2_ = H) and 5′-SPhNPPOC (R_1_ = SPh, R_2_ = Et) photosensitive protecting groups. Reagents and conditions: (i) TBS-Cl, *N*,*N*′-dimethylformamide (DMF), r.t.; (ii) benzenesulfonic acid, CH_2_Cl_2_/MeOH, r.t.; (iii) NPPOC-Cl (**3**) or SPhNPPOC-Cl (**4**), 1-methylimidazole, CH_2_Cl_2_, 0°C→r.t., 89%; (iv) Et_3_N·3HF, THF, r.t.; (v) (iPr_2_N)(OCNEt)PCl, diisopropylethylamine (DIEA), CH_2_Cl_2_, r.t.

We designed the RNA phosphoramidites to carry a PrOM acetal ester at the 2′-OH position, an NPPOC or thiophenyl-NPPOC (SPhNPPOC) photolabile group at the 5′-OH position, and a choice of phenoxyacetyl derivatives as protecting groups for purine nucleobases and acetyl for cytidine (Fig. 1). In so doing, the base protection strategy closely follows that of the monomers used for DNA photolithography (*41*, *42*). We initially considered a straightforward route to phosphoramidites **5a–d** and **8a–d** (Fig. 2) starting from 5′,3′-OH 2′-*O*-PrOM ribonucleosides by installing the photosensitive group on the 5′-OH position and proceeding with phosphitylation. Unexpectedly, we found that the regioselectivity of photoprotection with the (SPh)NPPOC chloroformates was very poor, yielding diasteromeric mixtures of 5′- and 3′-photoprotected species that were difficult to separate and detrimental to the overall yield. Attempts to kinetically control a preferential installation on the 5′-OH position improved the regioselectivity but at the cost of incomplete transformation. To counter the poor 5′-3′ regioselectivity, we opted for a transient protection of the 3′-OH until the 5′ position was equipped with the photosensitive group. To do so, we selected the commercially available 2′-*O*-PrOM ribonucleosides carrying a dimethoxytrityl (DMTr) group at the 5′-OH position as our starting material.

Nucleosides **1a–d** were first transformed into their 3′-silylated derivatives using TBS-Cl, and the DMTr group was removed under acidic conditions using benzenesulfonic acid, affording nucleosides **2a–d** in excellent yields (70 to 90%). Next, we installed the photolabile group using the chloroformates of NPPOC and SPhNPPOC and 1-methylimidazole, producing NPPOC- and SPhNPPOC-protected nucleosides **3a–d** and **6a–d** in 82 to 95% yield. Facile removal of the 3′-TBS group with Et_3_N·3HF yielded intermediates **4a–d** and **7a–d** (82 to 96% yield), which underwent phosphitylation following standard procedures, where phosphoramidites **5a–d** and **8a–d** could be isolated in excellent yields (71 to 95%). Notably, this route is suitable for multigram-scale synthesis and we found that the SPhNPPOC-protected intermediates required little preventive measures against exposure to ambient light, beyond masking the amber glassware in protective foil.

### Microarray synthesis

#### Coupling and degradation

**Fig. 3.**
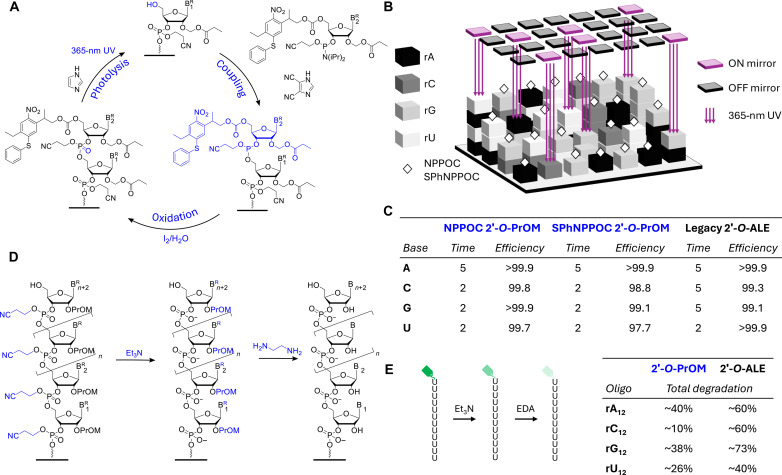
RNA microarray fabrication in situ (3′→5′) by photolithography with 2′-*O*-PrOM building blocks. (**A**) Representative coupling cycle for RNA photolithography, a repetition of three crucial steps: DCI-catalyzed coupling of the incoming RNA phosphoramidite, oxidation of the resulting phosphite triester, and photolysis of the 5′-photosensitive protecting group (here, SPhNPPOC) under 365-nm light. Reaction times are omitted for clarity. The following coupling cycle automatically starts until the desired sequences have been assembled. (**B**) Schematic illustration of the spatially selective photodeprotection in MAS. UV light at 365 nm is directed toward selected features on the array by turning the mirrors in an ON position, while unexposed features have the corresponding mirrors turned OFF. All mirrors are electronically and independently controlled inside a DMD (total of 786,432 mirrors 14 μm in size). Only exposed features (ON) can react with the next incoming RNA phosphoramidite. (**C**) Coupling conditions and stepwise yield for the 2′-*O*-PrOM RNA phosphoramidites of the 5′-NPPOC and SPhNPPOC series and compared to a prior work with 2′-*O*-ALE RNA. (**D**) RNA microarray deprotection postfabrication is a two-step process: cyanoethyl groups are first removed in triethylamine/ACN (2:3, 1.5 hours, r.t.) followed by concomitant nucleobase and 2′-OH deprotection in EDA/ethanol (1:1, 1 hour, r.t.). (**E**) RNA degradation measured during RNA deprotection [see (B)] by monitoring fluorescence loss on a terminally labeled dodecamer (5′-Cy3-rA_12_, rC_12_, rG_12_, and rU_12_). Total degradation (%) in the adjacent table is calculated after complete deprotection and relative to a stable dT_12_ dodecamer. The extent of RNA degradation is given for the 2′-*O*-PrOM and 2′-*O*-ALE monomers of the NPPOC series.

We then carried out RNA microarray photolithography with these two complete sets of RNA phosphoramidites. At the synthesis stage, adapting the MAS protocol to RNA requires few changes as the coupling cycle closely mimics that of conventional solid-phase synthesis: a standard phosphoramidite coupling step catalyzed by an activator [usually dicyanoimidazole (DCI)] followed by iodine/water-mediated oxidation of the resulting P(III) bond into P(V) and completed by a photolysis step to remove the 5′-terminal photoprotecting group (Fig. 3A). Selective photodeprotection takes place via a digital micromirror device (DMD), where the mirrors can be individually tilted in an ON or OFF position, directing 365-nm UV light onto or away from the corresponding area on the surface of the array (Fig. 3B).

We first aimed at evaluating their incorporation efficiency. To measure the stepwise coupling yield of each RNA phosphoramidite, we grow oligonucleotides from 1- to 12-nucleotide (nt) long and terminate with a fluorescent tag (fig. S2). Fluorescence intensity correlates with coupling efficiency, and the rate at which fluorescence decreases with the number of incorporations emerges experimentally as the stepwise coupling efficiency. For both SPhNPPOC and NPPOC PrOM variants, at 50 mM amidite concentration, we recorded near-quantitative coupling yields in 2 min for rC, rG, and rU, while rA consistently needed 5 min to reach completion (Fig. 3C). This already represents a substantial improvement over ALE chemistry, where three of four ribonucleotides needed at least 5 min of coupling time to achieve >99% efficiency. On average, PrOM RNA amidites couple faster and more efficiently than in solid-phase synthesis [3 min and ~98% stepwise coupling yield (*37*)], consistent with the more favorable kinetics observed on smooth surfaces relative to porous silica beads (*31*).

Postsynthesis deprotection of the RNA is performed in two separate steps (Fig. 3D), with decyanoethylation effected first using triethylamine followed by removal of the exocyclic amine and the 2′-OH protecting group with ethylenediamine (EDA). This represents a more streamlined process for handling RNA arrays than with 2′-*O*-ALE chemistry, where hydrazine-mediated levulinyl cleavage often proved to be sluggish or even incomplete. In contrast, the PrOM acetal ester is cleanly converted into its original 2′-OH group with EDA (fig. S1). PrOM monomers have the added advantage of being equipped with labile phenoxyacetyl protecting groups on the nucleobase, akin to the DNA phosphoramidites used in MAS (*19*, *41*, *42*), further extending their chemical compatibility.

Next, we monitored RNA degradation during deprotection and handling, and the fluorescently marked dodecamers used to determine coupling efficiency are ideal for that purpose as well (Fig. 3E). With ALE chemistry, we recorded strong RNA cleavage and degradation and a minimal loss of at least 40% signal relative to a Cy3-labeled dT_12_. With PrOM protection of 2′-OH, RNA degradation during deprotection is markedly reduced and ranges from 10% total signal loss for rC_12_ to a maximum of ~40% for rA_12_, with very limited degradation at the triethylamine intermediate stage (fig. S3). It is unclear why RNA stability is better controlled with the PrOM protection strategy. We would expect a propionyl ester to be more labile than its levulinyl counterpart and therefore expose the 2′-OH groups under basic conditions at a faster rate. The hydrazine treatment for ALE chains may be harsher than expected and lead to important RNA degradation.

#### Oligonucleotide synthesis, hybridization, and enzymatic treatment

**Fig. 4.**
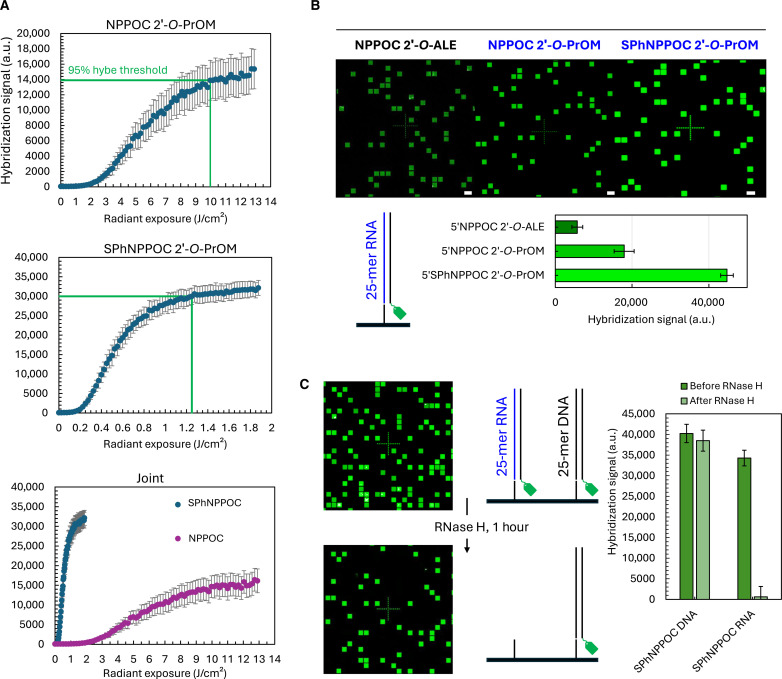
Hybridization on 25-mer RNA microarrays (5′-GTC ATC ATC ATG AAC CAC CCT GGT C). (**A**) Hybridization signals recorded on 25-mer RNA microarrays synthesized with either 5′-NPPOC (left) or SPhNPPOC 2′-*O*-PrOM phosphoramidites (middle). After each coupling, a gradient of 365-nm UV light is applied to the 5′ terminus, exposing the NPPOC group from 0 to 12 J/cm^2^ of radiant exposure (0 to 2 J/cm^2^ for SPhNPPOC). The rightmost diagram plots both SPhNPPOC and NPPOC hybridization curves. In green, the radiant exposure required to cross the 95% signal threshold. Hybe, hybridization. (**B**) Fluorescence signals recorded after hybridization with a complementary DNA oligonucleotide carrying a Cy3 fluorescent tag onto three RNA microarrays fabricated with either 5′-NPPOC/SPhNPPOC 2′-*O*-PrOM or 2′-*O*-ALE phosphoramidites. Coupling time was 2 min for PrOM and 5 min for ALE phosphoramidites (except rU-ALE, 2 min). Scan excerpts for RNA arrays were made with each of the three sets of RNA phosphoramidites, representing ~2% of the total synthesis area. Scale bars, 150 μm. Contrast and brightness have been adjusted to better illustrate differences in hybridization efficiency. Hybridization signals are given in arbitrary units (a.u.) (max. 65,536) and as the average of ~2000 measurements (replicates per array). (**C**) Hybridization signals recorded on microarrays supporting DNA and RNA versions of the same 25-mer sequence and synthesized with 5′-SPhNPPOC DNA and 2′-*O*-PrOM phosphoramidites. The DNA/RNA microarray was hybridized with a complementary DNA sequence (“before RNase H” in the adjacent plot) and then treated with RNase H (1 hour, 37°C). RNA features completely disappear after enzymatic treatment (scan excerpts before and after RNase H), translating into very low hybridization signals for RNA features (“after RNase H” in the adjacent plot), while the fluorescence intensity of DNA features remains unchanged. Error bars are SD.

With fast and efficient coupling along with limited degradation, we sought to synthesize longer, mixed-base RNA sequences on microarrays. The following 25-mer sequence 5′-GTC ATC ATC ATG AAC CAC CCT GGT C is our laboratory workhorse and is used to monitor, verify, and compare synthesis quality across arrays and chemistries. To do so, a Cy3-labeled complementary strand hybridizes to the array-bound sequence, and the surface is scanned for fluorescence after excitation at 532 nm, the signal intensity being a good indicator of synthesis efficiency. We used hybridization here not only to verify RNA synthesis but also to confirm chemical identity and identify the ideal UV exposure parameters. To measure the required exposure time—or photolysis efficiency—of the NPPOC/SPhNPPOC groups, we synthesize the 25-mer RNA by exposing the array to a gradient of 365-nm UV light after each phosphoramidite coupling. Complete NPPOC/SPhNPPOC photolysis liberates all possible 5′-OH functions of the growing strand and yields full-length oligonucleotides at all possible surface sites. On the other hand, incomplete photolysis prevents coupling of the next incoming nucleotide. Strongly exposed features produce the strongest hybridization signals. We monitor the increase in signal intensity as a function of stepwise radiant exposure to 365 nm, from 0 to 12 J/cm^2^ and from 0 to 2 J/cm^2^ for the NPPOC and SPhNPPOC groups, respectively, based on earlier works in the DNA series (*19*, *40*). The photodeprotection efficiency curves are shown in Fig. 4A. We found that hybridization signals sharply increase with increasing UV exposure, but the onset is delayed until ~2 J/cm^2^ for NPPOC and ~0.2 J/cm^2^ of exposure for SPhNPPOC. This delay likely stems from the massive accumulation of deletion errors at low radiant exposure, resulting in mostly truncated shortmers that cannot hybridize properly to a 25-mer strand and survive the hybridization conditions and subsequent stringent washes. Delay notwithstanding, hybridization curves approach first-order kinetics of photolysis (*40*), with the signal increase rapidly plateauing. The transition to plateauing regime is estimated at 95% of the maximum hybridization signal, which NPPOC RNA crosses at ~10 J/cm^2^ of radiant exposure and SPhNPPOC RNA at 1.25 J/cm^2^. The photosensitivity of the 5′-SPhNPPOC group is therefore 8× that of the 5′-NPPOC group, a somewhat smaller ratio than in the DNA series but a major improvement for microarray synthesis time. Unexpectedly, however, both NPPOC and SPhNPPOC groups on RNA nucleotides appear to require longer exposure to cross the same 95% threshold, when only 6 and 0.5 J/cm^2^ were needed for NPPOC and SPhNPPOC DNA, respectively. While we cannot yet explain this difference in reactivity, we observed similar notable photolysis behavior on 3′-OH photoprotection and for the l-DNA enantiomer as well (*41*, *43*).

The fluorescence signals of hybridization are profoundly different between the 25-mer RNA synthesized with NPPOC and SPhNPPOC RNA monomers. With NPPOC photochemistry, we consistently reach 15,000 to 17,000 units of fluorescence, but the SPhNPPOC RNA variant far outshines it and delivers very strong signals well above 35,000, sometimes up to 45,000 units, close to what can be reached at most with DNA chemistry. Crucially, both sets of PrOM amidites show superior hybridization efficiency to our legacy ALE system, which only yields <6000 units (Fig. 4B). In all cases, the signals are homogeneously distributed across the surface. DNA and RNA versions of the same sequence can be synthesized simultaneously on the same microarray, with very bright and almost 1:1 signal intensity hovering around 40k (Fig. 4C).

The total number of features selected for synthesis influences this signal ratio. Unexpectedly, with a 50% total use of the surface area for DNA and RNA synthesis, DNA hybridization signals are almost three times higher than those for RNA (fig. S4). With 10 to 20% total use (as in the scan excerpts in Fig. 4, B and C), RNA hybridization signals match those of DNA. This was observed for both the NPPOC and SPhNPPOC series. The ratio improves in favor of RNA upon increasing the concentration of complementary oligo. In our opinion, this phenomenon can only be explained if we assume that the kinetics of DNA and RNA hybridization on microarrays are markedly different, with DNA hybridization occurring at a much faster rate and greatly depleting the pool of available complement. Additional work would be required to support this hypothesis.

To confirm nucleic acid identity, we subjected the hybridized array to an enzymatic degradation assay with ribonuclease (RNase) H. After 1 hour at 37°C, the RNA features lost, on average, 95% of their original signal, while the DNA features remained practically unperturbed. This indicates that the surface-bound RNA:DNA hybrid is a substrate for RNase H and that the 2′-OH protecting groups were correctly removed.

#### Nucleic acid libraries and time considerations

**Fig. 5.**
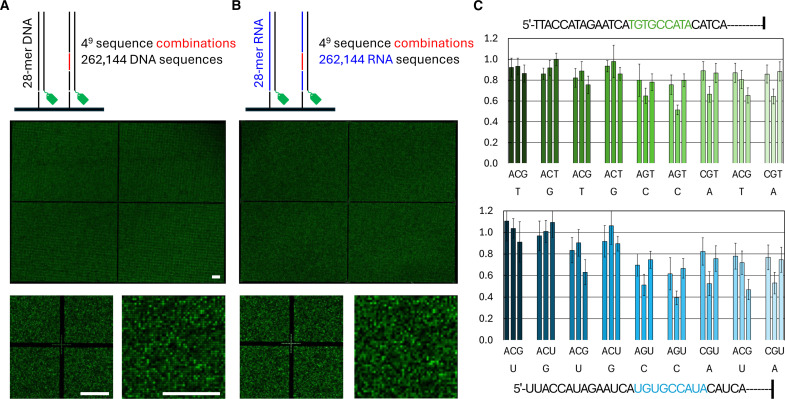
Photolithography fabrication time and array complexity. A 28-mer sequence (5′-TTA CCA TAG AAT CAT GTG CCA TAC ATC A) was synthesized as a DNA (**A**) and RNA (**B**) microarray with 5′-SPhNPPOC DNA and 2′-*O*-PrOM phosphoramidites. A middle section of nine consecutive nucleotides (TGT GCC ATA; in red) was transformed into a permutable region (*NNN NNN NNN*) of 4^9^ (262,144) sequence combinations. The complete permutation library is synthesized in DNA and RNA array format, with single-mirror feature size and three replicates per sequence per array and hybridized to a fluorescently labeled oligonucleotide complementary to the original 28-mer sequence. Scan excerpts represent the full synthesis area (top) and zoomed-in variants (1% view, bottom left and 0.2% view, bottom right). The middle cross is used for scan alignment. Scale bars, 350 μm. (**C**) Effect on hybridization signal of every possible point mutation at each position along the 9-nt stretch in the 28-mer. The fluorescence intensity of hybridization is normalized to 1 (full-match), 0 being the lowest recorded signal (scrambled 9-nt sequence). Error bars are SD. Hybridization (top) to the DNA microarray and (bottom) to the RNA microarray.

We next attempted to prepare a much more complex microarray that contains over 262,000 unique sequences. To do so, we chose a 28-mer sequence and selected a region of nine consecutive nucleotides where all possible nucleotide permutations are generated, equivalent to 4^9^ (262,144) permutations. This highly complex design engages all mirrors of the DMD and populates the synthesis area with sequences occupying the smallest feature size (14 × 14 μm^2^) and the shortest space in between (~1 μm). The synthesis of this high-density microarray needs a total of 67 cycles due to the presence of the variable region where the MAS setup is forced to introduce an A, C, G, and T coupling cycle at each of the nine permutable locations. We prepared the DNA and RNA versions of the same sequence library and proceeded with a single hybridization with a labeled strand complementary to the original, unchanged 28-mer. The hybridization scans in Fig. 5 show that high-density RNA synthesis is possible, yielding fluorescence signals of similar intensities to those of the DNA array counterpart and clearly resolved features in both cases. There is good correlation in the distribution of fluorescence values between the DNA and RNA libraries and even greater correlation between two separate hybridization assays on two RNA microarrays (fig. S6), indicating high reproducibility of the data even between two nucleic acid families. Likewise, the distribution of sequences in the total range of fluorescence is almost identical between the DNA and RNA versions of the library (fig. S7), with only ~30 sequences occupying the 90th percentile of signal intensities.

Analysis of the hybridization data at the level of single point mutations on the 9-nt sequence (5′-TGTGCCATA) indicates that both position and nature of the mismatch influence hybridization efficiency. The first two nucleotides from the 5′-end appear relatively insensitive to the introduction of mismatches (Fig. 5C) and yield hybridization signals close to that of the full-match. On the other hand, a mismatch at any of the two consecutive Cs results in a minimum of 30% loss of signal and to ~60% loss in the case of a G·G mismatch. A similar analysis on the DNA library reveals a generally comparable picture of the effect of point mutations; that is to say, the 3′-end of the section is more affected by the presence of a mismatch than the 5′-end. On average, the fluorescence intensity for single mismatches in RNA is ~10% lower than for the corresponding mismatched DNA sequence, indicating greater sensitivity of the RNA library to sequence imperfections.

**Fig. 6.**
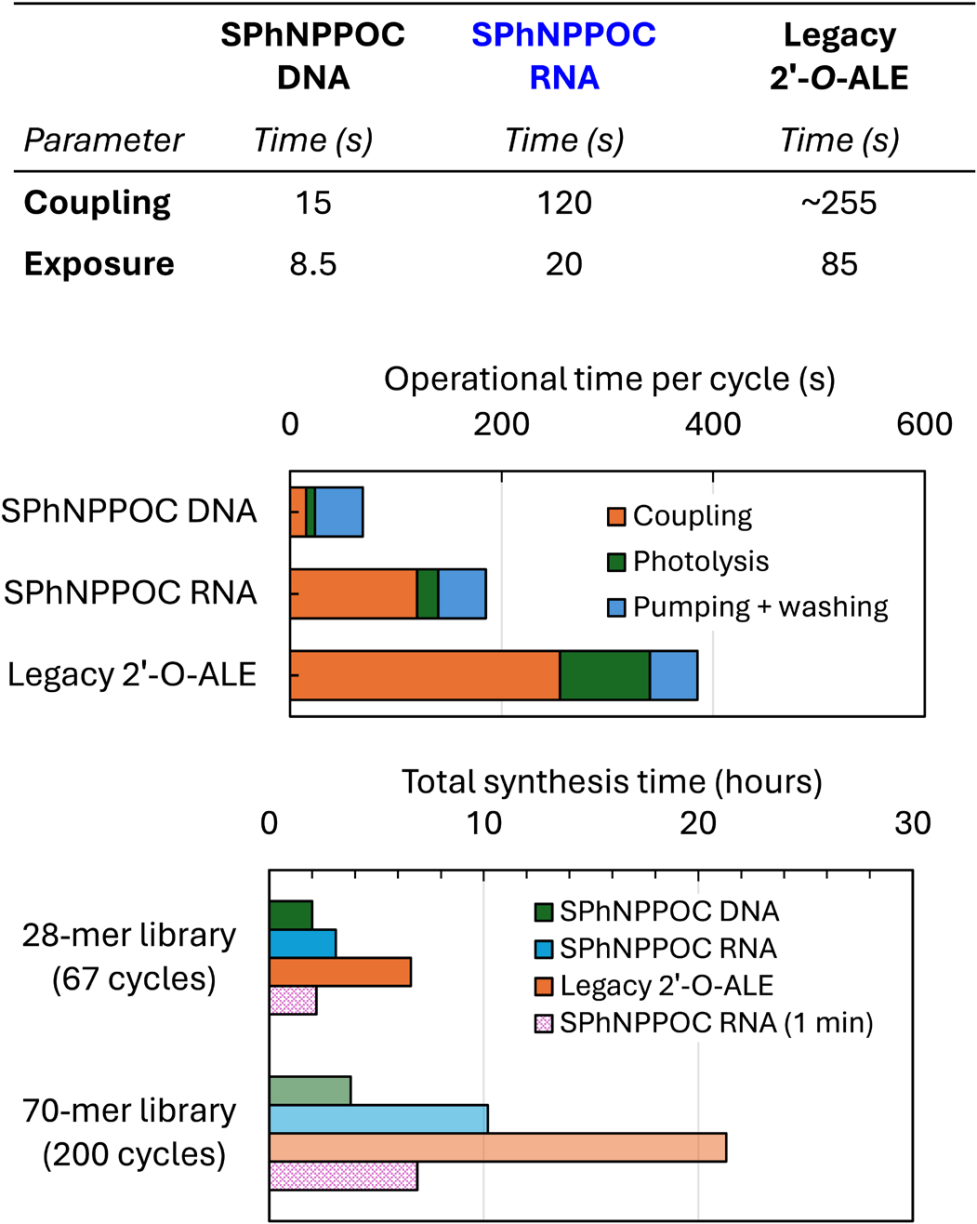
Considerations over the photolithographic fabrication time with improved RNA monomers. The top table summarizes the coupling and photolysis times selected to yield optimal hybridization signals in the DNA and RNA (PrOM/ALE) array series. The graphs below concatenate these individual values (coupling, photolysis, oxidation, washing, and drying) into an operational cycle time and a total fabrication time for the library of 28-mer (67 synthesis cycles). A fictive fabrication time for a microarray requiring 200+ synthesis cycles is also calculated. In dotted pink is the total synthesis time if 5′-SPhNPPOC 2′-*O*-PrOM RNA phosphoramidites were coupled for 1 min only.

For nucleic acid libraries of this complexity and length, the total fabrication time of the corresponding microarray becomes a factor that can affect surface integrity as prolonged exposure to reagents, organic solvents, and water visibly etches away the functional silane layer between the glass and the oligonucleotide (*34*, *44*). At the extreme range of this scale, it becomes experimentally impractical and RNA microarray synthesis with ALE monomers has quickly reached this threshold. The total synthesis time for the 28-mer DNA library using SPhNPPOC DNA phosphoramidites was around 2 hours (Fig. 6) and over 6 hours with 2′-*O*-ALE phosphoramidites. With SPhNPPOC 2′-*O*-PrOM RNA phosphoramidites, on the other hand, we were able to halve the total fabrication time down to slightly over 3 hours and can now approach DNA synthesis levels. This difference in synthesis time between our two sets of RNA monomers does not seem to fully reflect the improvements in coupling and photolysis. We summarize the coupling (c) and exposure (e) parameters in Fig. 6. With DNA, coupling is 15 s and exposure is 8.5 s; with SPhNPPOC PrOM RNA, the c/e parameters are 120 s/20 s and, for NPPOC ALE RNA, c/e is 255 s/85 s. This could be interpreted as a ~2.5× time gain per cycle, but this gain is only additive and indicates that an average of ~200 s is saved per cycle with PrOM RNA monomers. In the context of the 28-mer library, the first five amidite couplings correspond to the dT_5_ linker, so the total time saving applies to the remaining 62 RNA couplings, representing just over 3 hours (200 × 62). A better and perhaps more complete representation of the time gain is in considering the “operational cycle time,” which PrOM RNA amidites complete in ~180 s and ALE RNA amidites in ~390 s. The synthesis cycle contains additional reactions like oxidation along with multiple washing steps in between, and these commands have an assigned operational time. While these operations can be shortened to a certain extent (*45*), they are an unavoidable part of the total synthesis time but are, to the best of our knowledge, independent of nucleic acid chemistry. In the context of accelerated RNA synthesis, it would therefore be more appropriate to describe these advances as striving after the quality and throughput in DNA photolithography. If we extrapolate from these microarray fabrication times toward extremely complex sequence libraries with each oligonucleotide 70-nt long, then we expect to need a total of ~200 coupling cycles. In the abovementioned operational cycle, DNA synthesis is completed after ~4 hours, while RNA synthesis would require ~10 hours with PrOM monomers and almost an entire day (22 hours) with ALE amidites. It would seem fitting to draw the barrier of convenience and practicality at this stage.

Additional time can be saved by limiting the RNA coupling time to 1 min. We attempted to synthesize the 25-mer in Fig. 4 with SPhNPPOC RNA phosphoramidites with a 1-min coupling time for each A, C, G, and U monomer. We found that the hybridization signals were markedly lower (16,000 versus 45,000 units; fig. S5) but within the range of what was possible to obtain with NPPOC PrOM phosphoramidites, that is, substantially stronger signals that is possible to get with ALE chemistry. With a 1-min coupling time, RNA microarray synthesis with 200 coupling cycles would now be shortened to ~6 hours (Fig. 6, data in dotted pink).

#### Fluorogenic RNA aptamers

**Fig. 7.**
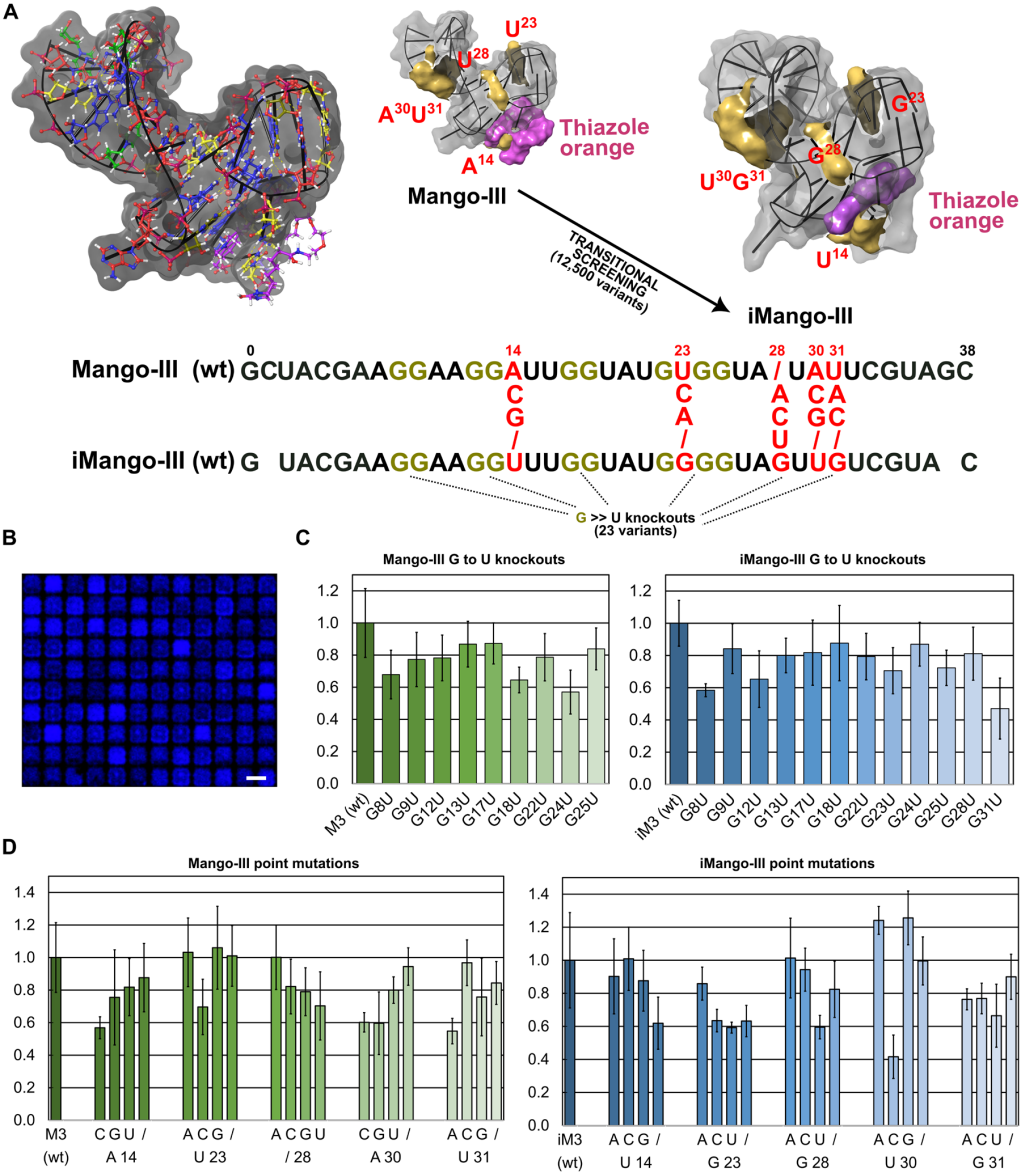
Fluorogenic RNA aptamers on microarrays. (**A**) Wireframe and surface representation of the Mango-III crystal structure [Protein Data Bank (PDB): 85UJ] and iMango-III RNA aptamers (PDB: 6E85) in complex with TO. Regions of interest are colored in yellow, and TO is in purple. The RNA library synthesized as a microarray consisted in screening the permutational landscape separating the two Mango variants (12,500 combinations), with each differing position in the central fold numbered and labeled in red. Guanine residues marked in gold and red were used for G→U substitutions. (**B**) Scan excerpt of the aptamer array after binding to 100 nM TO and scanning at 488 nm. Excerpt is <0.01% of the total synthesis area. Scale bar, 100 μm. (**C**) Effect of G→U substitutions at any of the guanine residues of the central fold in terms of loss/gain of fluorescence, relative to wild type (wt). Error bars are SD. (**D**) Effect of point mutations at any of the red-labeled nucleotides in Mango-III (data in green) and in iMango-III (data in blue). “/” refers to a deletion. Error bars are SD.

To verify the functionality of our RNA array platform with our set of nucleoside building blocks, we set out to synthesize RNA aptamers that can bind to fluorogenic dyes. Fluorogenic aptamers can display intense fluorescence emission upon formation of the aptamer·dye complex and have recently emerged as a useful class of molecules to track RNA in vivo (*46*). Among the many such aptamers whose names frequently recall a greengrocer’s stall, the RNA Mango and its derivatives are particularly attractive with low nanomolar affinity for thiazole orange (TO) and quantum yields (φ) reaching up to ~0.5 (*47*–*49*). We decided to focus on the Mango-III sequence and its reselected variant iMango-III (Fig. 7A). The two aptamers, 38- and 39-nt long, differ by a mere five nucleotides and we wanted to study how each of these positions is sensitive to change through systematic mutations. In addition, their respective double-stranded stem sequences also differ, so each mutant was also synthesized over a Mango-III or iMango-III stem. Along with G→U point mutations specifically directed at the two G tetrads, this set of sequences amounted to ~12,500 aptamer variants. After RNA microarray synthesis and deprotection, the Mango array was folded and subjected to binding to TO followed by scanning at 488-nm excitation wavelength (Fig. 7B).

Both wild-type Mango-III and iMango-III aptamers were found to bind TO with characteristic high fluorescence enhancement and a signal-to-noise ratio approaching 17:1 at 100 nM TO, reaching >34,000 fluorescence units for both reference aptamers. Looking at point mutations introduced within any of the five nucleotides that separate Mango-III from iMango-III (U14, G23, G28, U30, and G31), we find position and nucleobase dependence in how a mutation affects the fluorescence signal (Fig. 7D). For instance, in iMango, the U30C mutant loses 60% fluorescence while both U30A and U30G mutants increase fluorescence by ~20%. Mutating or deleting G23 leads, on average, to a loss of ~40% fluorescence. Deleting any of these five nucleotides is detrimental to the signal except for U30 where iMango seems unaffected. From the point of view of Mango-III, these five nonoverlapping positions (A14, U23, Ø28, A30, and U31) also appear to be sensitive to change. A loss of fluorescence reaching >40% was recorded for either A14C, A30C, A30G, or U31A mutants. On the other hand, modifying the U19 position (including a deletion) barely altered fluorescence, with the exception of U23C (~30% signal loss). The introduction of an additional nucleotide between A27 and U28, en route towards iMango-III, negatively affects the fluorescence signal in all but the A case. In iMango-III, this inserted nucleotide is a guanine (G28). All single point mutants of Mango-III whose change corresponds to the nucleotide in iMango-III (A14U, U23G, Ø28G, A30U, and U31G) result in a loss of signal, indicating that a single change is not sufficient to obtain a functional parent of iMango-III. Looking at the entire permutational landscape across all five positions (5^5^ or 3125 sequences), we found that a vast majority of mutants exhibit lower fluorescence signals than the wild-type version (fig. S8). However, a few mutated candidates far outshine Mango-III or iMango in fluorescence intensity. The A14/Ø23/G28/G30/G31 mutant, for instance, shares similarity with Mango-III (A14) and iMango-III (G28 and G31) and delivers an additional 20,000 fluorescence units (54,000 versus 34,000), which is very close to saturation levels. Whether this result can be attributed to stronger affinity and/or increased fluorescence enhancement would require further analysis and validation. Knockout variants where any guanine in the core region has been replaced with uracile consistently show a decrease in fluorescence intensity (from 17 to 50% loss; Fig. 7C), regardless of its putative role in the fluorogenic aptamer.

## DISCUSSION

A major accomplishment of this work is in greatly accelerating the RNA synthesis time with a set of RNA phosphoramidites carrying a small 2′-protecting group and a highly photosensitive 5′-protecting group. Independently, these two chemical changes have allowed to overcome the throughput limitations of previous RNA synthesis while increasing quality and hybridization efficiency. However, while we reach ever closer to DNA, the gap between DNA and RNA synthesis times has not been completely filled. For hybridization-only assays, shortening the coupling time at the cost of reduced signal should prove to be an acceptable trade-off. Beyond this fairly simple modification of the coupling parameter, further compression of the coupling time seems possible but would require adjustments, for instance, in the choice of activator (or coupling reagent). There are numerous tetrazole-like activators with a lower p*K*_a_ (where *K*_a_ is the acid dissociation constant) than DCI, which should translate into faster coupling times. We had previously observed unacceptable surface artefacts with tetrazole activators (*45*), but improvements in surface chemistry (*44*) encourage us to revisit this idea. An increase in ambient temperature will positively affect the kinetics of coupling, but our photolithography setup is sensitive to temperature changes, limiting the range of suitable fabrication conditions. A radical alternative would be to study another set of RNA amidites. From the point of view of steric hindrance, the PrOM group is one of the smallest options available for 2′-OH protection, the TBS, triisopropylsilyloxymethyl (TOM), and bis(acetoxyethoxy)methyl ether (ACE) groups occupying a much larger space (*50*–*52*). The only reasonable challenger sizewise would be the cyanoethoxymethyl (CEM) group (*53*, *54*), but its lability under basic conditions should be investigated.

Regarding quality and signal strength, there is another 5% in hybridization efficiency that can be garnered by exposing the NPPOC or SPhNPPOC groups to a larger amount of UV light, but this increase appears minimal compared to the increase in exposure time (Fig. 4A). For NPPOC, reaching 99% efficiency (13 J/cm^2^) would require close to 3 min of UV exposure, and for SPhNPPOC, the 99% threshold is crossed at a radiant exposure of ~2 J/cm^2^ or 29 s. When time consideration is not an issue and outmost nucleic acid quality is requested, such as for data storage and sequencing (*19*, *55*), this will be an appreciable improvement, but for hybridization assays, this sort of optimization is likely to be inconsequential. It is also worth noting that the exposure parameters were calculated based on the current output of our 365-nm UV–light-emitting diode (LED) (*56*) at ~70 mW/cm^2^ and that 140 mW/cm^2^ outputs are easily attainable. For SPhNPPOC RNA amidites, this halves the exposure time to ~10 s per cycle, but the effect is greater for NPPOC groups where the 10 J/cm^2^ mark would be reached in just 70 s.

The photolytic behavior of NPPOC and SPhNPPOC groups attached to ribonucleosides is slower than that for deoxynucleosides (required radiant exposure of 6 versus 10 J/cm^2^ for DNA and RNA in the NPPOC series; 0.5 versus 1.25 J/cm^2^ for SPhNPPOC), and this was observed in nonnatural nucleic acid microarrays as well. It should be kept in mind that what is being measured are hybridization signals (Fig. 4A) and not a direct monitoring of photorelease. Using hybridization to infer on photolysis efficiency is a very convenient assay to gauge the ideal exposure parameters because hybridization is a standard quality control for microarrays. However, the underlying physicochemical parameters of surface hybridization can differ substantially between nucleic acid families, and the presence of deletions due to incomplete photodeprotection may be more detrimental to hybridization for RNA, producing a photolysis curve that seemingly reaches a signal plateau with longer UV exposure.

RNA degradation has been noticeably decreased relative to a synthesis with ALE monomers, and it must constitute one of the reasons for the large increase in hybridization signal. With values up to 45,000 units (saturation level: 65,000) and on par with DNA signals, the extent of degradation appears manageable for mixed-base sequences. As indicated by the study of fluorogenic aptamers, degradation does not seem to impose a practical barrier to producing functional RNA arrays.

The commercially available 5′-DMTr 2′-*O*-PrOM precursors provide a useful gateway into the synthesis of 3′-photoprotected “reverse” RNA phosphoramidites, which is currently underway and is expected to become the next upgrade of the MAS chemical toolbox. These synthons would serve as an ideal starting point for the untemplated enzymatic polymerization of RNA and DNA oligonucleotides on microarrays. With the improved chemistry and photochemistry of RNA photolithography, it now becomes possible to foresee the preparation of microarrays decorated with much larger functional RNA molecules, such as tRNA, guide RNAs and RNAzymes, which were simply out of reach with ALE chemistry.

## MATERIALS AND METHODS

### Principle of MAS

MAS, or nucleic acid photolithography, follows principles that have been thoroughly described elsewhere (*19*, *25*, *31*, *42*, *56*–*58*). Briefly, microarray synthesis takes place on glass microscope slides (Schott Glass D) that have been silanized with *N*-(3-triethoxysilylpropyl)-4-hydroxybutyramide (Gelest SIT8189.5), providing hydroxyl functions onto which the first phosphoramidite can be coupled. The reaction chamber consists of two such slides separated by a 50-μm-thick Teflon gasket. To allow for reagents and solvents to flow in between the two slides, the bottom surface is first drilled with two small holes corresponding to the diameter of the tubing that attach the reaction chamber to a DNA synthesizer (Expedite 8909, PerSeptive Biosystems). The DNA synthesizer takes care of the delivery of all synthesis reagents, including DCI activator [0.25 M in acetonitrile (ACN), Sigma-Aldrich], dry (<30 parts per million H_2_O) ACN (Sigma-Aldrich L010000), oxidizer [20 mM I_2_ in H_2_O/pyridine/tetrahydrofuran (THF); Sigma-Aldrich L060084], phosphoramidites (diluted 30 mM in dry ACN for DNA and 50 mM for PrOM RNA and Cy3), and exposure solvent [1% imidazole (w/v) in dimethyl sulfoxide]. The exposure solvent fills the inner reaction chamber between the two slides during UV illumination and serves to effect cleavage of the NPPOC group from the 5′-OH position through base-mediated β-elimination after triplet sensitization of the nitrophenyl moiety (*59*). A UV-LED produces 365-nm light (Nichia NVSU333A) that is directed onto a 0.7″ XGA DMD (Texas Instruments) whose micromirrors are electronically controlled to be tilted either ON or OFF. ON mirrors reflect the incoming UV light into the reaction chamber where photochemistry takes place, OFF mirrors reflect UV light away from the reaction chamber, and the corresponding areas are therefore not exposed, thus keeping the 5′-photoprotecting group. A computer controls the tilt of each individual micromirror and manages communication between the synthesizer and the DMD to ensure synchronicity between illumination and delivery of the exposure solvent.

### Conventional DNA microarray synthesis by photolithography

DNA microarray synthesis used 5′-SPhNPPOC DNA phosphoramidites (dA^Bz^, dC^iBu^, dG^iBu^, and dT^*N*3-tertbutylbenzoyl^; kindly gifted by J. Lackey) and 5′-NPPOC DNA phosphoramidites (dA^tac^, dC^iBu^, dG^iPrPac^, and dT; ChemGenes) for DNA/RNA comparison or 5′-BzNPPOC variants (dA^tac^, dC^Ac^, dG^tac^, and dT) from Orgentis GmbH otherwise. Amidites were prediluted in dry ACN to prepare 30 mM solutions and kept diluted at −25°C with a moisture trap (Sigma-Aldrich Z509000). DNA phosphoramidites were coupled for 15 s in the presence of 0.25 M DCI activator followed by oxidation (~3 s) and exposure (1 J/cm^2^ for SPhNPPOC, 3 J/cm^2^ for BzNPPOC, and 6 J/cm^2^ for NPPOC). After synthesis, the microarray slides were deprotected in EDA/EtOH 1:1 for 2 hours at room temperature (r.t.) (overnight for SPhNPPOC amidites due to the presence of a benzoyl group on dA), rinsed with deionized water, dried in a microarray centrifuge, and stored in a desiccator until further use.

### RNA microarray synthesis

#### General

RNA 2′-*O*-PrOM phosphoramidites were not stored prediluted in ACN as for DNA monomers but rather classically weighed in and diluted in the strict minimum amount of ACN to make up 50 mM immediately prior to microarray synthesis. RNA amidites were otherwise stored as powders at −25°C.

#### Coupling efficiency determination

The determination of coupling efficiency requires both capping after coupling and a terminal labeling step to install a fluorogenic dye to the 5′-end of the oligonucleotides. In this microarray design, the coupling of the phosphoramidite to be tested was followed by a capping step. This capping step is performed by coupling a standard 5′-dimethoxytrityl-dT (DMTr-dT) after each phosphoramidite coupling. Because photolithography does not require the use of any acidic deblocking event, attaching DMTr-dT units to the 5′-end of coupling failures can be essentially seen as capping. RNA strands 1- to 12-nt long were synthesized simultaneously (fig. S2). In the case of 100% coupling efficiency, the signal intensity of the terminal dye would suffer no loss across all oligonucleotide lengths. For any other scenario, the terminal fluorescence signal would decrease exponentially as a function of the number of incorporated phosphoramidites. To observe this exponential decay, signal loss can be plotted against the number of couplings, and the mathematical equation *y* = *ae*^−*bx*^ fitted against experimental data to extract the rate of decay −*b* or coupling efficiency 1 − *b*.

As presented in fig. S2, a dT_5_ linker is first introduced everywhere on the surface of the array. Then, there was a stepwise incorporation of 0 to 12 DNA analogs of the sample to be tested (e.g., dX) followed by a stepwise incorporation of 0 to 12 RNA nucleotides (e.g., rX). Here, the dX units acted as a length corrector so that all synthesized oligonucleotides would have the same total length and the terminal dye is at the same distance from the surface regardless of the number of rX incorporation. After each phosphoramidite coupling, a capping step was introduced by coupling DMTr-dT (30 mM in ACN) for 60 s. A dT_10_ spacer was then inserted to distance the 5′-terminal dye from the 12-mer domain and prevent any sequence-dependent effect on the fluorescence signal. Last, a Cy3 phosphoramidite (LGC Biosearch) was coupled (50 mM in ACN, 2 × 5 min) to one-half of the total number of features, with the other unlabeled half serving as a negative control. A single array would be able to test the coupling efficiency of one control sample (dT) and all four rX phosphoramidites at the same time, for a total of 120 unique sequences and 120 technical replicates for each.

Following the synthesis, microarrays were directly transferred and washed in anhydrous ACN for 2 hours at r.t. with agitation. This was done to wash away most noncovalently bound Cy3. Microarrays were dried by centrifugation, washed in 0.1× saline sodium citrate (SSC) buffer for a few seconds, dried again, and scanned under appropriate laser/filter combination for Cy3 (532 nm) on a microarray scanner (GenePix 4100A, Molecular Devices). As standard deprotection reagents cause some degradation of the RNA oligonucleotides, the coupling efficiency was determined at the level of fully protected oligonucleotides.

#### RNA deprotection

After RNA synthesis, the RNA microarrays were deprotected in two separate steps. To remove the cyanoethyl groups on the phosphodiester, slides were transferred in a 50-ml Falcon tube containing Et_3_N:ACN (2:3) and incubated for 1.5 hours at r.t. under gentle agitation. Slides were washed twice in ACN with strong agitation, briefly washed in 0.1× SSC, scanned, and rewashed twice in ACN. To effect PrOM removal and base deprotection, the slides were transferred in 50-ml of EDA:EtOH (1:1) and incubated at r.t. for 1 hour. Last, slides were washed in RNase-free water, briefly washed in 0.1× SSC under strong agitation, dried in a microarray centrifuge, and stored until further use.

#### Photolysis and hybridization efficiency

To identify the ideal UV light exposure required to reach close to a maximum of hybridization signal, we used a gradient of UV exposure at each photodeprotection step while simultaneously attempting to grow a 25-mer oligonucleotide (5′-GTC ATC ATC ATG AAC CAC CCT GGT CTA). After each nucleotide coupling, we introduced a light exposure gradient consisting of 75 equal time intervals. Depending on the 5′-photosensitive group, this gradient ranged from either 0 to 13.6 J (NPPOC) or from 0 to 2 J (SPhNPPOC). The exposure interval used in this assay was ~3.2 s and was determined as the time that had elapsed between two consecutive digital masks sent to the DMD. Because of the very high sensitivity of SPhNPPOC groups to 365-nm light, the power output of UV-LED was reduced by stepping down the current from 3.2 to 0.4 A. This made it possible to keep the same time interval between steps and the same number of data points for both NPPOC and SPhNPPOC exposure gradients. In such an assay, there were >200 technical replicates for each of the exposure interval per array. DNA and RNA synthesis proceeded under the conditions described in the corresponding section (30 mM and 15 s coupling for DNA and 50 mM and 2 min coupling for RNA). After DNA or RNA synthesis, slides were deprotected under the conditions described above and hybridized against Cy3-labeled complementary sequence as described in the next section.

For microarray syntheses that contained a single sequence in DNA, RNA, or DNA and RNA format (Fig. 4, B and C), the same 25-mer sequence was synthesized in ~2000 replicates randomly scattered across the surface of the array. For RNA synthesis with NPPOC 2′-*O*-ALE phosphoramidites (ChemGenes), a 5-min coupling time was selected for rC, rG, and rA and 2 min for rU, along with 6 J/cm^2^ of radiant exposure. After synthesis, DNA, RNA, or DNA/RNA arrays were deprotected as described above. For the hybridization against the labeled complementary strand, a self-adhesive hybridization chamber (Grace Biolabs SA200) was placed on the microarray slides and filled with 300 μl of hybridization buffer. To prepare the hybridization buffer, a solution of 150 μl of 2× MES buffer (100 mM 2-morpholinoethanesulfonic acid, 1 M Na^+^, 20 mM EDTA, and 0.01% Tween 20), 110 μl of nuclease-free water (Carl Roth), 13.3 μl of acetylated bovine serum albumin (10 mg/ml, Promega), and 26.7 μl of 100 nM 5′-Cy3 complementary probe (5′-Cy3 GAC CAG GGT GGT TCA TGA TGA TGA C) was mixed in a 1.5-ml sterile microcentrifuge tube (Eppendorf). The final concentration of the probe was 10 nM. The hybridization solution was pipetted into the hybridization chamber, and the microarray slides were placed in a hybridization oven (Boekel Scientific) at 42°C for 2 hours with rotation. Following incubation, the microarray slides were washed with a series of standardized buffer washes of decreasing salt concentration. First, the microarrays were washed in a nonstringent wash buffer (0.9 M NaCl, 0.06 M sodium phosphate, 6 mM EDTA, and 0.01% Tween 20) with strong agitation for 2 min. They were transferred to a stringent wash buffer (100 mM MES, 0.1 M NaCl, and 0.01% Tween 20) and washed for 1 min with strong agitation. Last, the microarrays were briefly washed in a final wash buffer (0.1× SSC) for 5 s with strong agitation, dried with centrifugation, and scanned at 532 nm in a microarray scanner at 5-μm resolution.

#### RNase H degradation assay

To confirm the identity of the RNA oligonucleotides synthesized on the array, an enzymatic degradation assay with RNase H was carried out. We used the microarray that was designed to contain both DNA and RNA versions of the 25-mer oligonucleotide and proceeded with synthesis, deprotection, and hybridization as described above. Following scanning, a self-adhesive hybridization chamber was applied on the hybridized microarray and filled with 300 μl of RNase H buffer [10 mM KCl, 20 mM tris-HCl, 10 mM (NH_4_)_2_SO_4_, 2 mM MgSO_4_, 0.1% Triton X-100 (pH 8.8), and RNase H 5 μl @ 5 U/μl; New England Biolabs]. Slides were incubated for 1 hour at 37°C without rotation. The hybridization chamber was removed, and the microarray slides were washed briefly in the final wash buffer, dried by centrifugation, and rescanned again at 532 nm in a microarray scanner.

#### Sequence library synthesis

A 28-mer sequence (5′-TTACCATAGAATCATGTGCCATACATCA) was synthesized in DNA and RNA format along with sequence permutations. The sequence permutations are localized within a 9-nt long section of the 28-mer, taking the form 5′-TTACCATAGAATCA*NNNNNNNNN*CATCA where *N* corresponds to any nucleotide. The permutation library therefore contains 4^9^ unique sequences (262,144), which were all synthesized simultaneously on a single array. To achieve this density, each sequence was synthesized on a single-mirror feature size, with two technical replicates randomly distributed across the surface of the array, for a total of 524,288 features. To use up the rest of the available features (~240,000), a selected number of sequences were synthesized with a greater number of replicates, e.g., 7000 replicates for the original 28-mer, ~7500 replicates for sequences containing additional or fewer nucleotides in the randomized section, etc. The full list is available in (*32*). Following standard DNA or RNA deprotection and hybridization conditions as described above, the oligonucleotides were incubated with a Cy3-labeled DNA complementary probe of sequence: 5′-Cy3 TGATGTATGGCACATGTATTCTATGGTTTAA-3′. As described previously, microarrays were washed in three washing buffers, dried, and then scanned on a microarray scanner at 2.5-μm resolution (GenePix 4400, Molecular Devices) at 532-nm excitation.

#### Fluorogenic aptamers

The sequences of the 38-mer Mango-III (5′-GCUACGAAGGAAGGAUUGGUAUGUGGUAUAUUCGUAGC-3′) and the 37-mer iMango-III variant (5′-GCUACGAAGGAAGGUUUGGUAUGGGGUAGUUGUCGUAGC-3′) were aligned and subjected to transitional mutagenesis at five misaligned positions within the nonstem central regions. The calculated permutations included adenine (A), cytosine (C), guanine (G), uracil (U), and deletions (/), encompassing the entire permutation space between Mango-III and iMango-III, resulting in 3125 possible variants (5^5^). These variants were synthesized with all four possible stem combinations, yielding a total of 12,500 tested variants. In addition, the conserved guanines were individually mutated to uracile in 23 knockout variants. Overall, >13,000 unique RNA sequences were synthesized simultaneously, each represented by at least five technical replicates randomly distributed across the array. Each feature measured 64 μm^2^, with a 16-μm space separating adjacent features. The synthesis and deprotection were carried out according to the methodology described in the synthesis section. For aptamer folding, the deprotected microarrays were incubated under a temperature gradient from 75°C to r.t. for 2 hours with rotation in 50 ml of TO buffer (140 mM KCl, 1 mM MgCl_2_, 10 mM NaH_2_PO_4_, and 0.5% Tween 20). For the TO binding assay, the microarrays were incubated in 50 ml of TO buffer containing 100 nM TO for 1 min under agitation followed by a 10-s wash with shaking in 50 ml of fresh TO buffer. The arrays were dried by centrifugation and scanned at 488 nm using a microarray scanner at a resolution of 2.5 μm.

#### Data extraction and analysis

To extract fluorescence intensities from microarray scans, scan .tif files were analyzed on NimbleScan 2.1.68 (NimbleGen). Data processing was carried out using a custom-built script “Flash_v3” to automate data aggregation and statistical analysis (*60*). Further processing was done using Microsoft Excel and SigmaPlot 12.0.

## Supplementary Materials

### This PDF file includes:

Supplementary Results Figs. S1 to S8 Tables S1 and S2 Materials and Methods for Phosphoramidite Synthesis NMR Spectra of Compounds **2** to **8** Materials and Methods for Solid-Phase Oligonucleotide Synthesis
