# Supplementary material for: Accelerated, high-quality photolithographic synthesis of RNA microarrays in situ
Source: Sci Adv. 2024 Jul 31;10(31):eado6762. doi: 10.1126/sciadv.ado6762 (PMC11290486; doi:10.1126/sciadv.ado6762)
Supplement: Supplementary file 1 — Supplementary Results Figs. S1 to S8 Tables S1 and S2 Materials and Methods for Phosphoramidite Synthesis NMR Spectra of Compounds 2 to 8 Materials and Methods for Solid-Phase Oligonucleotide Synthesis [file sciadv.ado6762_sm.pdf]

Science Advances
AAAS

Supplementary Materials for

Accelerated, high-quality photolithographic synthesis of RNA microarrays in situ

Tadija Kekić *et al.*

Corresponding author: Jory Lietard, jory.lietard@univie.ac.at

Sci. Adv. **10**, eado6762 (2024)
DOI: 10.1126/sciadv.ado6762

This PDF file includes:

Supplementary Results
Figs. S1 to S8
Tables S1 and S2
Materials and Methods for Phosphoramidite Synthesis
NMR Spectra of Compounds **2** to **8**
Materials and Methods for Solid-Phase Oligonucleotide Synthesis

Supplementary Results

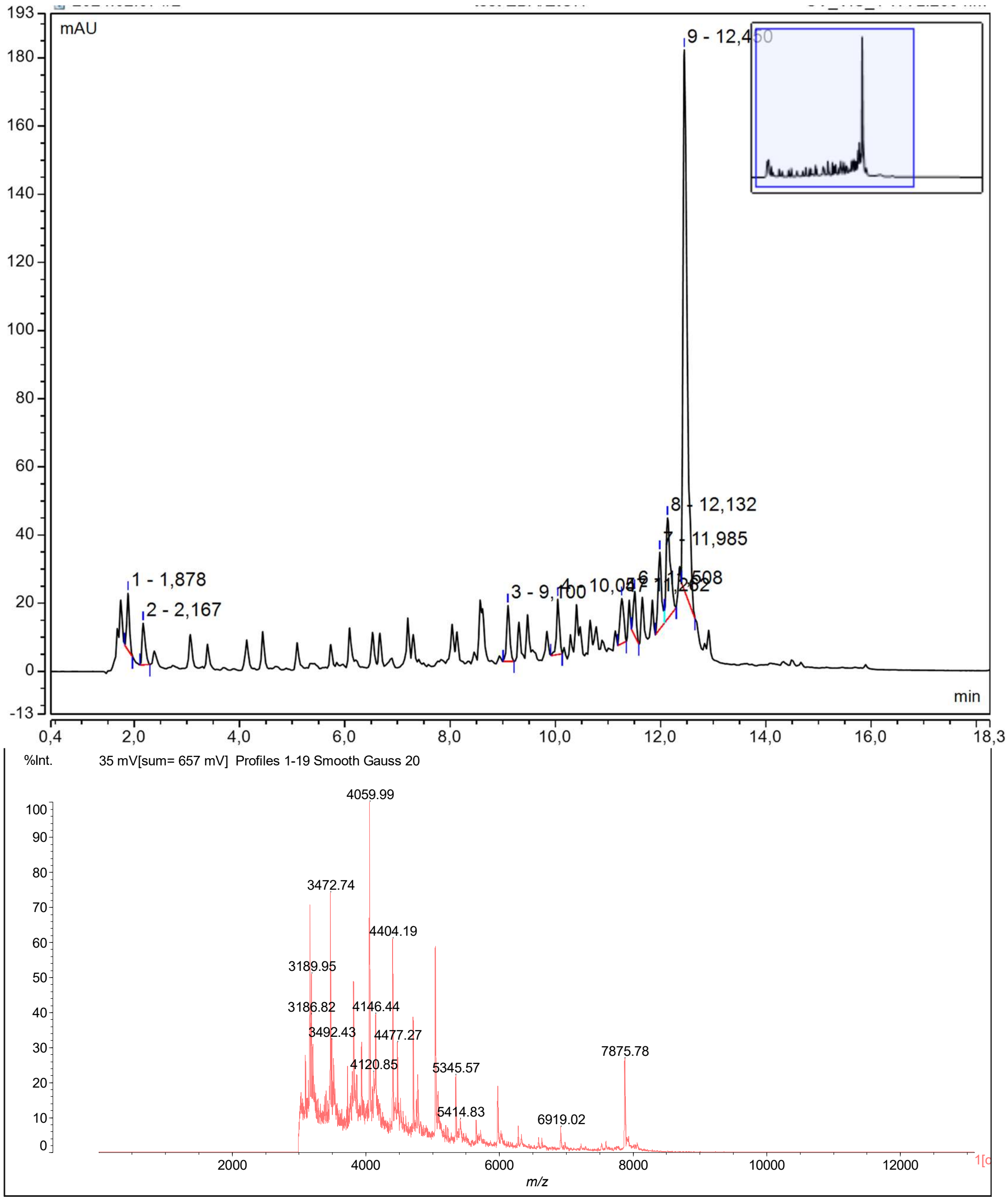

Fig. S1.

Deprotection study on a crude 25mer (5′- GUC AUC AUC AUG AAC CAC CCU GGU CUA) RNA sequence synthesized by solid-phase (top) RP-HPLC chromatogram after deprotection in Et_3N/ACN 2:3, 1h30, r.t. followed by EDA/EtOH 1:1, 1h, r.t. (bottom) MALDI-TOF MS spectrum of the same crude, fully-deprotected 25mer. M_w (expected) 7876.757 Da, (found) 7875.78 Da.

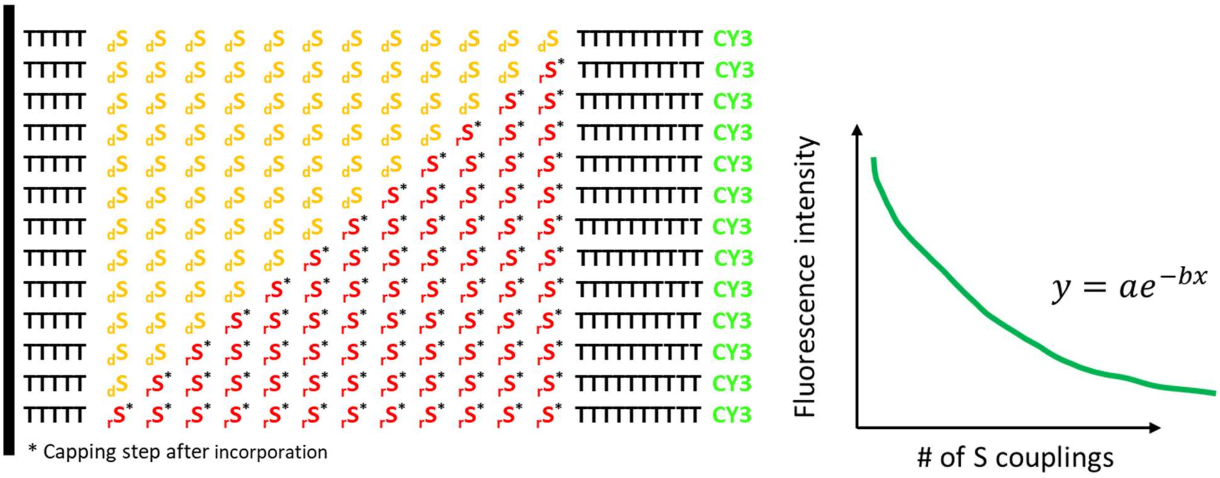

Fig. S2.

Array design for the measurement of coupling efficiency. (left) The phosphoramidite $_{r}S$ is coupled up to 12 consecutive times over a dT_5 linker, followed by a dT_{10} spacer and a terminal Cy3 coupling. A capping step (*) is introduced after each $_{r}S$ coupling in the form of an additional coupling with a DMTr-dT phosphoramidite. Since there is no acidic deblocking in photolithography, DMTr-dT essentially acts as a capping agent. To compensate for the short length of oligonucleotides with few S inserts and to ensure equal distance of the dye to the array surface, DNA nucleotides are inserted ($_{d}S$) so as to build a 12-nt long section between linker and spacer, regardless of the number of $_{r}S$ coupling events. A simple dT_{12} ($_{r}S$ = 0) is also synthesized and serves as a reference for fluorescence intensity. (right) Monitoring the decrease of fluorescence as a function of the number of $_{r}S$ coupling events equates to recording an exponential decay curve, from which the rate of decrease $(-b)$ can be converted into a stepwise coupling efficiency $(1-b)$. In this example, the decay is exaggerated purely for illustration purposes.

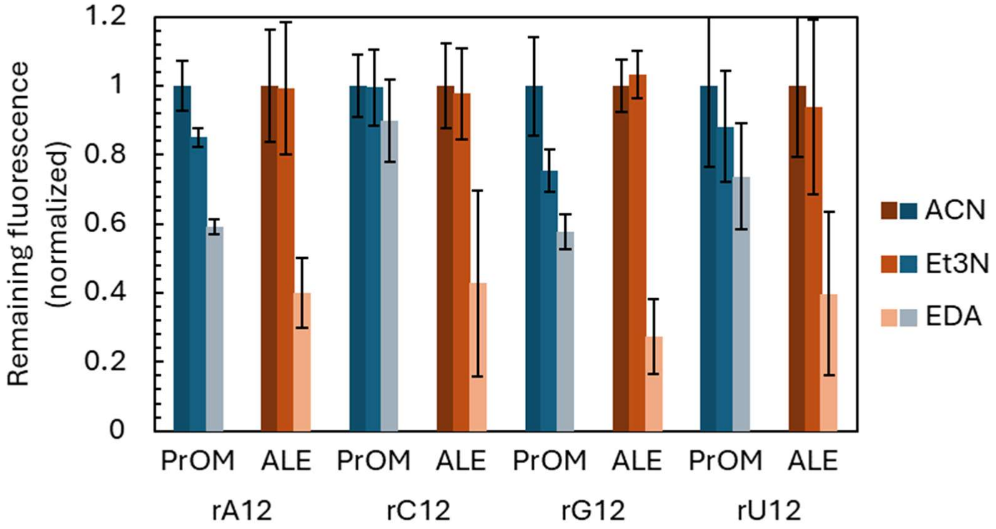

Fig. S3.

Monitoring the loss of fluorescence on 5′-labeled RNA dodecahomopolymers during RNA array deprotection. Degradation was measured for RNA oligonucleotides synthesized with NPPOC 2′-*O*-PrOM and 2′-*O*-ALE RNA amidites. Fluorescence is given as remaining signal after triethylamine and EDA treatments, normalized to that of a labeled dT_{12} control. Error bars are SD.

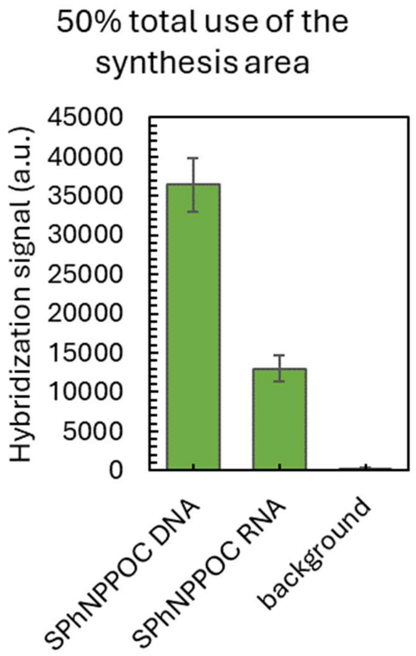

Fig. S4.

Hybridization signals recorded on a 25mer microarray synthesized with SPhNPPOC DNA and RNA phosphoramidites and where 50% of the total available features were selected for either DNA or RNA synthesis. Error bars are SD.

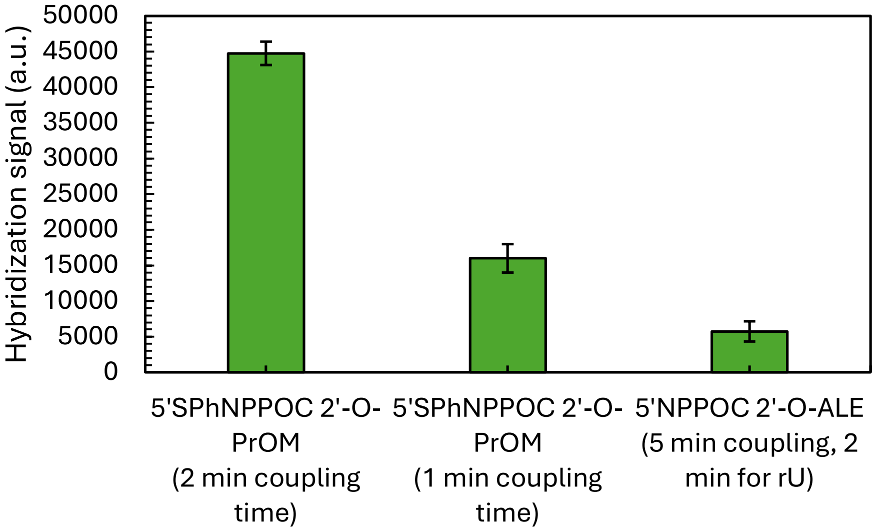

Fig. S5.

Hybridization signals recorded on a 25mer microarray synthesized with SPhNPPOC 2′-*O*-PrOM RNA phosphoramidites with 1 or 2 min coupling time, or with NPPOC 2′-*O*-ALE RNA phosphoramidites (5 min coupling time, except for rU: 2 min coupling time). Error bars are SD.

Table S1.

List of sequences (3′→5′) synthesized on a single microarray for the determination of RNA coupling efficiency. Lowercase letters are DNA nucleotides (no capping after coupling) and uppercase letters are RNA or DNA nucleotides with a capping step introduced after coupling.

Base	Sequence (3'->5')	Label
dT	ttttttttttttttttttttttttttt	Cy3
	ttttttttttttttttTtttttttttt	Cy3
	tttttttttttttttTTtttttttttt	Cy3
	ttttttttttttttTTTtttttttttt	Cy3
	tttttttttttttTTTTtttttttttt	Cy3
	ttttttttttttTTTTTtttttttttt	Cy3
	tttttttttttTTTTTTtttttttttt	Cy3
	ttttttttttTTTTTTTtttttttttt	Cy3
	tttttttttTTTTTTTTtttttttttt	Cy3
	ttttttttTTTTTTTTTtttttttttt	Cy3
	tttttttTTTTTTTTTTtttttttttt	Cy3
	ttttttTTTTTTTTTTTtttttttttt	Cy3
	tttttTTTTTTTTTTTTtttttttttt	Cy3
	ttttttttttttttttttttttttttt	-
	ttttttttttttttttTtttttttttt	-
	tttttttttttttttTTtttttttttt	-
	ttttttttttttttTTTtttttttttt	-
	tttttttttttttTTTTtttttttttt	-
	ttttttttttttTTTTTtttttttttt	-
	tttttttttttTTTTTTtttttttttt	-
	ttttttttttTTTTTTTtttttttttt	-
	tttttttttTTTTTTTTtttttttttt	-
	ttttttttTTTTTTTTTtttttttttt	-
	tttttttTTTTTTTTTTtttttttttt	-
	ttttttTTTTTTTTTTTtttttttttt	-
	tttttTTTTTTTTTTTTtttttttttt	-
rU	ttttttttttttttttttttttttttt	Cy3
	ttttttttttttttttUtttttttttt	Cy3
	tttttttttttttttUUtttttttttt	Cy3
	ttttttttttttttUUUtttttttttt	Cy3
	tttttttttttttUUUUtttttttttt	Cy3
	ttttttttttttUUUUUtttttttttt	Cy3
	tttttttttttUUUUUUtttttttttt	Cy3
	ttttttttttUUUUUUUtttttttttt	Cy3
	tttttttttUUUUUUUUtttttttttt	Cy3
	ttttttttUUUUUUUUUtttttttttt	Cy3
	tttttttUUUUUUUUUUtttttttttt	Cy3
	ttttttUUUUUUUUUUUtttttttttt	Cy3
	tttttUUUUUUUUUUUUtttttttttt	Cy3
	ttttttttttttttttttttttttttt	-
	ttttttttttttttttUtttttttttt	-
	tttttttttttttttUUtttttttttt	-
	ttttttttttttttUUUtttttttttt	-
	tttttttttttttUUUUtttttttttt	-
	ttttttttttttUUUUUtttttttttt	-
	tttttttttttUUUUUUtttttttttt	-
	ttttttttttUUUUUUUtttttttttt	-
	tttttttttUUUUUUUUtttttttttt	-
	ttttttttUUUUUUUUUtttttttttt	-
	tttttttUUUUUUUUUUtttttttttt	-
	ttttttUUUUUUUUUUUtttttttttt	-
	tttttUUUUUUUUUUUUtttttttttt	-
rC	tttttcccccccccccctttttttttt	Cy3
	tttttcccccccccccCtttttttttt	Cy3
	tttttccccccccccCCtttttttttt	Cy3
	tttttcccccccccCCCtttttttttt	Cy3
	tttttccccccccCCCCtttttttttt	Cy3
	tttttcccccccCCCCCtttttttttt	Cy3
	tttttccccccCCCCCCtttttttttt	Cy3
	tttttcccccCCCCCCCtttttttttt	Cy3
	tttttccccCCCCCCCCtttttttttt	Cy3
	tttttcccCCCCCCCCCtttttttttt	Cy3
	tttttccCCCCCCCCCCtttttttttt	Cy3
	tttttcCCCCCCCCCCCtttttttttt	Cy3
	tttttCCCCCCCCCCCCtttttttttt	Cy3
	tttttcccccccccccctttttttttt	-
	tttttcccccccccccCtttttttttt	-
	tttttccccccccccCCtttttttttt	-
	tttttcccccccccCCCtttttttttt	-
	tttttccccccccCCCCtttttttttt	-
	tttttcccccccCCCCCtttttttttt	-
	tttttccccccCCCCCCtttttttttt	-
	tttttcccccCCCCCCCtttttttttt	-
	tttttccccCCCCCCCCtttttttttt	-
	tttttcccCCCCCCCCCtttttttttt	-
	tttttccCCCCCCCCCCtttttttttt	-
	tttttcCCCCCCCCCCCtttttttttt	-
	tttttCCCCCCCCCCCCtttttttttt	-

rG	tttttggggggggggggtttttttttt	Cy3
	tttttgggggggggggGtttttttttt	Cy3
	tttttggggggggggGGtttttttttt	Cy3
	tttttgggggggggGGGtttttttttt	Cy3
	tttttggggggggGGGGtttttttttt	Cy3
	tttttgggggggGGGGGtttttttttt	Cy3
	tttttggggggGGGGGGtttttttttt	Cy3
	tttttgggggGGGGGGGtttttttttt	Cy3
	tttttggggGGGGGGGGtttttttttt	Cy3
	tttttgggGGGGGGGGGtttttttttt	Cy3
	tttttggGGGGGGGGGGtttttttttt	Cy3
	tttttgGGGGGGGGGGGtttttttttt	Cy3
	tttttGGGGGGGGGGGGtttttttttt	Cy3
	tttttggggggggggggtttttttttt	-
	tttttgggggggggggGtttttttttt	-
	tttttggggggggggGGtttttttttt	-
	tttttgggggggggGGGtttttttttt	-
	tttttggggggggGGGGtttttttttt	-
	tttttgggggggGGGGGtttttttttt	-
	tttttggggggGGGGGGtttttttttt	-
	tttttgggggGGGGGGGtttttttttt	-
	tttttggggGGGGGGGGtttttttttt	-
	tttttgggGGGGGGGGGtttttttttt	-
	tttttggGGGGGGGGGGtttttttttt	-
	tttttgGGGGGGGGGGGtttttttttt	-
	tttttGGGGGGGGGGGGtttttttttt	-

rA	tttttaaaaaaaaaaaatttttttttt	Cy3
	tttttaaaaaaaaaaaAtttttttttt	Cy3
	tttttaaaaaaaaaaAAtttttttttt	Cy3
	tttttaaaaaaaaaAAAtttttttttt	Cy3
	tttttaaaaaaaaAAAAtttttttttt	Cy3
	tttttaaaaaaaAAAAAtttttttttt	Cy3
	tttttaaaaaaAAAAAAtttttttttt	Cy3
	tttttaaaaaAAAAAAAtttttttttt	Cy3
	tttttaaaaAAAAAAAAtttttttttt	Cy3
	tttttaaaAAAAAAAAAtttttttttt	Cy3
	tttttaaAAAAAAAAAAtttttttttt	Cy3
	tttttaAAAAAAAAAAAtttttttttt	Cy3
	tttttAAAAAAAAAAAAtttttttttt	Cy3
	tttttaaaaaaaaaaaatttttttttt	-
	tttttaaaaaaaaaaaAtttttttttt	-
	tttttaaaaaaaaaaAAtttttttttt	-
	tttttaaaaaaaaaAAAtttttttttt	-
	tttttaaaaaaaaAAAAtttttttttt	-
	tttttaaaaaaaAAAAAtttttttttt	-
	tttttaaaaaaAAAAAAtttttttttt	-
	tttttaaaaaAAAAAAAtttttttttt	-
	tttttaaaaAAAAAAAAtttttttttt	-
	tttttaaaAAAAAAAAAtttttttttt	-
	tttttaaAAAAAAAAAAtttttttttt	-
	tttttaAAAAAAAAAAAtttttttttt	-
	tttttAAAAAAAAAAAAtttttttttt	-

Table S2.
List of DNA or RNA sequences synthesized for the high-density 28mer library. The permuted region is in bold, **N** letters refer to randomized, permuted RNA nucleotides

Sequence name	Sequence (5′ to 3′) N = A, C, G, U	# of replicates
Permutation library	UUACCAUAGAAUCA*NNNNNNNNN*CAUCA	2 per sequence (524 288 total)
Full-match	UUACCAUAGAAUCA**UGUGCCAUA**CAUCA	7000
ACA mutant	UUACCAUAGAAUCA**ACAGCCAUA**CAUCA	7000
U8 to A	UUACCAUAGAAUCA**UGUGCCAAA**CAUCA	2000
U8 to C	UUACCAUAGAAUCA**UGUGCCACA**CAUCA	2000
U8 to G	UUACCAUAGAAUCA**UGUGCCAGA**CAUCA	2000
A7 to U	UUACCAUAGAAUCA**UGUGCCUUA**CAUCA	2000
A7 to C	UUACCAUAGAAUCA**UGUGCCCUA**CAUCA	2000
A7 to G	UUACCAUAGAAUCA**UGUGCCGUA**CAUCA	2000
U3 to A	UUACCAUAGAAUCA**UGAGCCAUA**CAUCA	2000
U3 to C	UUACCAUAGAAUCA**UGCGCCAUA**CAUCA	2000
U3 to G	UUACCAUAGAAUCA**UGGGCCAUA**CAUCA	2000
A9 to U	UUACCAUAGAAUCA**UGUGCCAUU**CAUCA	2000
A9 to C	UUACCAUAGAAUCA**UGUGCCAUC**CAUCA	2000
A9 to G	UUACCAUAGAAUCA**UGUGCCAUG**CAUCA	2000
UGU fixed	UUACCAUAGAAUCA**UGU***NNNNNN*CAUCA	24 576
UGU random	UUACCAUAGAAUCA*NNN***GCCAUA**CAUCA	192
NN random	UUACCAUAGAAUCA**UGU***NN***AUA**CAUCA	96
NNN random	UUACCAUAGAAUCA**UGU***NNN***AUA**CAUCA	384
NNNN random	UUACCAUAGAAUCA**UGU***NNNN***AUA**CAUCA	1536
NNNNN random	UUACCAUAGAAUCA**UGU***NNNNN***AUA**CAUCA	6144

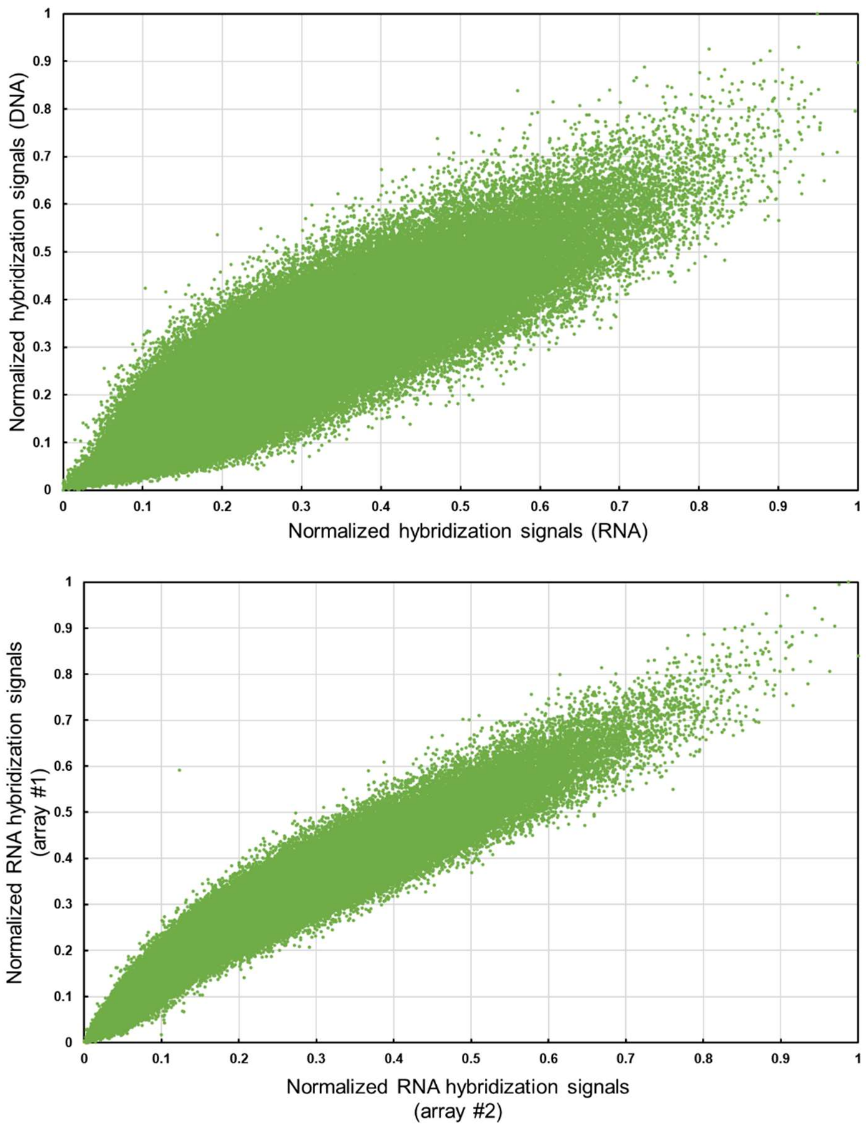

Fig. S6

Scatter plots comparing the distribution of hybridization signals to the 28mer DNA and RNA sequence libraries (Figure 4) after binding with a Cy3-labeled DNA oligonucleotide complementary to the sequence 5′-TTA CCA TAG AAT CAT GTG CCA TAC ATC A. (top) comparison of hybridization signals between DNA and RNA microarrays. (bottom) comparison between two separate RNA microarray slides of the same library and two separate hybridization assays. The fluorescence intensity has been normalized to 0 = background signal and 1 = highest recorded hybridization signal.

Fluorescence decile	# of sequences DNA	RNA
0 – 0.1	31549	31586
0.1 – 0.2	84933	67919
0.2 – 0.3	67040	67183
0.3 – 0.4	40955	51585
0.4 – 0.5	22366	28511
0.5 – 0.6	10783	11558
0.6 – 0.7	4205	3884
0.7 – 0.8	1292	998
0.8 – 0.9	277	183
0.9 – 1	35	28

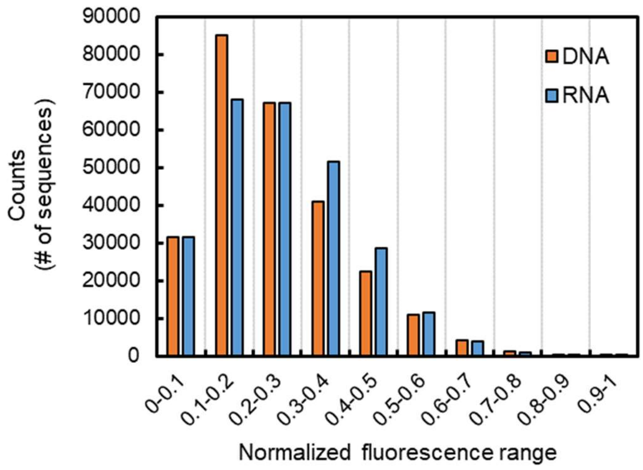

Fig. S7

Number of DNA and RNA sequences with fluorescence hybridization signals recorded within each decile of the total range of signal intensities. This range spans the lowest recorded signal (0) to the brightest hybridization signal (1) and this normalized range was split into deciles.

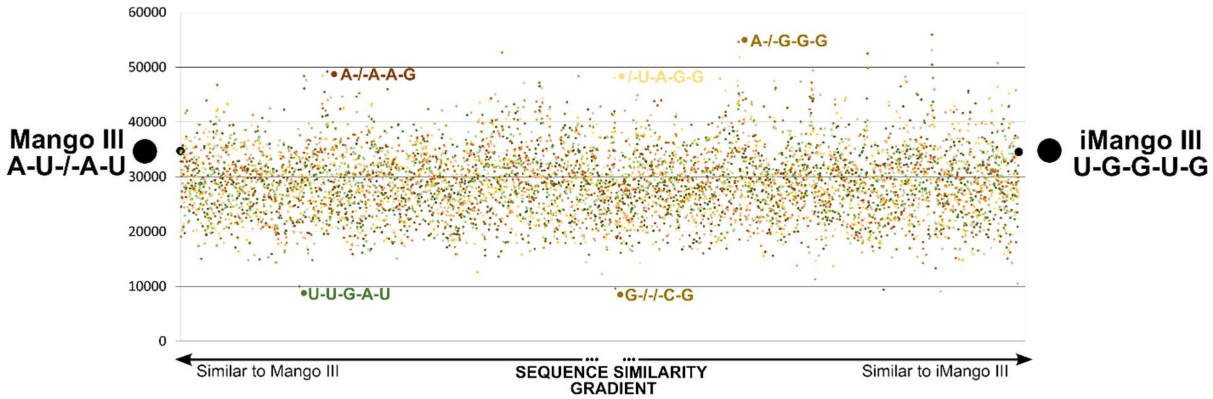

Fig. S8

Scatterplot of the distribution of fluorescence signals for all 5^5 permutations on the Mango-III/iMango-III scaffolds, after binding to 100 nM TO. The sequences are ranked on the x axis as a function of similarity to either fluorogenic aptamer. Fluorescence intensity (a.u.) is on the ordinate. A few select mutants in the lower and upper scale of fluorescence intensity have been labeled for representative purposes. The label corresponds to the nucleotide at position 14, 23, 28, 30 and 31.

Material and Methods for Phosphoramidite Synthesis

General information

All dry solvents and reagents were purchased from commercial suppliers and were used without further purification. DIEA was distilled from calcium hydride. Purifications by column chromatography were performed using Biotage Isolera 1 system with FlashPure cartridges (Buchi). 2-(2-nitrophenyl)-propyloxycarbonyl chloride (NPPOC-Cl) reagent was prepared according to the literature procedure (*RSC Advances* 2015, 5, 28344-28348, https://doi.org/10.1039/C5RA01210D) (*J. Org. Chem.* 2022, 87, 3402-3421, https://doi.org/10.1021/acs.joc.1c03006). 2-(4-ethyl-2-nitro-5-phenylthiophenyl) propyloxycarbonyl chloride (SPhNPPOC-Cl) was provided by Orgentis Chemicals. 5′-*O*-dimethoxytrityl (DMTr) 2′-*O*-propionyloxymethyl (PrOM)-protected ribonucleosides **1a-d** and 2-cyanoethyl *N,N*-diisopropylchlorophosphoramidite were purchased from ChemGenes. NPPOC- or PhSNPPOC-protected nucleosides and their respective phosphoramidites were mostly isolated as a mixture of two or four diastereomers resulting in doubling of certain signals in the ^{1}H- and ^{13}C-NMR spectra for the nucleosides, while in the case of phosphoramidites, four distinct peaks in ^{31}P-NMR were observed for certain products.

NMR experiments were recorded on Bruker 400, 500 or 600 MHz spectrometers at 20 °C. Chemical shifts are given in ppm (δ scale), coupling constants (J) in Hz. Complete assignment of all NMR signals was achieved by using a combination of ^{1}H,^{1}H-COSY, ^{1}H,^{13}C-HSQC and ^{1}H,^{13}C-HMBC NMR experiments. (o) (m) (p) refer to ortho, meta, para positions in the phenyl ring, respectively. HRMS analyses were obtained with electrospray ionization (ESI) in positive mode on a Q-TOF Micromass spectrometer.

General procedure A for synthesis of compounds 2a-d

To a solution of 5′-*O*-DMTr-2′-*O*-PrOM-protected ribonucleosides **1a-d** (1 eq) in DMF (0.3 M) were added imidazole (3 eq) and TBSCl (1.5 eq), and the reaction mixture was left to stir overnight at room temperature. The solvent was evaporated under reduced pressure and the residue was suspended in DCM and extracted three times with saturated $NaHCO_3$ solution and three times with saturated LiCl solution to remove traces of DMF. The organic layer was dried over Na_2SO_4 and evaporated under reduced pressure. The residue was dissolved in DCM/MeOH (7:3) (0.05 M) and a 10% benzene sulfonic acid (BSA) solution in DCM/MeOH (7:3, v:v) was added. The reaction mixture was stirred at room temperature until completion of the reaction, neutralized with $NaHCO_3$, extracted three times with DCM and washed with brine. The organic layer was dried over

Na_2SO_4, evaporated under reduced pressure and the crude was purified on high performance flash chromatography system on a SiO_2 column (0-10% gradient of MeOH in DCM). Fractions containing the pure products **2a-d** were collected, evaporated under reduced pressure. The residue was co-evaporated several times with DCM and pentane to provide a white foam.

3′-*O*-*tert*-butyldimethylsilyl-2′-*O*-propionyloxymethyl uridine (2a)

2a was prepared from 5′-*O*-DMTr-2′-*O*-PrOM protected uridine **1a** (3.7 g, 5.84 mmol), imidazole (1.19 g, 17.52 mmol) and TBSCl (1.32 g, 8.77 mmol) followed by detritylation with 10% BSA solution in DCM/MeOH according to **general procedure A**. Compound **2a** was isolated as a white foam (2.35 g, 90%). ^{1}H-NMR (600 MHz, DMSO-d_6) δ 11.38 (s, 1H, NH), 7.91 (d, J = 8.1, 1H, H_6), 5.85 (d, J = 4.4, $H_{1'}$), 5.68 (d, J= 10.0, 1H, H_5), 5.37 (d, J= 6.4, 1H, OCH_2O), 5.28-5.15 (m, 2H, OCH_2O, 5′OH), 4.35-4.23 (m, 2H, $H_{2'}$, $H_{4'}$), 3.86 (q, J = 3.2, 1H, $H_{3'}$), 3.66 (ddd, J= 12.0, J= 4.7, J= 3.6, 1H, $H_{5'a}$), 3.54 (ddd, J= 12.1, J = 4.5, J = 3.1, 1H, $H_{5'b}$), 2.27 (q, J = 7.5, 2H, $OCOC\mathit{H_2}CH_3$), 0.99 (t, J = 7.5, 3H, $OCOCH_2C\mathit{H_3}$), 0.88 (s, 9H, $OSiC(CH_3)_3$), 0.09 (s, 6H, $OSi(CH_3)_2$). ^{13}C-NMR (151 MHz, DMSO-d_6) δ 172.9, 163.0, 150.6, 140.5, 101.9, 87.6, 86.2, 85.1, 80.2, 70.1, 60.0, 26.5, 25.5, 18.1, 8.6, -4.9, -5.1. HRMS-ESI $[M+H]^+$ calcd for $C_{19}H_{33}N_2O_8Si$ 455.2001, found 455.2005.

3′-*O*-*tert*-butyldimethylsilyl-2′-*O*-propionyloxymethyl-*N*^{4}-acetyl-cytidine (2b)

2b was prepared from 5′-*O*-DMTr-2′-*O*-PrOM protected N^4-acetyl-cytidine **1b** (5 g, 7.42 mmol), imidazole (1.52 g, 22.26 mmol) and TBSCl (1.68 g, 11.13 mmol) followed by detritylation with 10% BSA solution in DCM/MeOH according to **general procedure A**. Compound **2b** was isolated as a white foam (2.52 g, 70%). ^{1}H-NMR (600 MHz, DMSO-d_6) δ 10.93 (s, 1H, NH), 8.41 (d, J = 7.5, 1H, H_6), 7.20 (d, J= 7.5, 1H, H_5), 5.84 (d, J= 2.5, 1H, $H_{1'}$), 5.45 (d, J= 6.3, 1H, H_a OCH_2O), 5.37 (d, J= 6.3, 1H, H_b OCH_2O), 5.25 (t, J= 4.5, 1H, 5′OH), 4.29-4.23 (m, 2H, $H_{2'}$, $H_{3'}$), 3.94-3.89 (m, 1H, $H_{4'}$), 3.82-3.75 (m, 1H, $H_{5'a}$), 3.59-3.52 (m, 1H, $H_{5'b}$), 2.35-2.22 (m, 2H, CH_2 PrOM), 2.10 (s, 3H, CH_3 Ac), 0.99 (t, J = 7.5, 3H, CH_3 PrOM), 0.84 (s, 9H, $OSiC(CH_3)_3$), 0.07 (2s, 6H, $OSi(CH_3)_2$). ^{13}C-NMR (151 MHz, DMSO-d_6) δ 173.0, 171.1, 162.5, 154.5, 145.1, 95.4, 88.5, 87.1, 84.2, 80.7, 68.7, 59.0, 26.7, 25.6, 24.3, 17.7, 8.6, -4.9, -5.2. HRMS-ESI $[M+H]^+$ calcd for $C_{21}H_{35}N_3O_8Si$ 486.2266, found 486.2257.

3′-*O*-*tert*-butyldimethylsilyl-2′-*O*-propionyloxymethyl-*N*^{6}-phenoxyacetyl-adenosine (2c)

2c was prepared from 5′-*O*-DMTr-2′-*O*-PrOM protected N^6-phenoxyacetyl adenosine **1c** (2.09 g, 2.65 mmol), imidazole (540 mg, 7.94 mmol) and TBSCl (598 mg, 3.97 mmol) followed by detritylation with 10% BSA solution in DCM/MeOH according to **general procedure A**. Compound **2c** was isolated as a white foam (1.21 g, 76%). ^{1}H-NMR (600 MHz, DMSO-d_6) δ 10.98 (s, 1H, NH), 8.76 (s, 1H, H_2), 8.69 (s, 1H, H_8), 7.34-7.27 (m, 2H, (o) phenyl), 7.02 - 6.92 (m, 3H, (m, p) phenyl), 6.13 (d, *J* = 6.2, 1H, $H_{1'}$), 5.30 (d, *J* = 6.4, 1H, OCH_2O), 5.24 (t, *J* = 5.5, 1H, 5′OH), 5.13 (d, *J* = 6.4, 1H, OCH_2O), 5.03 (bs, 2H, OCH_2OPh), 4.99-4.97 (m, 1H, $H_{2'}$), 4.57 (dd, *J* = 4.6, *J* = 3.9, 1H, $H_{3'}$), 3.99 (dd, *J* = 7.2, *J* = 4.1, 1H, $H_{4'}$), 3.75 (dt, *J* = 9.8, *J* = 4.9, 1H, $H_{5'a}$), 3.57 (dd, *J* = 11.0 , *J* = 5.7, 1H, $H_{5'b}$), 2.04-1.77 (m, 2H, CH_2 PrOM), 0.91 (s, 9H, $OSiC(CH_3)_3$), 0.76 (t, J = 7.5, 3H, CH_3 PrOM), 0.13 and 0.08 (2s, 6H, CH_3Si). ^{13}C-NMR (151 MHz, DMSO-d_6) δ 172.8, 167.4, 157.8, 151.8, 151.6, 149.2, 143.4, 129.5, 123.5, 121.2, 114.6, 87.8, 86.5, 85.8, 80.2, 71.0, 67.2, 60.8, 39.5, 26.4, 25.7, 17.9, 8.4, -5.0, -5.3. HRMS-ESI $[M+H]^+$ calcd for $C_{28}H_{40}N_5O_8Si$ 602.2641, found 602.2647.

3′-*O*-*tert*-butyldimethylsilyl-2′-*O*-propionyloxymethyl-N^2-*iso*propylphenoxyacetyl-guanosine (2d)

2d was prepared from 5′-*O*-DMTr-2′-*O*-PrOM protected N^2-*iso*propylphenoxyacetyl guanosine **1d** (5 g, 5.89 mmol), imidazole (1.2 g, 17.69 mmol) and TBSCl (1.34 g, 8.85 mmol) followed by detritylation with 10% BSA solution in DCM/MeOH according to **general procedure A**. Compound **2d** was isolated as a white foam (3.4 g, 88%). ^{1}H-NMR (600 MHz, DMSO-d_6) δ 11.82 (s, 1H, NH), 11.77 (s, 1H, NH *i*PrPac), 8.33 (s, 1H, H_8), 7.17 (d, *J* = 9.7, 2H, (o) Ph), 6.88 (d, *J* = 6.7, 2H, (m) Ph), 5.93 (d, *J* = 7.0, 1H, $H_{1'}$), 5.30 (d, *J* = 6.4, 1H, OCH_2O), 5.20 (t, *J* = 5.1, 1H, 5′OH), 5.13 (d, *J* = 6.4, 1H, OCH_2O), 4.83 (bs, 2H, $NHCOCH_2Ph$), 4.80-4.78 (m, 1H, $H_{2'}$), 4.47 (dd, *J* = 4.7, *J* = 1.9, 1H, $H_{3'}$), 3.97 (m, 1H, $H_{4'}$), 3.64 (dt, *J* = 10.3, *J* = 5.2, 1H , $H_{5'a}$), 3.61-3.55 (m, 1H, $H_{5'b}$), 2.87-2.78 (m, 1H, $C\mathit{H}(CH_3)_2$ *i*PrPac), 2.04-1.97 (m, 2H, CH_2, PrOM), 1.17 (d, *J* = 6.9, 6H, $CH(C\mathit{H}_3)_2$), 0.91 (s, 9H, $OSiC(C\mathit{H}_3)_3$), 0.84 (t, *J* = 7.5, 3H, CH_3 PrOM), 0.13 (2s, 6H, CH_3Si). ^{13}C-NMR (151 MHz, DMSO-d_6) δ = 172.9, 171.1, 155.7, 154.8, 148.8, 147.4, 141.3, 137.8, 127.2, 120.3, 114.4, 87.8, 86.9, 84.6, 80.8, 71.4, 66.4, 60.9, 32.6, 26.4, 25.6, 24.1, 17.9, 8.5, -4.1, -5.0. HRMS-ESI $[M+H]^+$ calcd for $C_{31}H_{46}N_5O_9Si$ 660.3059, found 660.3051.

<u>General procedure B for synthesis of compounds 3a-d and 6a-d with a 5′-photolabile group</u>

Compounds **2a-d** (1 eq) were dried over P_2O_5 overnight and the following day were dissolved in anhydrous DCM (0.1 M) and the solution was stirred at room temperature for 30 min over 3Å

molecular sieves. NPPOC-Cl or PhSNPPOC-Cl (2.5 eq) was added to a solution of 1-methylimidazole (7.5 eq) in anhydrous DCM and the solution was left stirring at 0°C for 30 min over 3Å molecular sieves. The nucleoside-containing solution was transferred dropwise to the equivolume solution of 1-methylimidazole and NPPOC-Cl or PhSNPPOC-Cl (final conc. of nucleoside 0.03 M) and the reaction mixture was stirred for 2.5 to 16 h at room temperature. The mixture was diluted with DCM, extracted with saturated solution of $NaHCO_3$. The organic layer was dried over Na_2SO_4, evaporated under reduced pressure and the crude was purified on high performance flash chromatography system on a SiO_2 column (0-100% gradient of ethyl acetate in cyclohexane). Fractions containing the pure products **3a-d** or **6a-d** were collected, evaporated under reduced pressure and evaporated several times with DCM and pentane to provide a white foam.

5′-*O*-2-(2-nitrophenyl)propyloxycarbonyl-3′-*O*-*tert*-butyldimethylsilyl-2′-*O*-propionyloxy-methyl uridine (3a)

3a was prepared from nucleoside **2a** (2 g, 4.5 mmol) and NPPOC-Cl (2.74 g, 11.27 mmol) in the presence of 1-methylimidazole (2.69 mL, 33.75 mmol) according to **general procedure B**. Compound **3a** was isolated as a white foam (2.61 g, 89%). ^{1}H-NMR (600 MHz, DMSO-d_6) δ 11.42 (s, 1H, NH), 7.83-7.81 (m, 1H, H_3 NPPOC), 7.72-7.67 (m, 2H, H_4 and H_5 NPPOC), 7.62 (t, J = 8.1, 1H, H_6), 7.52-7.46 (m, 1H, H_6 NPPOC), 5.82 (d, J = 5.1, 1H, $H_{1'}$), 5.57 (d, J = 8.0, 1H, H_5), 5.36 (d, J = 6.4, OCH_2O), 5.19 (m, 1H, OCH_2O), 4.40 (m, 1H, $H_{2'}$), 4.35-4.25 (m, 4H, $H_{3'}$,$H_{5'}$, $OCH_2CH(CH_3)PhNO_2$), 4.20-4.17 (m, 1H, $H_{5'}$), 3.98-3.97 (m, 1H, $H_{4'}$), 3.52-3.49 (m, 1H, $OCH_2CH(CH_3)PhNO_2$), 2.28-2.4 (m, 2H, CH_2 PrOM), 1.29 (d, J = 6.97, 3H, $OCH_2CH(CH_3)PhNO_2$), 0.97 (t, J =7.5, 3H, CH_3 PrOM) , 0.86 (2s, 9H, $OSiC(CH_3)_3$), 0.06 and 0.05 (2s, 3H, $SiC(CH_3)_2$), 0.04 and 0.03 (2s, 3H, $SiC(CH_3)_2$). ^{13}C-NMR (151 MHz, DMSO-d_6) δ 172.7, 163.0, 153.8, 150.4, 150.1, 150.0, 140.7, 136.1, 132.9, 128.5, 128.4, 127.9, 123.8, 123.7, 102.0, 87.7, 87.5, 87.4, 81.1, 81.0, 79.2, 71.3, 71.2, 70.1, 70.0, 66.2, 32.8, 32.7, 26.6, 25.5, 17.7, 17.6, 17.5, 8.4, -5.4, -4.9. HRMS-ESI $[M+Na]^+$ calcd for $C_{29}H_{41}N_3O_{12}SiNa$ 674.2357, found 674.2364.

5′-*O*-2-(2-nitrophenyl)propyloxycarbonyl-3′-*O*-*tert*-butyldimethylsilyl-2′-*O*-propionyloxy-methyl *N*^{4}-acetyl-cytidine (3b)

3b was prepared from nucleoside **2b** (2 g, 4.12 mmol) and NPPOC-Cl (2.51 g, 10.30 mmol) in the presence of 1-methylimidazole (2.95 mL, 37.03 mmol) according to **general procedure B**. Compound **3b** was isolated as a white foam (2.55 g, 89%). ^{1}H-NMR (600 MHz, DMSO-d_6) δ 10.96 (s, 1H, NH), 8.01 (dd, *J* = 7.5, 1.1, 1H, H_6), 7.83 (d, *J* = 8.1, 1H, H_5 NPPOC), 7.74-7.64 (m, 2H, H_6, H_3 NPPOC), 7.55-7.41 (m, 1H, H_4 NPPOC), 7.25-7.15 (m, 1H, H_5), 5.85-5.79 (m, 1H, $H_{1'}$), 5.45 (d, *J* = 6.3, 1H, H_a OCH_2O), 5.32 (dd, *J* = 6.3, 3.4, 1H, H_b OCH_2O), 4.38-4.17 (m, 6H, $H_{2'}$,$H_{3'}$,$H_{5'a,b}$, OCH_2CH(CH_3)$PhNO_2$), 4.09-3.99 (m, 1H, $H_{4'}$), 3.51 (m, *J* = 6.9, 1H, OCH_2C*H*(CH_3)$PhNO_2$), 2.33-2.22 (m, 2H, CH_2 PrOM), 2.10 (s, 3H, CH_3 Ac), 1.30 (d, *J* = 7.0, 3H, OCH_2CH(*CH_3*)$PhNO_2$), 0.99 (t, *J* = 7.5, 3H, CH_3 PrOM), 0.83 (s, 9H, OSiC(CH_3)$_3$), 0.04 and 0.02 (2s, 6H, OSi(CH_3)$_2$). ^{13}C-NMR (151 MHz, DMSO-d_6) δ 173.0, 171.1, 162.6, 154.4, 154.0, 150.1, 145.1, 136.1, 133.0, 128.5, 128.4, 128.0, 123.8, 95.7, 89.5, 87.2, 80.6, 79.8, 71.3, 71.3, 69.5, 66.1, 32.8, 26.7, 25.5, 24.3, 17.6, 17.4, 8.5, -5.0, -5.5. HRMS-ESI $[M+H]^+$ calcd for $C_{31}H_{45}N_4O_{12}Si$ 693.2803, found 693.2806.

5′-*O*-2-(2-nitrophenyl)propyloxycarbonyl-3′-*O*-*tert*-butyldimethylsilyl-2′-*O*-propionyloxy-methyl-*N*^{6}-phenoxyacetyl-adenosine (3c)

3c was prepared from nucleoside **2c** (1.18 g, 1.96 mmol) and NPPOC-Cl (1.20 g, 4.91 mmol) in the presence of 1-methylimidazole (1.17 mL, 14.73 mmol) according to **general procedure B**. Compound **3c** was isolated as a white foam (1.47 g, 92%). ^{1}H-NMR (600 MHz, DMSO-d_6) δ 11.03 (s, 1H, NH), 8.70 (2s, 1H, H_2), 8.68 (2s, 1H, H_8), 7.82 (d, *J* = 8.0, 1H, H_3 NPPOC), 7.76-7.61 (m, 2H, H_4, H_5 NPPOC), 7.49-7.45 (m, 1H, H_6 NPPOC), 7.32-7.28 (m, 2H, (o) Ph), 6.98 -6.94 (m, 3H, (m)(p) Ph), 6.14 (d, *J* = 5.7, 1H, $H_{1'}$), 5.33 (2d, *J* = 6.4, 1.9, 1H, OCH_2O), 5.13-5.08 (m, 2H, OCH_2O, $H_{2'}$), 5.04 (bs, 2H, NH, $NHCOCH_2Ph$), 4.67-4.64 (m, 1H, $H_{3'}$), 44.41-4.37 (m, 1H, $H_{5'a}$), 4.38-4.23 (m, 3H, $H_{5'b}$+ OCH_2CH(CH_3)$PhNO_2$), 4.13-4.11 (m, $H_{4'}$), 3.49-3.45 (m, 1H, OCH_2C*H*(CH_3)$PhNO_2$), 1.98-1.78 (m, 2H, CH_2 PrOM), 1.26 (d, *J* = 6.9, 3H, OCH_2CH(*CH_3*)$PhNO_2$), 0.89 (s, 9H, SiC(CH_3)$_3$), 0.75 (m, 3H, CH_3 PrOM), 0.10 and 0.09 (2s, 6H, $SiCH_3$). ^{13}C-NMR (151 MHz, DMSO-d_6) δ 172.8, 167.4, 157.8, 154.0, 151.8, 151.5, 150.1, 149.2, 143.7, 136.2, 133.0, 129.5, 128.5, 128.0, 123.8, 123.6, 121.1, 114.5, 87.8, 86.1, 82.3, 82.2, 79.2, 71.2, 71.2, 70.8, 70.7, 67.2, 66.59, 66.53, 32.5, 26.4, 25.6, 17.8, 17.6, 8.3, -4.8, -5.3. HRMS-ESI $[M+H]^+$ calcd for $C_{38}H_{49}N_6O_{12}Si$ 809.3178, found 809.3172.

5′-*O*-2-(2-nitrophenyl)propyloxycarbonyl-3′-*O*-*tert*-butyldimethylsilyl-2′-*O*-propionyloxy-methyl-*N*^{2}-*iso*propylphenoxyacetyl-guanosine (3d)

3d was prepared from nucleoside **2d** (2 g, 3.03 mmol) and NPPOC-Cl (1.85 g, 7.58 mmol) in the presence of 1-methylimidazole (1.82 mL, 22.73 mmol) according to **general procedure B**. Compound **3d** was isolated as a white foam (2.16 g, 82%). ^{1}H-NMR (600 MHz, DMSO-d_6) δ 11.85 (s, 1H, NH, NH), 11.69 (s, 1H, NH *i*PrPac), 8.26 (d, 1H, J = 8.9, H_8), 7.83 (s, 1H, H_3 NPPOC), 7.73 -7.60 (m, 2H, H_4, H_5 NPPOC), 7.51-7.46 (m, 1H, H_6 NPPOC), 7.16 (d, 2H, *J* = 8.6, (o) Ph), 6.89 (d, 2H, *J* = 8.6, (m) Ph), 5.91 (d, *J* = 6.8, 1H, $H_{1'}$), 5.31 (d, *J* = 6.3, 1H, OCH_2O), 5.10 (dd, *J* = 8.5, 6.5, 1H, OCH_2O), 4.87-4.82 (m, 3H, $H_{2'}$, $NHCOCH_2Ph$), 4.46-4.45 (m, 1H, $H_{3'}$), 4.33-4.23 (m, 4H, $OC\mathit{H_2}CH(CH_3)PhNO_2$ + $H_{5'a,b}$), 4.10-4.07 (m, 1H, $H_{4'}$), 3.52-3.47 (m, 1H, $OCH_2C\mathit{H}(CH_3)PhNO_2$), 2.86-2.80 (m, 1H, $C\mathit{H}(CH_3)_2$ *i*PrPac), 2.07-1.94 (m, 2H, CH_2 PrOM), 1.28 (2d, *J* = 2.76, 3H, $OCH_2CH(C\mathit{H_3})PhNO_2$), 1.17, 1.15 (2s, 6H, $CH(C\mathit{H_3})_2$), 0.88 (m, 9H, $OSiCH(C\mathit{H_3})_3$), 0.12 – 0.07 (m, 6H, CH_3Si).). ^{13}C-NMR (150 MHz, DMSO-d_6) δ 172.8, 171.1, 155.7, 154.9, 154.1, 150.1, 148.8, 147.5, 141.4, 138.0, 136.2, 133.1, 128.5, 128.0, 127.3, 123.9, 120.5, 114.4, 87.8, 84.6, 82.7, 79.7, 71.4, 71.3, 71.1, 71.0, 66.6, 66.5, 32.8, 32.7, 32.6, 26.5, 25.6, 24.1, 17.9, 17.6, 8.5, -4.9, -5.3. HRMS-ESI $[M+H]^+$ calcd for $C_{41}H_{55}N_6O_{13}Si$ 867.3596, found 867.3591.

General procedure C for 3′-desilylation and synthesis of compounds 4a-d and 7a-d

To a solution of fully protected nucleosides **3a-d** or **6a-d** (1 eq) in THF (0.1 M), triethylamine trihydrofluoride (6 eq) was added and the reaction was left stirring at room temperature for 24 to 48 h. The reaction was neutralized with TEAAc buffer pH 7 (2 M), diluted with H_2O, extracted two times with EtOAc and washed with brine. The organic layer was dried over Na_2SO_4, concentrated under reduced pressure and purified on high performance flash chromatography system on a SiO_2 column (0-10% gradient of MeOH in DCM). Fractions containing the pure products **4a-d** and **7a-d** were evaporated and co-evaporated several times with DCM and pentane to yield a white foam.

5′-*O*-2-(2-nitrophenyl)propyloxycarbonyl-2′-*O*-propionyloxymethyl uridine (4a)

4a was prepared from nucleoside **3a** (2.5 g, 3.83 mmol) according to **general procedure C**. Compound **4a** was isolated as a white foam (1.77 g, 86%). ^{1}H-NMR (600 MHz, DMSO-d_6) δ 11.42 (s, 1H, NH), 7.83 (d, *J* = 8.3, 1H, H_5 NPPOC), 7.73-7.66 (m, 2H, H_5, H_6 NPPOC), 7.57 (dd, *J* = 8.1, 6.2, 1H, H_6), 7.52-7.46 (m, 1H, H_4 NPPOC), 5.80 (d, *J* = 4.7, 1H, $H_{1'}$), 5.56 (d, *J* = 8.1, 1H,

H_5), 5.51 (d, $J = 4.7$, 1H, 3′OH), 5.35 (d, $J = 6.5$, 1H, H_a OCH_2O), 5.20 (d, $J = 6.5$, 1H, H_b OCH_2O), 4.36-4.24 (m, 4H, $H_{2'}$, $H_{5'a}$, $OCH_2CH(CH_3)PhNO_2$), 4.21 (ddd, $J = 11.8, 5.9, 1.6$, 1H, $H_{5'b}$), 4.06 (d, $J = 3.3$, 1H, $H_{3'}$), 3.97 (dt, $J = 8.9, 4.5$, 1H, $H_{4'}$), 3.57-3.45 (m, 1H, $OCH_2CH(CH_3)PhNO_2$), 2.33-2.22 (m, 2H, CH_2 PrOM), 1.27 (t, $J = 12.0$, 3H, $OCH_2CH(CH_3)PhNO_2$), 0.99 (t, $J = 7.5$, 3H, CH_3 PrOM). ^{13}C-NMR (151 MHz, DMSO-d_6) δ 173.1, 162.9, 154.0, 150.4, 150.1, 140.5, 136.1, 133.0, 128.5, 128.0, 123. 8, 102.0, 87.8, 87.4, 81.0, 79.8, 71.2, 68.6, 67.0, 32.80, 26.70, 17.6, 8.6. HRMS-ESI $[M+H]^+$ calcd for $C_{23}H_{27}N_3O_{12}$ 538.1667, found 538.1659.

5′-*O*-2-(2-nitrophenyl)propyloxycarbonyl-2′-*O*-propionyloxymethyl-*N*^{4}-Acetyl-cytidine (4b)

4b was prepared from nucleoside **3b** (2.5 g, 3.61 mmol) according to **general procedure C**. Compound **4b** was isolated as a white foam (1.77 g, 85%). ^{1}H-NMR (600 MHz, DMSO-d_6) δ 10.94 (s, 1H, NH), 8.04-7.93 (m, 1H, H_6), 7.83 (d, $J = 7.8$, 1H, H_3 NPPOC), 7.76-7.65 (m, 2H, H_5, H_6 NPPOC), 7.52-7.43 (m, 1H, H_4 NPPOC), 7.23-7.13 (m, 1H, H_5), 5.80 (t, $J = 3.1$, 1H, $H_{1'}$), 5.49 (s, 1H, 3′OH), 5.39 (d, $J = 6.4$, 1H, H_a OCH_2O), 5.30 (dd, $J = 6.4, 2.5$, 1H, H_b OCH_2O), 4.41-4.21 (m, 5H, $H_{4'}$, $H_{5'a,b}$, $OCH_2CH(CH_3)PhNO_2$), 4.02 (s, 2H, $H_{2'}$, $H_{3'}$), 3.56-3.46 (m, 1H, $OCH_2CH(CH_3)PhNO_2$), 2.29 (q, $J = 7.5$, 2H, CH_2 PrOM), 2.11 (s, 3H, CH_3 Ac), 1.29 (d, $J = 7.0$, 3H, $OCH_2CH(CH_3)PhNO_2$), 0.99 (t, $J = 7.5$, 3H, CH_3 PrOM). ^{13}C-NMR (151 MHz, DMSO-d_6) δ 173.2, 171.1, 162.6, 154.3, 154.1, 150.1, 145.1, 136.1, 133.0, 128.5, 128.0, 123.8, 95.6, 89.6, 87.5, 81.9, 80.6, 71.3, 68.4, 66.8, 32.8, 26.8, 24.4, 17.5, 8.6. HRMS-ESI $[M+H]^+$ calcd for $C_{25}H_{31}N_4O_{12}$ 579.1938, found 579.1944.

5′-*O*-2-(2-nitrophenyl)propyloxycarbonyl-2′-*O*-propionyloxymethyl-*N*^{6}-phenoxyacetyl-adenosine (4c)

4c was prepared from nucleoside **3c** (1.47 g, 1.82 mmol) according to **general procedure C**. Compound **4c** was isolated as a white foam (1.04 g, 82%).^{1}H-NMR (600 MHz, DMSO-d_6) δ 11.01 (s, 1H, NH), 8.68 (2s, 1H, H_2), 8.66 (2s, 1H, H_8), 7.82 (d, $J = 8.0$, 1H, H_3 NPPOC), 7.76-7.61 (m, 2H, H_4, H_5 NPPOC), 7.49-7.45 (m, 1H, H_6 NPPOC), 7.32-7.28 (m, 2H, (o) Ph), 6.98-6.94 (m, 3H, (m), (p) Ph), 6.13 (d, $J = 5.7$, 1H, $H_{1'}$), 5.70 (dd, $J = 5.54, 2.04$, 1H, 3′OH), 5.34-5.32 (m, 1H, OCH_2O), 5.20-5.18 (m, 1H, OCH2O), 5.04 (bs, 2H, NH, $NHCOCH_2Ph$), 6.95 (t, $J = 5.13$, 1H, $H_{2'}$), 4.47-4.45 (m, 1H, $H_{3'}$), 4.38-4.36 (m, 1H, $H_{5'a}$), 4.29-4.23 (m, 3H, $H_{5'b}$, $OCH_2CH(CH_3)PhNO_2$), 4.13-4.10 (m, $H_{4'}$), 3.49-3.45 (m, 1H, $OCH_2CH(CH_3)PhNO_2$), 2.07-1.94 (m, 2H, CH_2 PrOM), 1.26 and 1.24 (2d, $J = 1.32$, 3H, $OCH_2CH(CH_3)PhNO_2$), 0.89-0.80 (m, 3H, CH_3 PrOM). ^{13}C-NMR (151

MHz, DMSO-d_6) δ 173.0, 167.5, 157.8, 154.1, 151.9, 151.5, 150.0, 149.2, 143.3, 136.2, 133.1, 129.6, 128.5, 128.0, 123.9, 123.4, 121.1, 114.6, 87.9, 86.3, 81.9, 80.0, 71.2, 69.1, 67.3, 67.2, 32.8, 26.6, 17.61, 8.5. HRMS-ESI $[M+H]^+$ calcd for $C_{32}H_{36}N_6O_{12}$ 695.2307, found 695.2312.

5′-*O*-2-(2-nitrophenyl)propyloxycarbonyl-2′-*O*-propionyloxymethyl-*N*^{2}-*iso*propylphenoxy-acetyl-guanosine (4d)

4d was prepared from nucleoside **3d** (2.16 g, 2.49 mmol) according to **general procedure C**. Compound **4d** was isolated as a white foam (1.5 g, 83%).^{1}H-NMR (600 MHz, DMSO-d_6) δ 11.82 and 11.72 (2s, 2H, NH and NH *i*PrPac), 8.19 and 8.18 (2s, 1H, H_8), 7.83 (d, *J* = 8.0, 1H, H_3 NPPOC), 7.71-7.60 (m, 2H, H_4, H_5 NPPOC), 7.51-7.46 (m, 1H, H_6 NPPOC), 7.20-7.10 (d, *J* = 8.6, 2H, (o) Ph), 6.89 (d, *J* = 8.6, 2H, (m) Ph), 5.94 (dd, *J* = 5.8, 1.4, 1H, $H_{1'}$), 5.31 (dd, *J* = 6.5, 2.1, 1H, OCH_2O), 5.14 (dd, *J* = 6.5, 4.0 , 1H, OCH_2O), 4.83 (bs, 2H, $NHCOCH_2Ph$), 4.75-4.73 (m, 1H, $H_{2'}$), 4.37-4.33 (m, 1H, $H_{3'}$), 4.32-4.23 (m, 4H, $C\mathit{H_2}CH(CH_3)PhNO_2$, $H_{5'a,b}$), 4.14-4.08 (m, 1H, $H_{4'}$), 3.52-3.49 (m, 1H, $OCH_2C\mathit{H}(CH_3)PhNO_2$), 2.85-2.80 (m, 1H, $C\mathit{H}(CH_3)_2$ *i*PrPac), 2.16-2.09 (m, 2H, CH_2 PrOM), 1.28 and 1.27 (2d, 3H, $OCH_2CH(C\mathit{H_3})PhNO_2$), 1.17 (d, *J* = 6.9, 6H, $CH(C\mathit{H_3})_2$), 0.90-0.74 (m, 3H, CH_3 PrOM). ^{13}C-NMR (151 MHz, DMSO-d_6) δ 173.0, 171.1, 155.7, 154.9, 154.1, 150.1, 148.5, 147.4, 141.4, 138.0, 136.1, 133.0, 128.5, 128.0, 127.3, 123.7, 120.5, 114.2, 87.8, 84.4, 81.9, 80.5, 80.4, 71.2, 69.2, 67.4, 66.5, 32.8, 32.6, 26.6, 24.1, 17.6, 8.5. HRMS-ESI $[M+H]^+$ calcd for $C_{35}H_{41}N_6O_{13}$ 753.2726, found 753.2719.

General procedure D for synthesis of ribonucleoside 3′-*O*-phosphoramidites 5a-d and 8a-d

5′-*O*-NPPOC ribonucleosides **4a-d** or **7a-d** (1 eq) were dried over P_2O_5 overnight prior to dissolution in anhydrous DCM (0.12 M). This solution was left stirring over 3Å molecular sieves and under argon for 30 min at room temperature. In a separate flask, anhydrous DCM was stirred under argon over 3Å molecular sieves, and 2-cyanoethyl *N,N*-di*iso*propylchlorophosphoramidite (2.5 eq) was added, followed by the addition of *N,N*-di*iso*propylethylamine (2.8 eq). This solution was transferred dropwise to the nucleoside-containing solution (final conc. 0.1 M) and the reaction mixture was stirred for 2.5 h at room temperature. The reaction mixture was neutralized with 1:1 mixture of saturated $NaHCO_3$ and NaCl solutions and extracted three times with DCM. The organic layer was dried over Na_2SO_4, concentrated under reduced pressure and purified on high performance flash chromatography system on a SiO_2 column with a 0 to 50% gradient of acetone in DCM containing 1% Pyridine. Fractions with the pure products **5a-d** or **8a-d** were pooled,

evaporated under reduced pressure, co-evaporated with toluene, precipitated in hexane. The solid was dissolved in DCM and the solution was evaporated to yield a white foam.

5′-*O*-2-(2-nitrophenyl)propyloxycarbonyl-2′-*O*-propionyloxymethyl-3′-*O*-(2-cyanoethyl)-(*N,N*-di*iso*propyl)-phosphoramidite uridine (5a)

5a was prepared from nucleoside **4a** (780 mg, 1.45 mmol) in reaction with *N,N*-di*iso*propylchlorophosphoramidite (810 µL, 3.63 mmol) and di*iso*propylethylamine (707 µL, 4.06 mmol) according to **general procedure D**. Compound **5a** was isolated as a white foam (1 g, 95%). ^{31}P-NMR (162 MHz, $CDCl_3$) δ 150.78, 150.70, 150.46, 150.39. HRMS-ESI $[M+H]^+$ calcd for $C_{32}H_{45}N_5O_{13}P$ 738.2746, found 738.2743.

5′-*O*-2-(2-nitrophenyl)propyloxycarbonyl-2′-*O*-propionyloxymethyl-3′-*O*-(2-cyanoethyl)-(*N,N*-di*iso*propyl)-phosphoramidite-*N*^{4}-acetyl-cytidine (5b)

5b was prepared from nucleoside **4b** (1.27 g, 2.20 mmol) in reaction with *N,N*-di*iso*propylchlorophosphoramidite (1.23 mL, 5.49 mmol) and di*iso*propylethylamine (1.07 mL, 6.15 mmol) according to **general procedure D**. Compound **5b** was isolated as a white foam (1.27 g, 74 %). ^{31}P-NMR (162 MHz, $CDCl_3$) δ 150.06. HRMS-ESI $[M+H]^+$ calcd for $C_{34}H_{48}N_6O_{13}P$ 779.3022, found 779.3011.

5′-*O*-2-(2-nitrophenyl)propyloxycarbonyl-2′-*O*-propionyloxymethyl-3′-*O*-(2-cyanoethyl)-(*N,N*-di*iso*propyl)-phosphoramidite-*N*^{6}-phenoxyacetyl-adenosine (5c)

5c was prepared from nucleoside **4c** (500 mg, 0.72 mmol) in reaction with *N,N*-di*iso*propylchlorophosphoramidite (402 µL, 1.80 mmol) and di*iso*propylethylamine (351 µL, 2.02 mmol) according to **general procedure D**. Compound **5c** was isolated as a white foam (489 mg, 75 %). ^{31}P-NMR (162 MHz, $CDCl_3$) δ 151.00, 150.97, 150.84, 150.81. HRMS-ESI $[M+H]^+$ calcd for $C_{41}H_{52}N_8O_{13}P$ 895.3386, found 895.3394.

5′-*O*-2-(2-nitrophenyl)propyloxycarbonyl-2′-*O*-propionyloxymethyl-3′-*O*-(2-cyanoethyl)-(*N,N*-di*iso*propyl)-phosphoramidite-*N*^{2}-*iso*propylphenoxyacetyl-guanosine (5d)

5d was prepared from nucleoside **4d** (340 mg, 0.45 mmol) in reaction with *N,N*-di*iso*propylchlorophosphoramidite (252 µL, 1.13 mmol) and di*iso*propylethylamine (220 µL, 1.26 mmol) according to **general procedure D**. Compound **5d** was isolated as a white foam (413 mg, 95 %). ^{31}P-NMR (162 MHz, $CDCl_3$) δ 151.16, 151.14, 150.81. HRMS-ESI $[M+H]^+$ calcd for $C_{44}H_{58}N_8O_{14}P$ 953.3810, found 953.3822.

5′-*O*-2-(2-nitro-4-ethyl-5-thiophenyl-phenyl)propyloxycarbonyl-3′-*O*-*tert*-butyldimethyl-silyl-2′-*O*-propionyloxymethyl uridine (6a)

6a was prepared from nucleoside **2a** (750 mg, 1.69 mmol) and SPhNPPOC-Cl (1.60 g, 4.22 mmol) in the presence of 1-methylimodazole (1.01 mL, 12.65 mmol) according to **general procedure B**. Compound **6a** was isolated as a white foam (1.11 g, 84%). ^{1}H-NMR (600 MHz, DMSO-d_6) δ 11.4 (s, 1H, NH), 7.81 (d, J = 1.8, 1H, H_5 NPPOC), 7.62 (dd, J = 8.0, 6.7, 1H, H_6), 7.50-7.38 (m, 5H, SPhNPPOC), 7.09 (d, J = 4.2, 1H, H_2 NPPOC), 5.80 (dd, J = 4.7, 1.4, 1H, $H_{1'}$), 5.56 (dt, J = 13.1, 6.5, 1H, H_5), 5.37 (d, J = 6.4, 1H, OCH_2O), 5.17 (dd, J = 6.4, 3.1, 1H, OCH_2O), 4.39 (td, J = 5.1, 2.4, 1H, $H_{2'}$), 4.31-4.23 (m, 2H, $H_{5'}$, $H_{3'}$), 4.22-4.10 (m, 2H, $OC\mathit{H_2}CH(CH_3)PhNO_2$, $H_{5'}$), 4.06-3.95 (m, 2H, $OC\mathit{H_2}CH(CH_3)PhNO_2$, $H_{4'}$), 3.53 (dd, J = 12.5, 5.5, 1H, $OCH_2C\mathit{H}(CH_3)PhNO_2$), 2.75 (q, J = 7.5, 2H, CH_2 (SPhNPPOC)), 2.26 (qd, J = 7.5, 2.2, 2H, CH_2 PrOM), 1.20 (t, J = 7.5, 3H, CH_3 (SPhNPPOC)), 1.06 (d, J = 6.9, 3H, $OCH_2CH(C\mathit{H_3})PhNO_2$), 0.98 (td, J = 7.5, 1.1, 3H, CH_3 PrOM), 0.84 (2s, 9H, $OSiC(CH_3)_3$), 0.04 (2s, 6H, $OSi(CH_3)_2$). ^{13}C-NMR (151 MHz, DMSO-d_6) δ 172.9, 162.9, 153.9, 150.4, 148.0, 147.9, 142.1, 140.8, 134.7, 132.4, 132.3, 131.8, 130.0, 128.7, 128.5, 124.1, 102.0, 87.8, 87.5, 81.0, 79.2, 71.0, 70.1, 66.3, 32.4, 26.7, 25.6, 25.5, 17.7, 17.1 17.0, 13.6, 8.6, -4.9, -5.4. HRMS-ESI $[M+H]^+$ calcd for $C_{37}H_{50}N_3O_{12}Si$ 788.2879, found 788.2884.

5′-*O*-2-(2-nitro-4-ethyl-5-thiophenyl-phenyl)propyloxycarbonyl-3′-*O*-*tert*-butyldimethyl-silyl-2′-*O*-propionyloxymethyl *N*^{4}-acetyl-cytidine (6b)

6b was prepared from nucleoside **2b** (1.3 g, 2.68 mmol) and SPhNPPOC-Cl (2.54 g, 6.69 mmol) in the presence of 1-methylimodazole (1.6 mL, 20.08 mmol) according to **general procedure B**. Compound **6b** was isolated as a white foam (2.11 g, 95%).^{1}H-NMR (600 MHz, DMSO-d_6) δ 10.95 (s, 1H, NH), 8.02 (dd, J = 7.5, 0.9, 1H, H_6), 7.81 (d, J = 2.7, 1H, H_5 NPPOC), 7.47-7.39 (m, 5H, SPhNPPOC), 7.20 (dd, J = 7.5, 1.1, 1H, H_5), 7.09 (d, J = 4.0, 1H, H_2 NPPOC), 5.83 (t, J = 3.1, 1H, $H_{1'}$), 5.46-5.44 (m, 1H, H_a OCH_2O), 5.32 (dd, J = 6.3, 3.8, 1H, H_b OCH_2O), 4.37-4.31 (m, 2H, $H_{2'}$, $H_{5'}$), 4.3 (ddd, J = 12.0, 5.7, 2.5, 1H, $H_{5'}$), 4.2 (dt, J = 6.8, 4.8, 1H, $H_{3'}$), 4.14 (td, J = 10.4, 5.8, 1H, $OC\mathit{H_2}CH(CH_3)PhNO_2$), 4.08-4.01 (m, 2H, $OC\mathit{H_2}CH(CH_3)PhNO_2$, $H_{4'}$), 3.57-3.51 (m, 1H, $OCH_2C\mathit{H}(CH_3)PhNO_2$), 2.74 (q, J = 7.5, 2H, CH_2 (SPhNPPOC)), 2.30-2.23 (m, 2H, CH_2 PrOM), 2.10 (d, J = 2.9, 3H, CH_3 Ac), 1.21-1.17 (m, 3H, CH_3 (SPhNPPOC)), 1.06 (d, J = 6.9, 3H, $OCH_2CH(C\mathit{H_3})PhNO_2$), 0.98 (td, J = 7.5, 1.4, 3H, CH_3 PrOM), 0.8 (t, J = 3.5, 9H, $OSiC(CH_3)_3$), 0.02 (dd, J = 17.2, 6.4 6H, $OSi(CH_3)_2$). ^{13}C-NMR (151 MHz, DMSO-d_6) δ 173.0, 171.0, 162.6, 154.4, 153.9, 148.0, 147.9, 145.3, 142.2, 141.5, 134.6, 132.4, 131.8, 130.0, 128.7 and 128.6, 128.5,

124.1, 95.7, 89.7, 87.3, 80.7, 80.6, 79.8, 71.1, 70.9, 69.7, 69.6, 66.3, 66.2, 59.8, 32.4, 26.7, 25.6, 25.5, 24.4, 17.6, 17.1, 13.7, 8.5, -4.9, -5.5. HRMS-ESI $[M+H]^+$ calcd for $C_{39}H_{53}N_4O_{12}SSi$ 829.3131, found 829.3146.

5′-*O*-2-(2-nitro-4-ethyl-5-thiophenyl-phenyl)propyloxycarbonyl-3′-*O*-*tert*-butyldimethyl-silyl-2′-*O*-propionyloxymethyl-*N*^{6}-phenoxyacetyl-adenosine (6c)

6c was prepared from nucleoside **2c** (2.30 g, 3.82 mmol) and SPhNPPOC-Cl (3.63 g, 9.56 mmol) in the presence of 1-methylimodazole (2.29 mL, 28.67 mmol) according to **general procedure B**. Compound **6c** was isolated as a white foam (3.10 g, 86%). ^{1}H-NMR (600 MHz, DMSO-d_6) δ 10.98 (s, 1H, NH), 8.70 (d, J = 1.6, 1H, H_2), 8.66 (d, J = 7.2, 1H, H_8), 7.80 (d, J = 3.5, 1H, H_5 NPPOC), 7.44-7.37 (m, 5H, SPhNPPOC), 7.32-7.28 (m, 2H, (o) Pac), 7.06 (s, 1H, H_2 NPPOC), 6.98-6.95 (m, 3H, (m) + (p) Pac), 6.15 (d, J = 5.8, 1H, $H_{1'}$), 5.33 (dd, J = 6.4, 3.1, 1H, OCH_2O), 5.13-5.08 (m, 2H, OCH_2O, $H_{2'}$), 5.03 (s, 2H, NHCOCH_2Ph), 4.65 (dd, J = 8.4, 4.7, 1H, $H_{3'}$), 4.37 (dt, J = 11.4, 5.6, 1H, $H_{5'}$), 4.32-4.26 (m, 1H, $H_{5'}$), 4.15-4.08 (m, 2H, OCH_2CH(CH_3)PhNO_2, $H_{4'}$), 4.02-3.96 (m, 1H, OCH_2CH(CH_3)PhNO_2), 3.53-3.47 (m, 1H, OCH_2C*H*(CH_3)PhNO_2), 2.73 (qd, J = 7.4, 2.1, 2H, CH_2 (SPhNPPOC)), 1.95-1.83 (m, 2H, CH_2 PrOM), 1.20-1.16 (m, 3H, CH_3 (SPhNPPOC)), 1.03 (d, J = 6.9, 3H, OCH_2CH(CH_3)PhNO_2), 0.89 (d, J = 2.8, 9H, $SiC(CH_3)_3$), 0.76 (td, J = 7.5, 4.0, 3H, CH_3 PrOM), 0.10-0.07 (m, 6H, $SiCH_3$). ^{13}C-NMR (151 MHz, DMSO-d_6) δ 172.7, 167.3, 157.8, 153.9, 151.7, 151.4, 149.2, 147.9, 147.8, 143.7, 142.0, 141.5, 134.6, 132.4, 131.7, 129.9, 129.5, 128.7, 128.5, 124.1, 123.6, 121.1, 114.5, 87.8, 86.1, 82.3, 82.2, 79.1, 70.8, 70.9, 67.2, 66.7, 32.4, 32.3, 26.3, 25.6, 17.8, 17.1, 13.7, 8.3, -4.9, -5.3. HRMS-ESI $[M+H]^+$ calcd for $C_{46}H_{57}N_6O_{12}SSi$ 945.3519, found 945.3517.

5′-*O*-2-(2-nitro-4-ethyl-5-thiophenyl-phenyl)propyloxycarbonyl-3′-*O*-*tert*-butyldimethyl-silyl-2′-*O*-propionyloxymethyl-*N*^{2}-*iso*propylphenoxyacetyl-guanosine (6d)

6d was prepared from nucleoside **2d** (1 g, 1.52 mmol) and SPhNPPOC-Cl (1.44 g, 3.79 mmol) in the presence of 1-methylimodazole (0.91 mL, 11.37 mmol) according to **general procedure B**. Compound **6d** was isolated as a white foam (1.28 g, 84%). ^{1}H-NMR (600 MHz, DMSO-d_6) δ 11.83 (s, 1H, NH), 11.64 (s, 1H, NH *i*PrPac), 8.27 (d, J = 5.9, 1H, H_8), 7.81 (s, 1H, H_5 NPPOC), 7.44-7.38 (m, 5H, SPhNPPOC), 7.16 (d, J = 8.5, 2H, (m) *i*PrPac), 7.08 (s, 1H, H_2 NPPOC), 6.91-6.87

(m, 2H, (o) *i*PrPac), 5.92 (d, *J* = 6.7, 1H, $H_{1'}$), 5.31 (d, *J* = 6.4, 1H, OCH_2O), 5.11 (dd, *J* = 6.3, 4.2, 1H, OCH_2O), 4.88-4.84 (m, 1H, $H_{2'}$), 4.81 (s, 2H, NHCOC*H₂*Ph), 4.46 (dd, *J* = 4.8, 2.6, 1H, $H_{3'}$), 4.36-4.23 (m, 2H, $H_{5'a,b}$), 4.16- 4.07 (m, 2H, $OCH_2CH(CH_3)PhNO_2$), 4.06-4.02 (m, 1H, $H_{4'}$), 3.57-3.49 (m, 1H, $OCH_2CH(CH_3)PhNO_2$), 2.87-2.79 (m, 1H, $CH(CH_3)_2$ *i*PrPac), 2.78-2.71 (m, 2H, CH_2 (SPhNPPOC)), 2.11-2.00 (m, 2H, CH_2 PrOM), 1.20 (t, *J* = 4.4, 3H, CH_3 (SPhNPPOC)), 1.16 (d, *J* = 7.0, 6H, $CH(CH_3)_2$ *i*PrPac), 1.05 (d, *J* = 6.9, 3H, $OCH_2CH(CH_3)PhNO_2$), 0.87 (d, *J* = 1.7, 9H, $OSiCH(CH_3)_3$), 0.85-0.81 (m, 3H, CH_3 PrOM), 0.08 (t, *J* = 3.0, 6H, CH_3Si). ^{13}C-NMR (150 MHz, DMSO-d_6) δ 172.8, 171.1, 155.7, 154.9, 153.9, 148.8, 147.9, 142.1, 141.6, 141.3, 138.1, 134.6, 132.5, 131.7, 130.0, 128.7, 128.5, 127.2, 124.1, 120.5, 114.4, 87.8, 84.6, 82.6, 79.7, 71.0, 66.7, 66.5, 32.6, 32.4, 26.4, 25.6, 24.1, 17.8, 17.1, 13.7, 8.4, -4.9, -5.3. HRMS-ESI $[M+H]^+$ calcd for $C_{49}H_{63}N_6O_{13}SSi$ 1003.3937, found 1003.3950.

5′-*O*-2-(2-nitro-4-ethyl-5-thiophenyl-phenyl)propyloxycarbonyl-2′-*O*-propionyloxymethyl uridine (7a)

7a was prepared from nucleoside **6a** (1.32 g, 1.68 mmol) according to **general procedure C**. Compound **7a** was isolated as a white foam (1.09 g, 96%). ^{1}H-NMR (600 MHz, DMSO-d_6) δ 11.42 (s, 1H, NH), 7.81 (s, 1H, H_5 NPPOC), 7.58 (dd, *J* = 10.9, 8.1, 1H, H_6), 7.50-7.39 (m, 5H, SPhNPPOC), 7.07 (s, 1H, H_2 NPPOC), 5.82 (dd, *J* = 4.7, 3.0, 1H, $H_{1'}$), 5.6 (d, *J* = 8.1, 1H, H_5), 5.5 (d, *J* = 8.1, 1H, 5′OH), 5.35 (d, *J* = 6.5, 1H, H_a OCH_2O), 5.20 (d, *J* = 6.5, 1H, H_b OCH_2O), 4.30 (dt, *J* = 7.6, 3.7, 2H, $H_{2'}$, $H_{5'}$), 4.24-4.17 (m, 1H, $H_{5'}$), 4.16-4.10 (m, 1H, $OCH_2CH(CH_3)PhNO_2$), 4.09 -3.94 (m, 3H, $OCH_2CH(CH_3)PhNO_2$, $H_{4'}$, $H_{3'}$), 3.58-3.47 (m, 1H, $OCH_2CH(CH_3)PhNO_2$), 2.75 (q, *J* = 7.5, 2H, CH_2(SPhNPPOC)), 2.34-2.22 (m, 2H, CH_2 PrOM), 1.20 (t, *J* = 7.5, 3H, CH_3(SPhNPPOC)), 1.05 (d, *J* = 6.8, 3H, $OCH_2CH(CH_3)PhNO_2$), 0.98 (t, *J* = 7.5, 3H, CH_3 PrOM). ^{13}C-NMR (151 MHz, DMSO-d_6) δ 173.1, 162.9, 153.9, 150.4, 147.8, 142.0, 141.7, 140.7, 140.5, 134.6, 132.6, 131.6, 130.0, 128.8, 128.3, 124.1, 102.0, 87.8, 87.5, 87.3, 80.9, 79.8, 70.8, 68.6, 67.1, 67.0, 32.4, 26.7, 25.6, 17.1, 17.0, 13.6, 8.6. HRMS-ESI $[M+H]^+$ calcd for $C_{31}H_{36}N_3O_{12}S$ 674.2025, found 674.2026.

5′-*O*-2-(2-nitro-4-ethyl-5-thiophenyl-phenyl)propyloxycarbonyl-2′-*O*-propionyloxymethyl *N*^{4}-acetyl-cytidine (7b)

7b was prepared from nucleoside **6b** (2.11 g, 2.55 mmol) according to **general procedure C**. Compound **7b** was isolated as a white foam (1.5 g, 82%). ^{1}H-NMR (600 MHz, DMSO-d_6) δ 10.94

(s, 1H, NH), 8.00 (t, *J* = 7.2, 1H, H_6), 7.81 (s, 1H, H_5 NPPOC), 7.45-7.40 (m, 5H, SPhNPPOC), 7.17 (dd, *J* = 7.5, 3.3, 1H, H_5), 7.06 (d, *J* = 3.3, 1H, H_2 NPPOC), 5.82 (t, *J* = 3.1, 1H, $H_{1'}$), 5.51 (s, 1H, 3′OH), 5.40 (dd, *J* = 6.4, 1.6, 1H, H_a OCH_2O), 5.31 (dd, *J* = 6.4, 1.7, 1H, H_b OCH_2O), 4.35 (dt, *J* = 12.0, 2.7, 1H, $H_{5'}$), 4.30-4.25 (m, 2H, $H_{4'}$, $H_{5'}$), 4.13 (dt, *J* = 10.7, 6.0, 1H, $OC\mathit{H_2}CH(CH_3)PhNO_2$), 4.05-3.99 (m, 3H, $OC\mathit{H_2}CH(CH_3)PhNO_2$, $H_{2'}$, $H_{3'}$), 3.55-3.51 (m, 1H, $OCH_2C\mathit{H}(CH_3)PhNO_2$), 2.76-2.72 (m, 2H, CH_2 (SPhNPPOC)), 2.32-2.27 (m, 2H, CH_2 PrOM), 2.10 (d, *J* = 0.4, 3H, CH_3 Ac), 1.20 (dt, *J* = 7.5, 3.7, 3H, CH_3 (SPhNPPOC)), 1.06 (dd, *J* = 6.9, 2.1, 3H, $OCH_2CH(C\mathit{H_3})PhNO_2$), 0.99 (td, *J* = 7.5, 1.1, 3H, CH_3 PrOM). ^{13}C-NMR (151 MHz, DMSO-d_6) δ 173.2, 171.0, 162.6, 154.3, 154.0, 147.9, 147.8, 145.7, 145.1, 142.0, 141.7, 134.7, 134.6, 132.6, 131.6, 130.0, 128.8, 128.5, 128.3, 124.1, 95.6, 89.8 and 89.7, 87.5, 80.6, 70.9, 70.8, 68.4, 67.0, 32.4, 26.8, 25.6, 24.3, 17.1 and 17.0, 13.7, 8.6. HRMS-ESI $[M+H]^+$ calcd for $C_{33}H_{39}N_4O_{12}S$ 715.2280, found 715.2282.

5′-*O*-2-(2-nitro-4-ethyl-5-thiophenyl-phenyl)propyloxycarbonyl-2′-*O*-propionyloxymethyl-*N*^{6}-phenoxyacetyl-adenosine (7c)

7c was prepared from nucleoside **6c** (3.10 g, 3.28 mmol) according to **general procedure C**. Compound **7c** was isolated as a white foam (2.50 g, 92%).^{1}H-NMR (600 MHz, DMSO-d_6) δ 10.96 (s, 1H, NH), 8.68-8.65 (m, 2H, H_2 , H_8), 7.80 (d, *J* = 6.1, 1H, H_5 NPPOC), 7.44-7.37 (m, 5H, SPhNPPOC), 7.30 (t, *J* = 7.9, 2H, (o) Pac), 7.03 (d, *J* = 3.7, 1H, H_2 NPPOC), 6.96 (dd, *J* = 7.4, 4.1, 3H, (m) (p) Pac), 6.16 (d, *J* = 5.3, 1H, $H_{1'}$), 5.68 (d, *J* = 5.4, 1H, 3′OH), 5.34 (d, *J* = 6.5, 1H, OCH_2O), 5.20 (dd, *J* = 6.5, 1.8, 1H, OCH_2O), 5.03 (s, 2H, $NHCOCH_2Ph$), 4.97 (td, *J* = 5.2, 2.7, 1H, $H_{2'}$), 4.46-4.41 (m, 1H, $H_{3'}$), 4.37 (dt, *J* = 11.5, 3.5, 1H, $H_{5'}$), 4.28 (dt, *J* = 11.7, 7.2, 1H, $H_{5'}$), 4.14-4.07 (m, 2H, $OC\mathit{H_2}CH(CH_3)PhNO_2$, $H_{4'}$), 3.99 (ddd, *J* = 12.7, 10.8, 7.4, 1H, $OC\mathit{H_2}CH(CH_3)PhNO_2$), 3.49 (ddd, *J* = 13.2, 6.9, 3.4, 1H, $OCH_2C\mathit{H}(CH_3)PhNO_2$), 2.73 (dq, *J* = 11.5, 3.7, 2H, $C\mathit{H_2}$ (SPhNPPOC)), 2.10-1.98 (m, 2H, CH_2 PrOM), 1.20-1.17 (m, 3H, CH_3 (SPhNPPOC)), 1.02 (dd, *J* = 6.9, 1.7, 3H, $OCH_2CH(C\mathit{H_3})PhNO_2$), 0.82 (td, *J* = 7.5, 1.7, 3H, CH_3 PrOM). ^{13}C-NMR (151 MHz, DMSO-d_6) δ 172.9, 167.3, 157.8, 153.9, 151.6, 151.4, 149.2, 147.7, 143.3, 141.9, 141.7, 134.6, 132.6, 131.5, 130.0, 129.9, 129.5, 128.9, 128.8, 128.7, 128.3, 128.2, 124.1, 123.4, 121.1, 114.5, 87.8, 86.3, 81.8, 79.9, 70.8, 69.1, 67.3, 67.2, 32.4, 26.5, 25.5, 17.1, 13.6, 8.4. HRMS-ESI $[M+H]^+$ calcd for $C_{40}H_{43}N_6O_{12}S$ 831.2654, found 831.2656.

5′-*O*-2-(2-nitro-4-ethyl-5-thiophenyl-phenyl)propyloxycarbonyl-2′-*O*-propionyloxymethyl-*N*^{2}-*iso*propylphenoxyacetyl-guanosine (7d)

7d was prepared from nucleoside **2d** (1.28 g, 1.28 mmol) according to **general procedure C**. Compound **7d** was isolated as a white foam (1.05 g, 93%).^{1}H-NMR (600 MHz, DMSO-d_6) δ 11.82 (s, 1H, NH), 11.71 (s, 1H, NH *i*PrPac), 8.19 (d, *J* = 7.4, 1H, H_8), 7.80 (d, *J* = 2.6, 1H, H_5 NPPOC), 7.48-7.36 (m, 5H, SPhNPPOC), 7.16 (d, *J* = 8.4, 2H, (m) *i*PrPac), 7.03 (d, *J* = 3.9, 1H, H_2 NPPOC), 6.92-6.86 (m, 2H, (o) *i*PrPac), 5.95 (dd, *J* = 5.7, 1.5, 1H, $H_{1'}$), 5.61 (d, *J* = 4.9, 1H, 3′OH), 5.33 (d, *J* = 6.5, 1H, OCH_2O), 5.21 (dd, *J* = 6.5, 2.4, 1H, OCH_2O), 4.82 (s, 2H, NHCOCH_2Ph), 4.74 (t, *J* = 5.4, 1H, $H_{2'}$), 4.40-4.30 (m, 2H, $H_{3'}$, $H_{5'}$), 4.24 (ddd, *J* = 11.5, 6.9, 4.5, 1H, $H_{5'}$), 4.15- 4.05 (m, 2H, CH_2CH(CH_3)PhNO_2, $H_{4'}$), 4.00 (td, *J* = 10.4, 7.4, 1H, CH_2CH(CH_3)PhNO_2), 3.56-3.46 (m, 1H, OCH_2C*H*(CH_3)PhNO_2), 2.84 (dq, *J* = 13.8, 6.9, 1H, C*H*(CH_3)$_2$ *i*PrPac), 2.74 (q, *J* = 7.5, 2H, CH_2 (SPhNPPOC)), 2.25-2.03 (m, 2H, CH_2 PrOM), 1.20 (d, *J* = 7.5, 3H, CH_3 (SPhNPPOC)), 1.16 (d, *J* = 6.9, 6H, CH(CH_3)$_2$ *i*PrPac), 1.02 (t, *J* = 9.4, 3H, OCH_2CH(CH_3)PhNO_2), 0.88 (dd, *J* = 7.9, 7.1, 3H, CH_3 PrOM). ^{13}C-NMR (151 MHz, DMSO-d_6) δ 172.9, 155.7, 154.8, 154.0, 148.5, 147.7, 141.9 and 141.8, 141.3, 138.0, 134.6, 132.6, 131.5, 130.0, 128.8 and 128.7, 128.2, 127.2, 124.1, 120.5, 114.4, 87.8, 85.4, 81.9, 81.8, 80.3, 70.9, 70.8, 69.1, 67.6, 67.5, 66.5, 32.6, 32.4, 26.6, 25.5, 24.1, 17.1, 17.0, 13.6, 8.5. HRMS-ESI $[M+H]^+$ calcd for $C_{43}H_{49}N_6O_{13}S$ 889.3073 found 889.3080.

5′-*O*-2-(2-nitro-4-ethyl-5-thiophenyl-phenyl)propyloxycarbonyl-2′-*O*-propionyloxymethyl-3′-*O*-(2-cyanoethyl)-(*N,N*-di*iso*propyl)-phosphoramidite uridine (8a)

8a was prepared from nucleoside **7a** (1.08 g, 1.60 mmol), *N,N*-di*iso*propylchlorophosphoramidite (895 µL, 4.01 mmol) and di*iso*propylethylamine (782 µL, 4.49 mmol) according to **general procedure D**. Compound **8a** was isolated as a white foam (1.23 g, 88 %). ^{31}P-NMR (162 MHz, $CDCl_3$) δ 150.76, 150.66, 150.43, 150.37. HRMS-ESI $[M+H]^+$ calcd for $C_{40}H_{53}N_5O_{13}PS$ 874.3093, found 874.3085.

5′-*O*-2-(2-nitro-4-ethyl-5-thiophenyl-phenyl)propyloxycarbonyl-2′-*O*-propionyloxymethyl-3′-*O*-(2-cyanoethyl)-(*N,N*-di*iso*propyl)-phosphoramidite *N*^{4}-acetyl-cytidine (8b)

8b was prepared from nucleoside **7b** (1 g, 1.4 mmol), *N,N*-di*iso*propylchlorophosphoramidite (781 µL, 3.5 mmol) and di*iso*propylethylamine (682 µL, 3.92 mmol) according to **general procedure D**. Compound **8b** was isolated as a white foam (946 mg, 74 %). ^{31}P-NMR (162 MHz, $CDCl_3$) δ 150.16, 150.04, 150.00. HRMS-ESI $[M+H]^+$ calcd for $C_{42}H_{56}N_6O_{13}PS$ 915.3019, found 915.3008.

5′-*O*-2-(2-nitro-4-ethyl-5-thiophenyl-phenyl)propyloxycarbonyl-2′-*O*-propionyloxymethyl-3′-*O*-(2-cyanoethyl)-(*N,N*-di*iso*propyl)-phosphoramidite N^6-phenoxyacetyl-adenosine (8c)

8c was prepared from nucleoside **7c** (1.06 g, 1.28 mmol), *N,N*-di*iso*propylchlorophosphoramidite (712 µL, 3.19 mmol) and di*iso*propylethylamine (622 µL, 3.57 mmol) according to **general procedure D**. Compound **8c** was isolated as a white foam (946 mg, 72 %). ^{31}P-NMR (162 MHz, $CDCl_3$) δ 151.03, 150.97, 150.84. HRMS-ESI $[M+H]^+$ calcd for $C_{49}H_{60}N_8O_{14}PS$ 1047.3682, found 1047.3665.

5′-*O*-2-(2-nitro-4-ethyl-5-thiophenyl-phenyl)propyloxycarbonyl-2′-*O*-propionyloxymethyl-3′-*O*-(2-cyanoethyl)-(*N,N*-di*iso*propyl)-phosphoramidite N^2-*iso*propylphenoxyacetyl-guanosine (8d)

8d was prepared from nucleoside **7d** (1.04 g, 1.17 mmol), *N,N*-di*iso*propylchlorophosphoramidite (653 µL, 2.92 mmol) and di*iso*propylethylamine (570 µL, 3.28 mmol) according to **general procedure D**. The product was isolated as a white foam (899 mg, 71 %). ^{31}P-NMR (162 MHz, $CDCl_3$) δ 151.20, 150.79, 150.77. HRMS-ESI $[M+H]^+$ calcd for $C_{52}H_{66}N_8O_{14}PS$ 1089.4151, found 1089.4145.

NMR Spectra of compounds 2 to 8

^{1}H-NMR spectrum of 3′-*O*-TBDMS 2′-*O*-propionyloxymethyl uridine (**2a**)

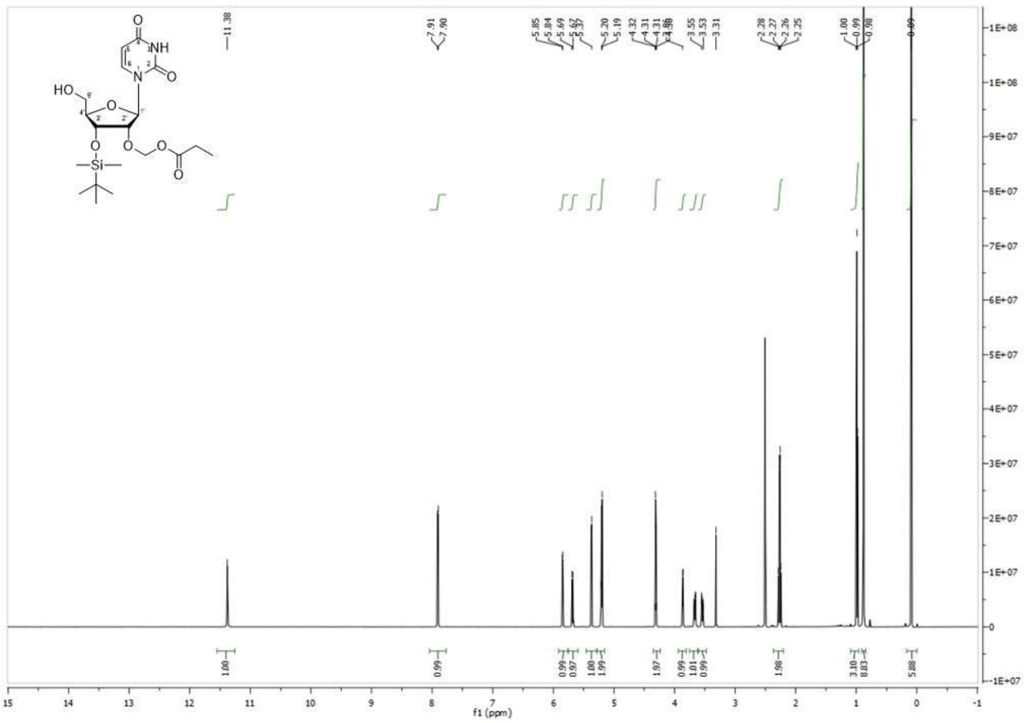

^{13}C-NMR spectrum of 3′-*O*-TBDMS 2′-*O*-propionyloxymethyl uridine (**2a**)

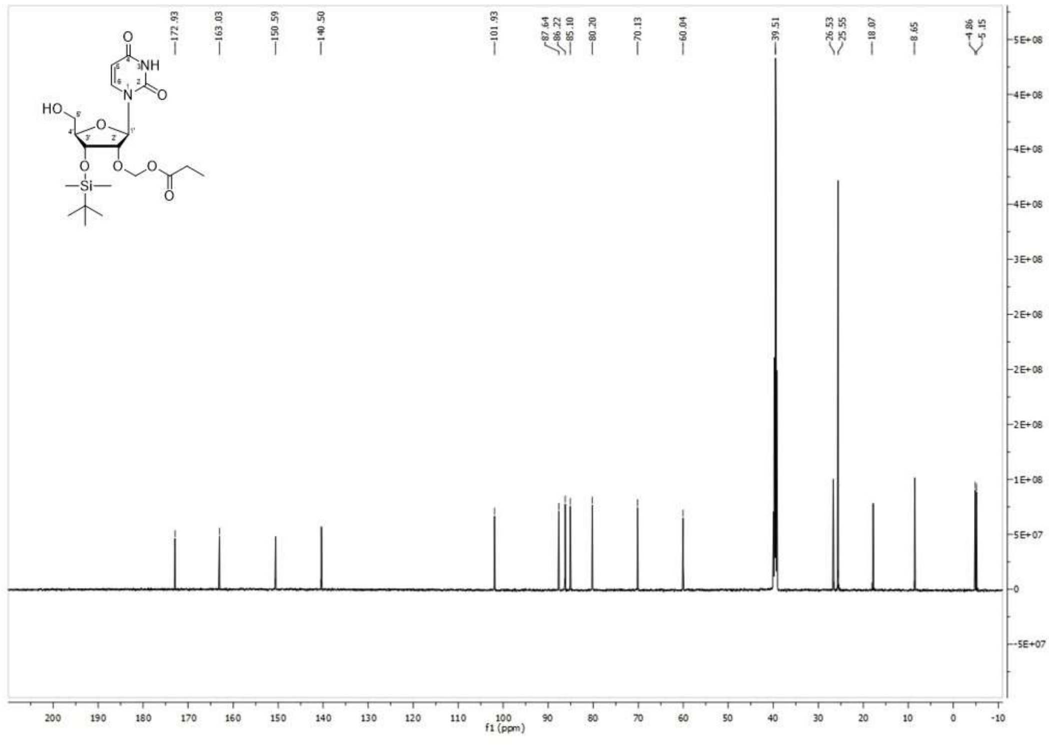

^{1}H-NMR spectrum of 3′-*O*-TBDMS-2′-*O*-propionyloxymethyl-*N*[4]-acetyl-cytidine (**2b**)

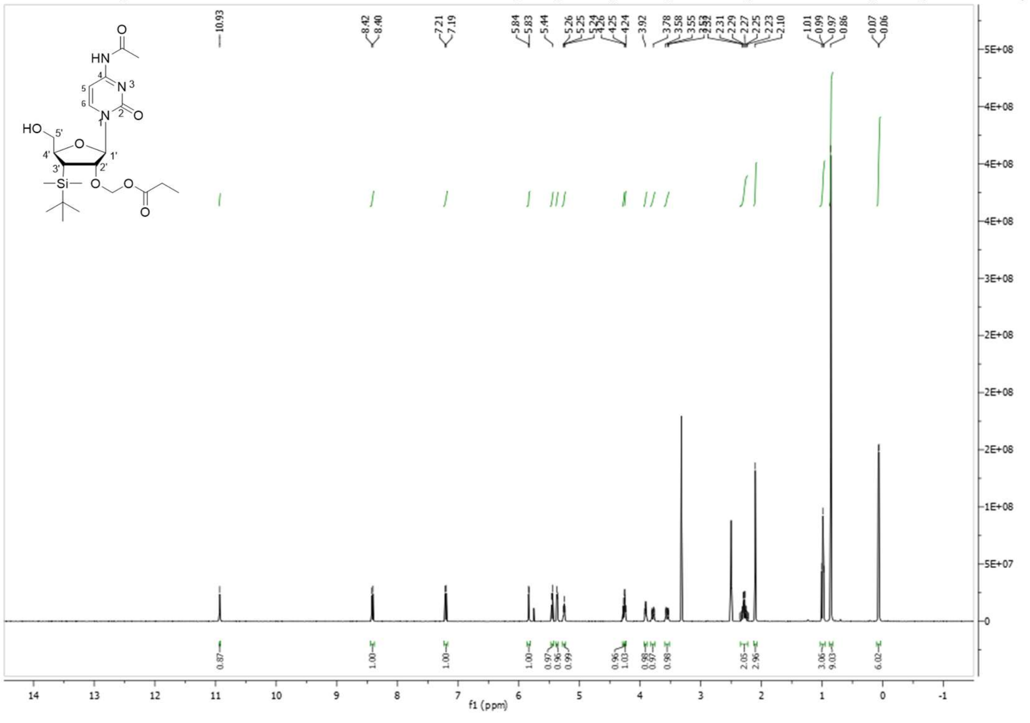

^{13}C-NMR spectrum of 3′-*O*-TBDMS-2′-*O*-propionyloxymethyl-*N*[4]-acetyl-cytidine (**2b**)

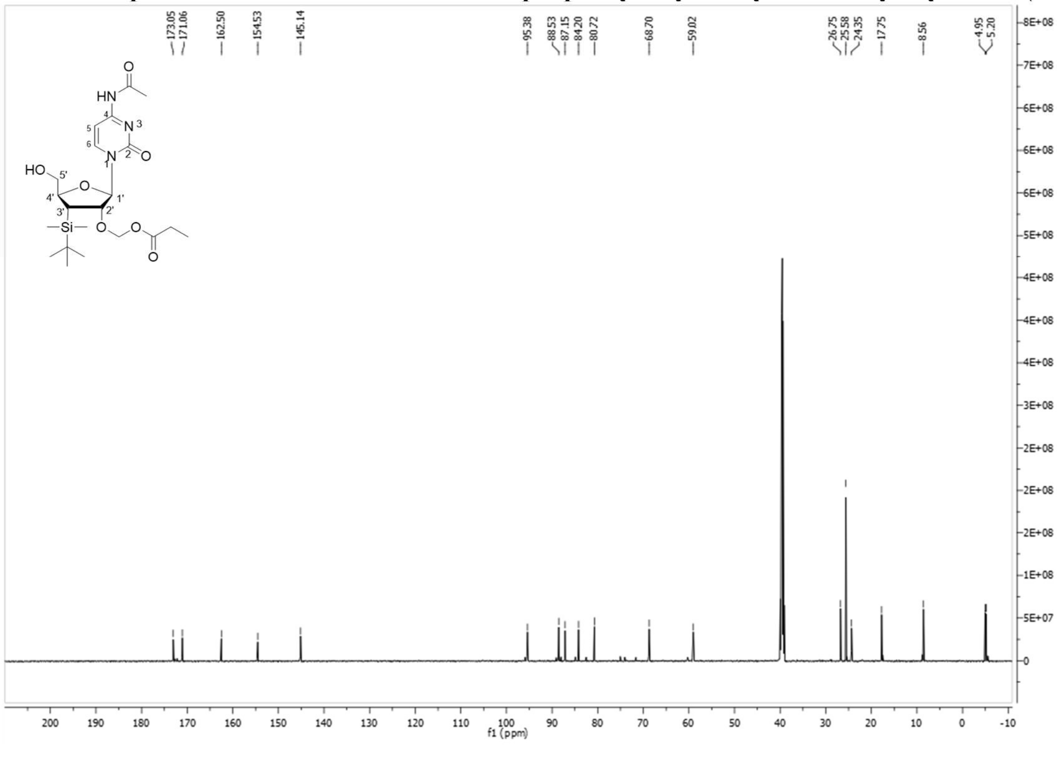

^{1}H-NMR spectrum of 3′-*O*-TBDMS-2′-*O*-propionyloxymethyl-*N*^{6}-Pac-adenosine (**2c**)

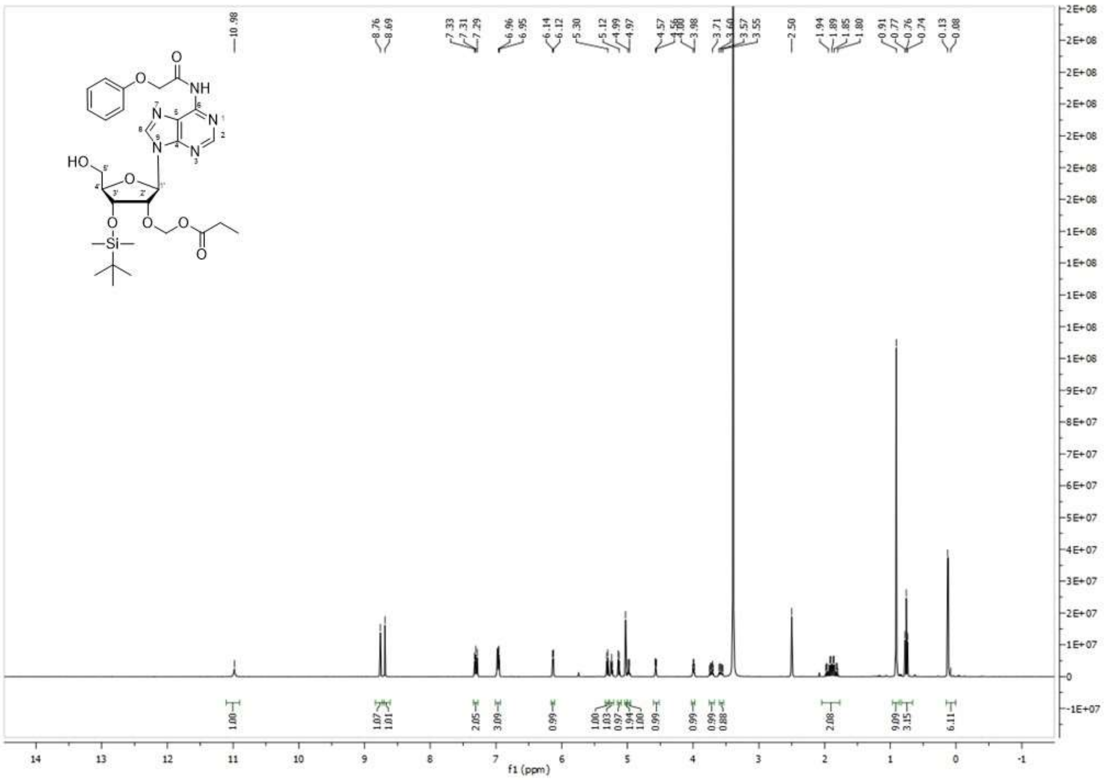

^{13}C-NMR spectrum of 3′-*O*-TBDMS-2′-*O*-propionyloxymethyl-*N*^{6}-Pac-adenosine (**2c**)

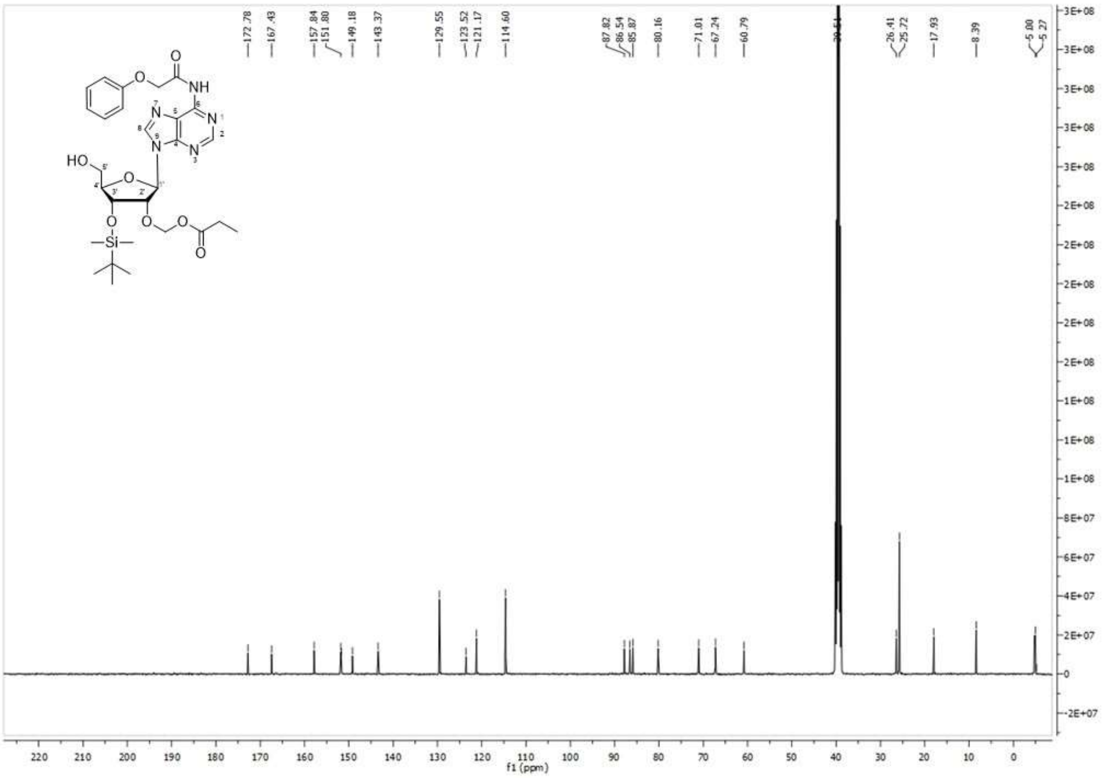

^{1}H-NMR spectrum of 3′-*O*-TBDMS-2′-*O*-propionyloxymethyl-*N*[2]-*i*PrPac-guanosine (**2d**)

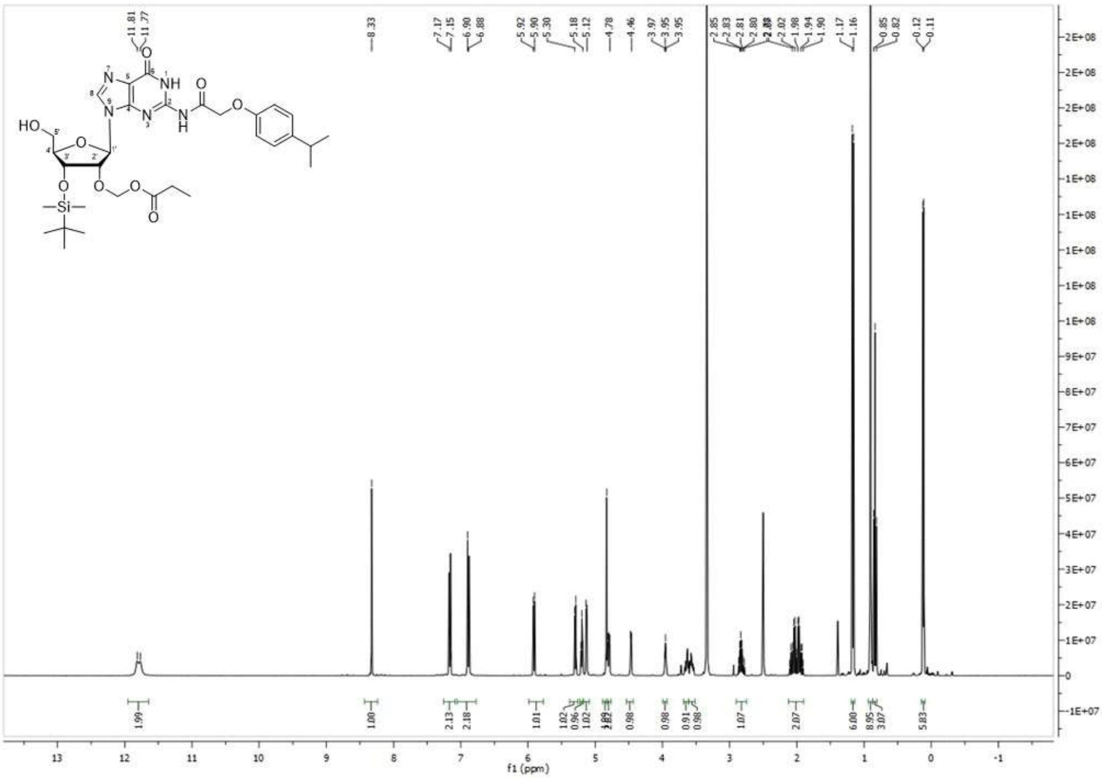

^{13}C-NMR spectrum of 3′-*O*-TBDMS-2′-*O*-propionyloxymethyl-*N*[2]-*i*PrPac-guanosine (**2d**)

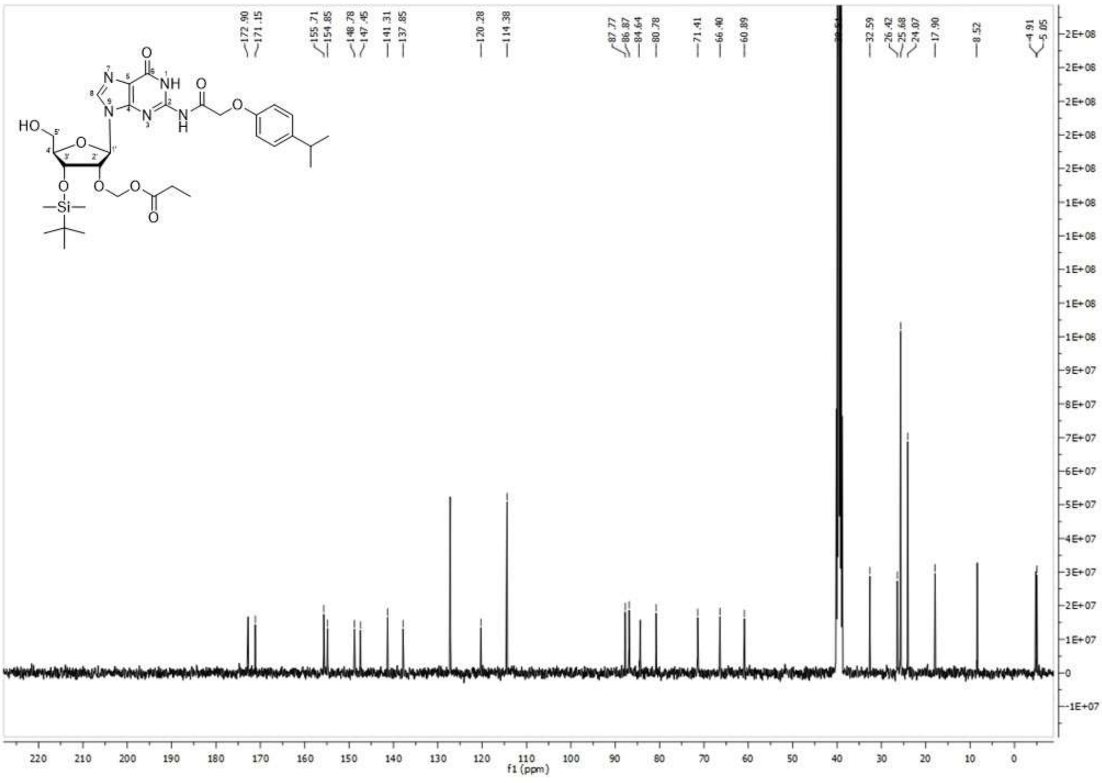

^{1}H-NMR spectrum of 5′-O-2-(2-nitrophenyl)propyloxycarbonyl-3′-O-TBDMS-2′-O-propionyloxymethyl uridine (**3a**)

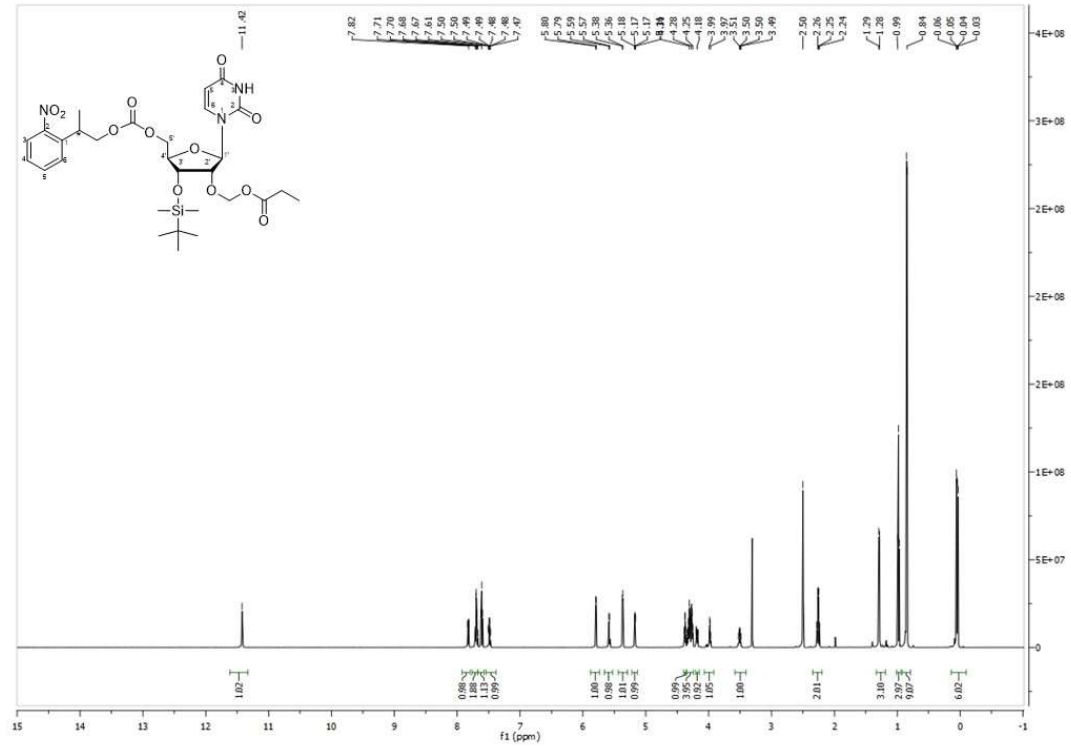

^{13}C-NMR spectrum of 5′-O-2-(2-nitrophenyl)propyloxycarbonyl-3′-O-TBDMS-2′-O-propionyloxymethyl uridine (**3a**)

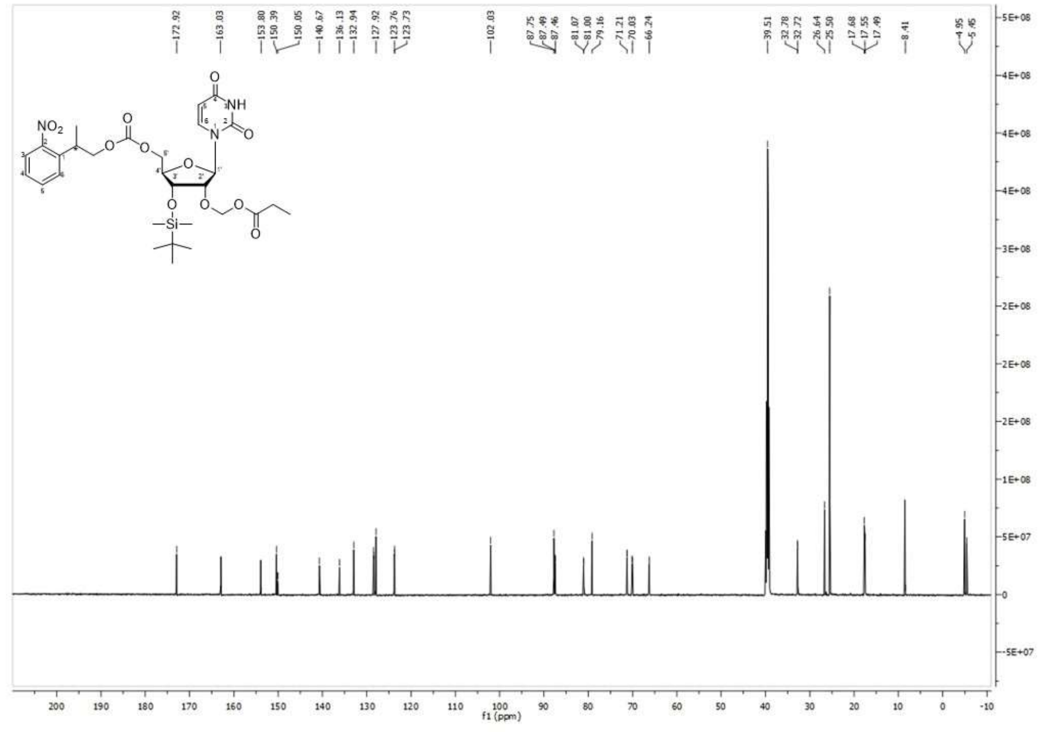

^{1}H-NMR spectrum of 5′-*O*-2-(2-nitrophenyl)propyloxycarbonyl-3′-*O*-TBDMS-2′-*O*-propionyloxymethyl-*N*[4]-acetyl-cytidine (**3b**)

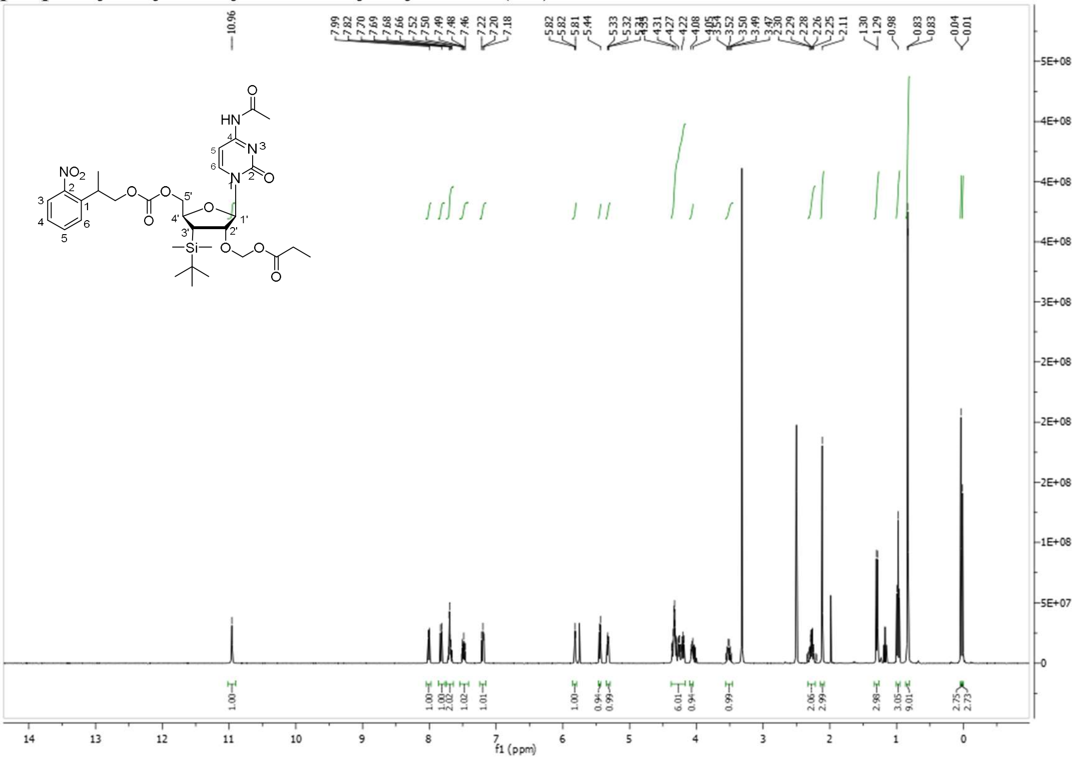

^{13}C-NMR spectrum of 5′-*O*-2-(2-nitrophenyl)propyloxycarbonyl-3′-*O*-TBDMS-2′-*O*-propionyloxymethyl-*N*[4]-acetyl-cytidine (**3b**)

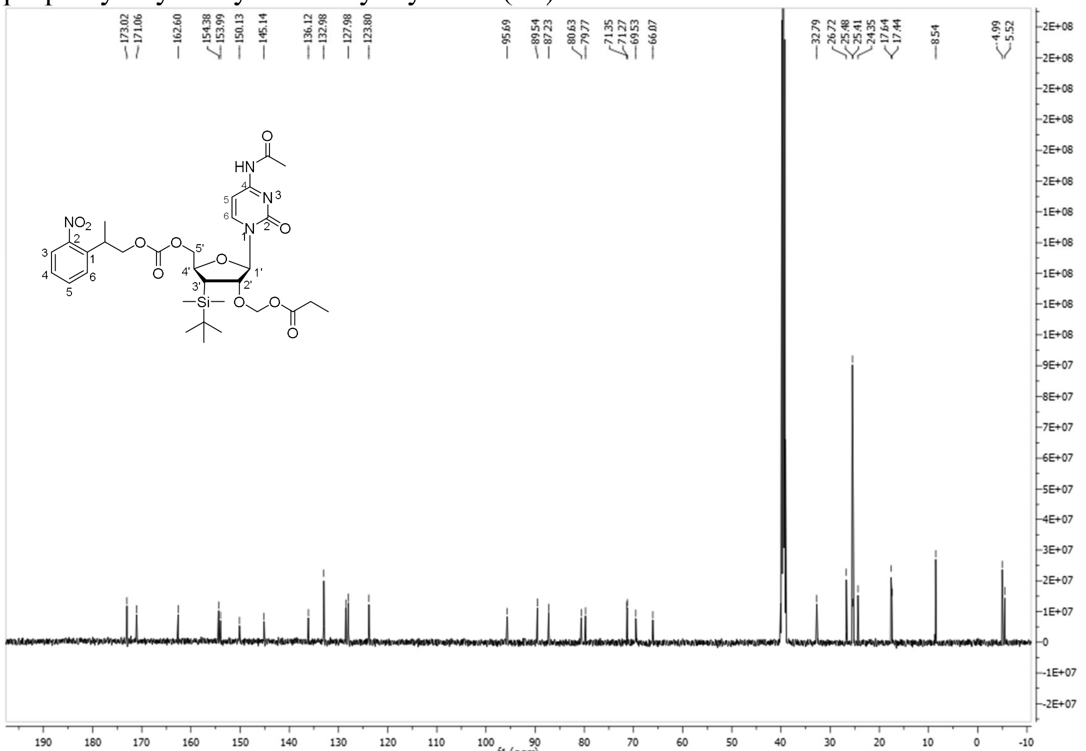

^{1}H-NMR spectrum of 5′-*O*-2-(2-nitrophenyl)propyloxycarbonyl-3′-*O*-TBDMS-2′-*O*-propionyloxymethyl *N*^{6}-Pac-adenosine (**3c**)

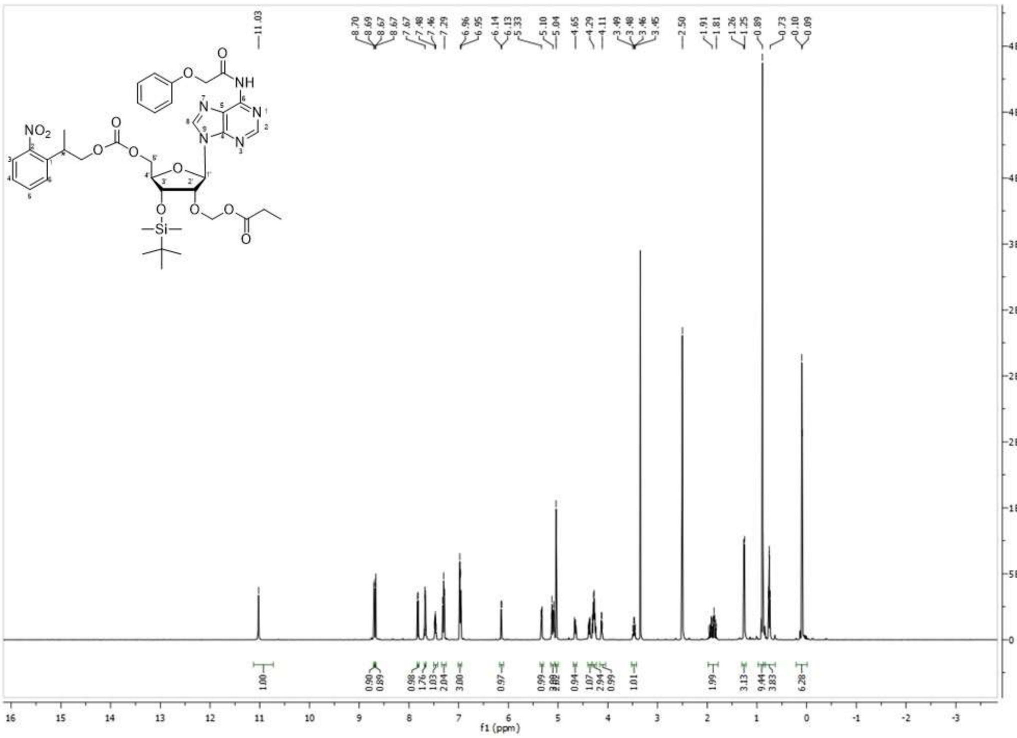

^{13}C-NMR spectrum of 5′-*O*-2-(2-nitrophenyl)propyloxycarbonyl-3′-*O*-TBDMS-2′-*O*-propionyloxymethyl *N*^{6}-Pac-adenosine (**3c**)

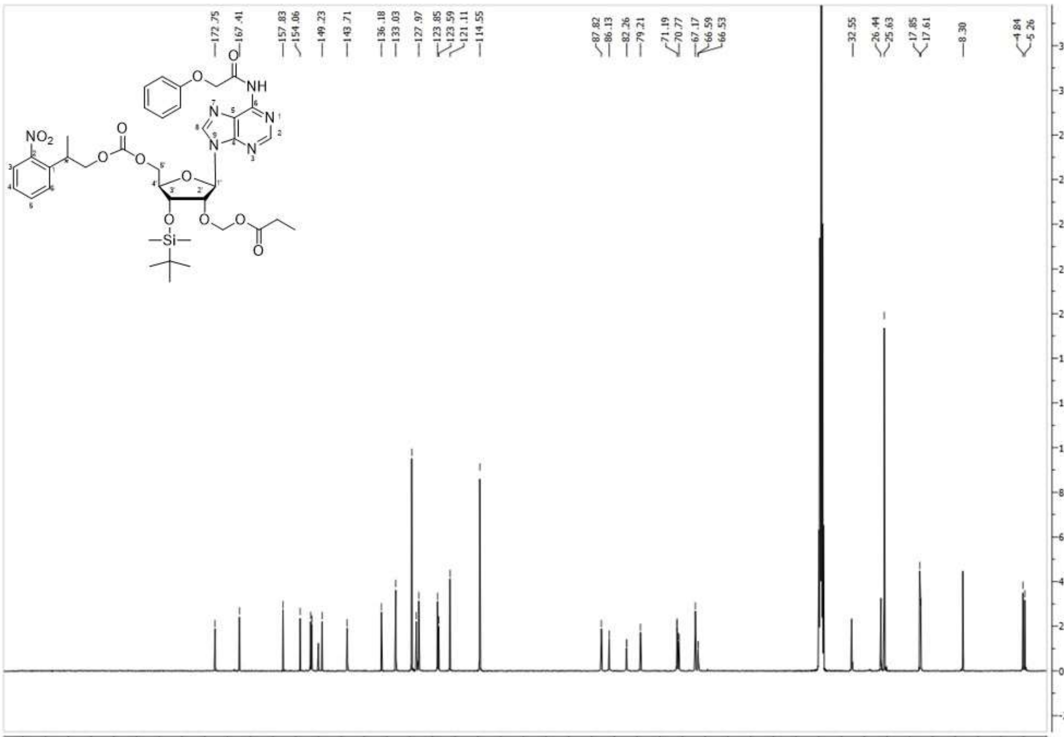

^{1}H-NMR spectrum of 5′-*O*-2-(2-nitrophenyl)propyloxycarbonyl-3′-*O*-TBDMS-2′-*O*-propionyloxymethyl *N*^{2}-*i*PrPac-guanosine (**3d**)

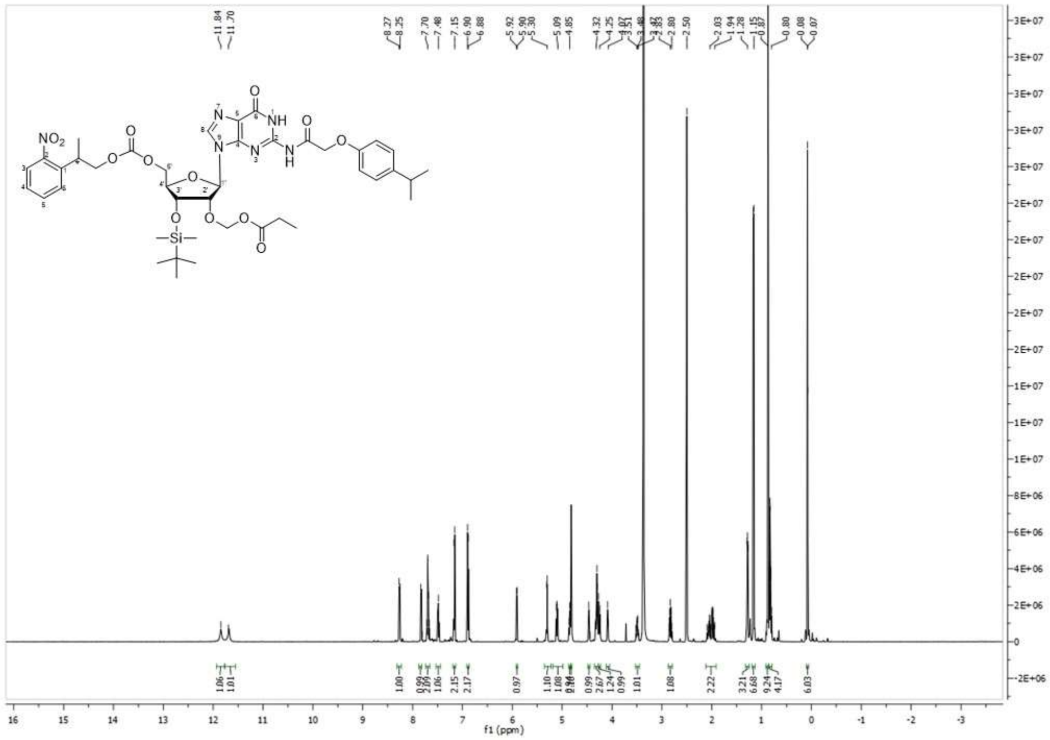

^{13}C-NMR spectrum of 5′-*O*-2-(2-nitrophenyl)propyloxycarbonyl-3′-*O*-TBDMS-2′-*O*-propionyloxymethyl *N*^{2}-*i*PrPac-guanosine (**3d**)

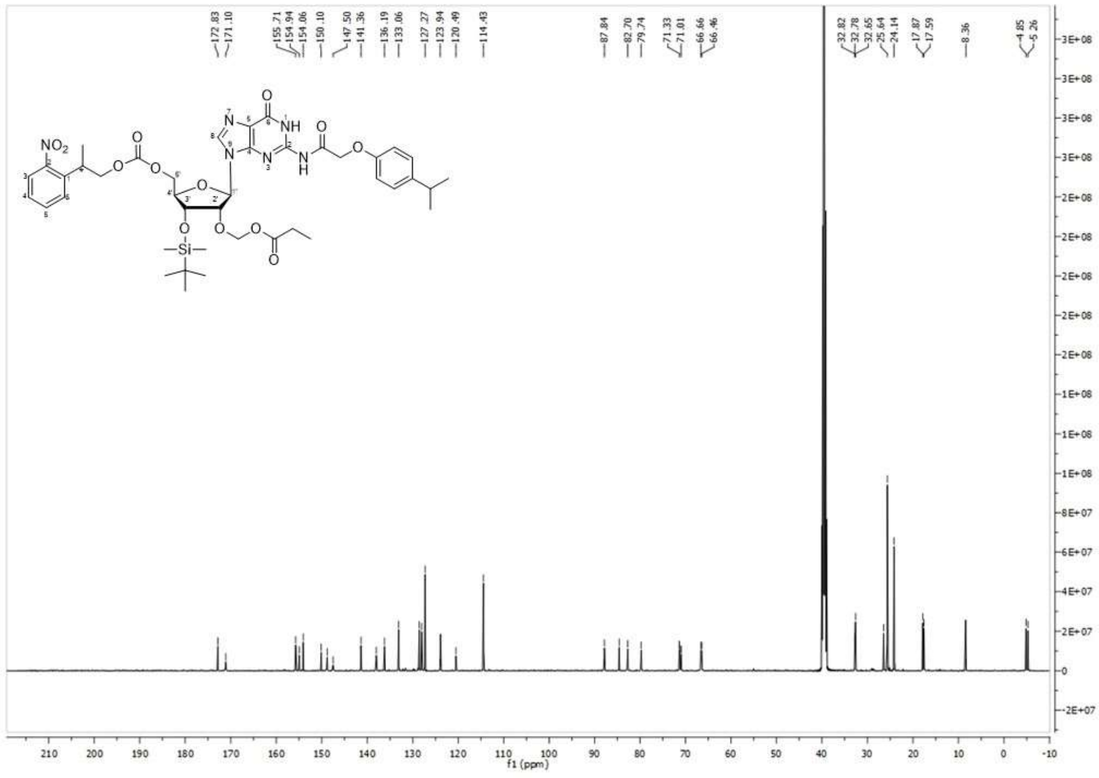

^{1}H-NMR spectrum of 5′-*O*-2-(2-nitrophenyl)propyloxycarbonyl-2′-*O*-propionyloxymethyl uridine (**4a**)

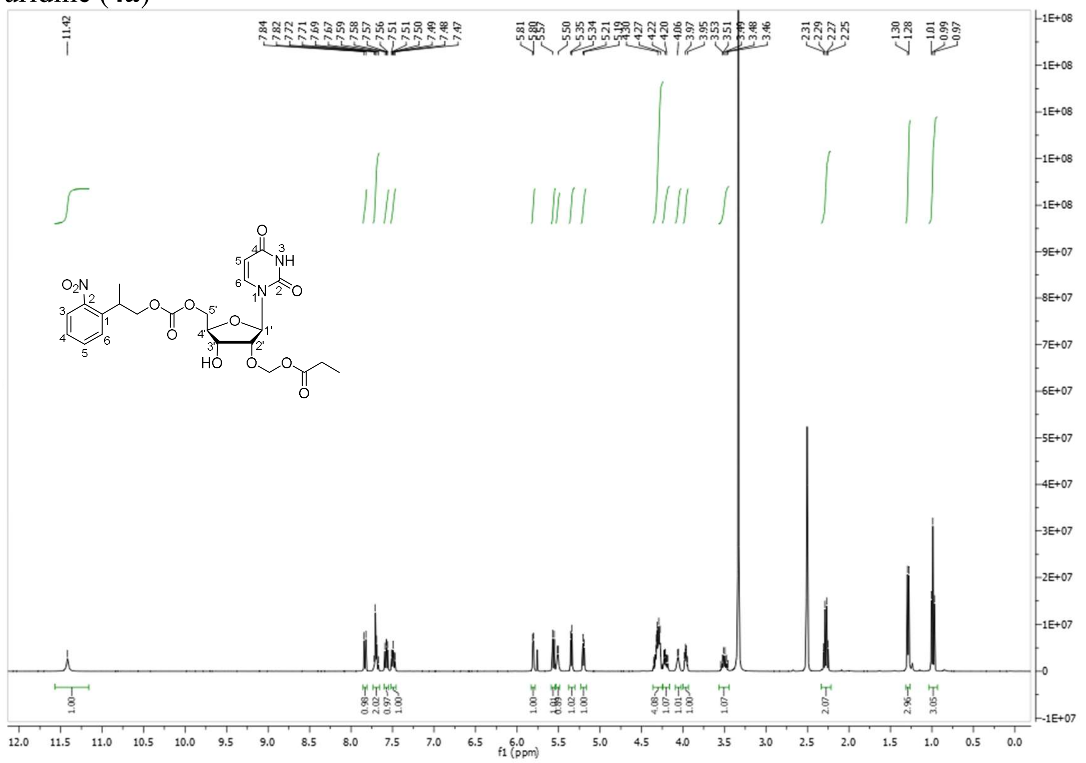

^{13}C-NMR spectrum of 5′-*O*-2-(2-nitrophenyl)propyloxycarbonyl-2′-*O*-propionyloxymethyl uridine (**4a**)

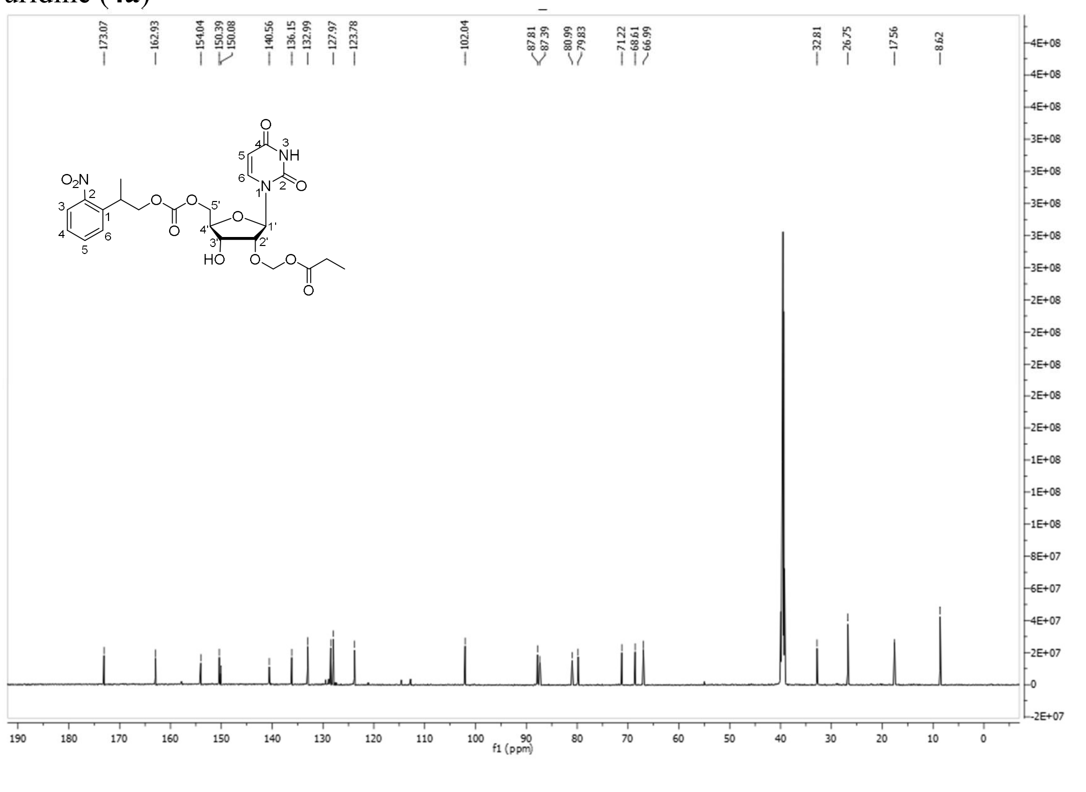

^{1}H-NMR spectrum of 5′-*O*-2-(2-nitrophenyl)propyloxycarbonyl-2′-*O*-propionyloxymethyl- N^4-acetyl-cytidine (**4b**)

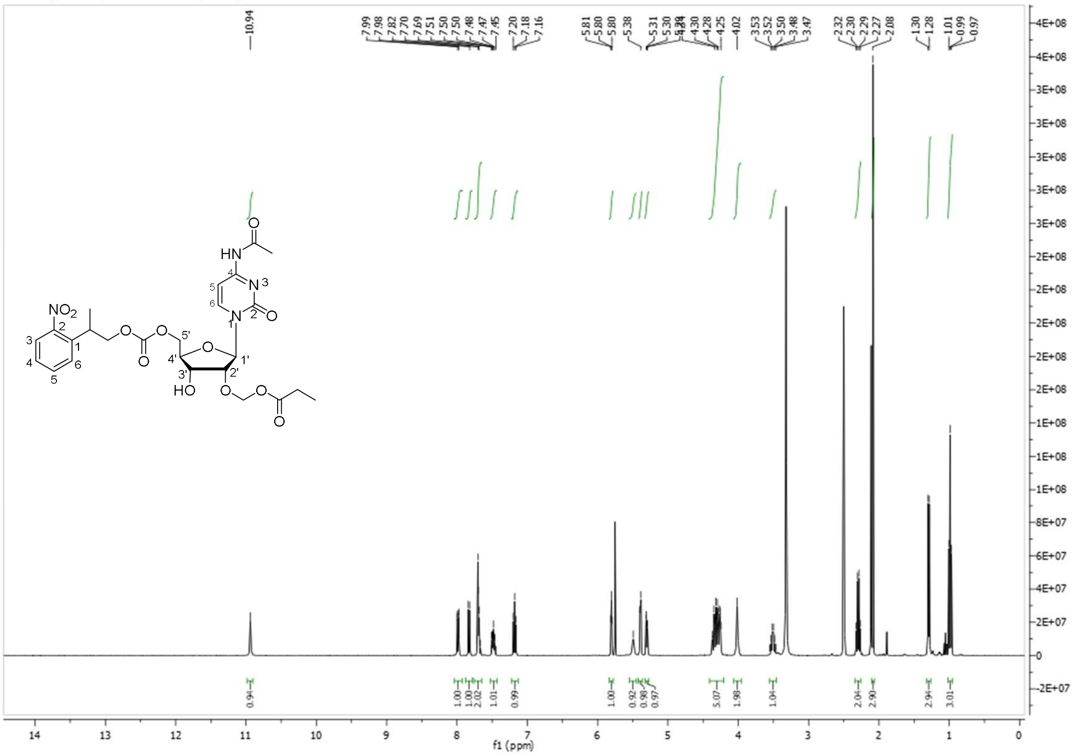

^{13}C-NMR spectrum of 5′-*O*-2-(2-nitrophenyl)propyloxycarbonyl-2′-*O*-propionyloxymethyl-N^4-acetyl-cytidine (**4b**)

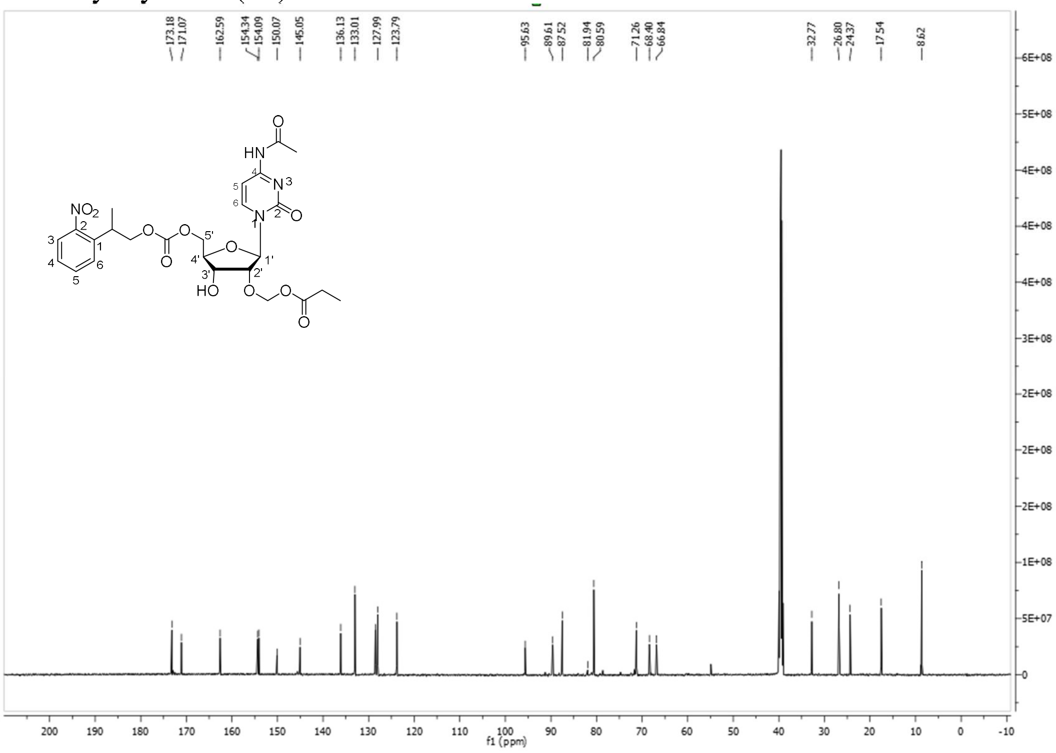

^{1}H-NMR spectrum of 5′-*O*-2-(2-nitrophenyl)propyloxycarbonyl-2′-*O*-propionyloxymethyl *N*^{6}-Pac-adenosine (**4c**)

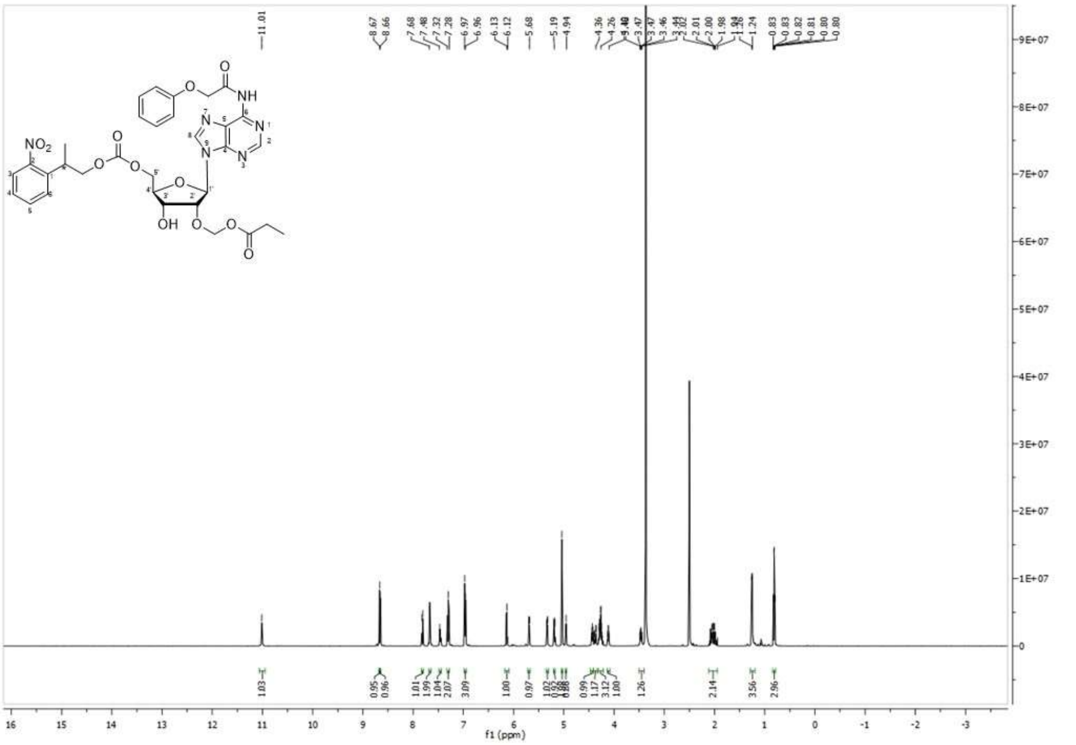

^{13}C-NMR spectrum of 5′-*O*-2-(2-nitrophenyl)propyloxycarbonyl-2′-*O*-propionyloxymethyl *N*^{6}-Pac-adenosine (**4c**)

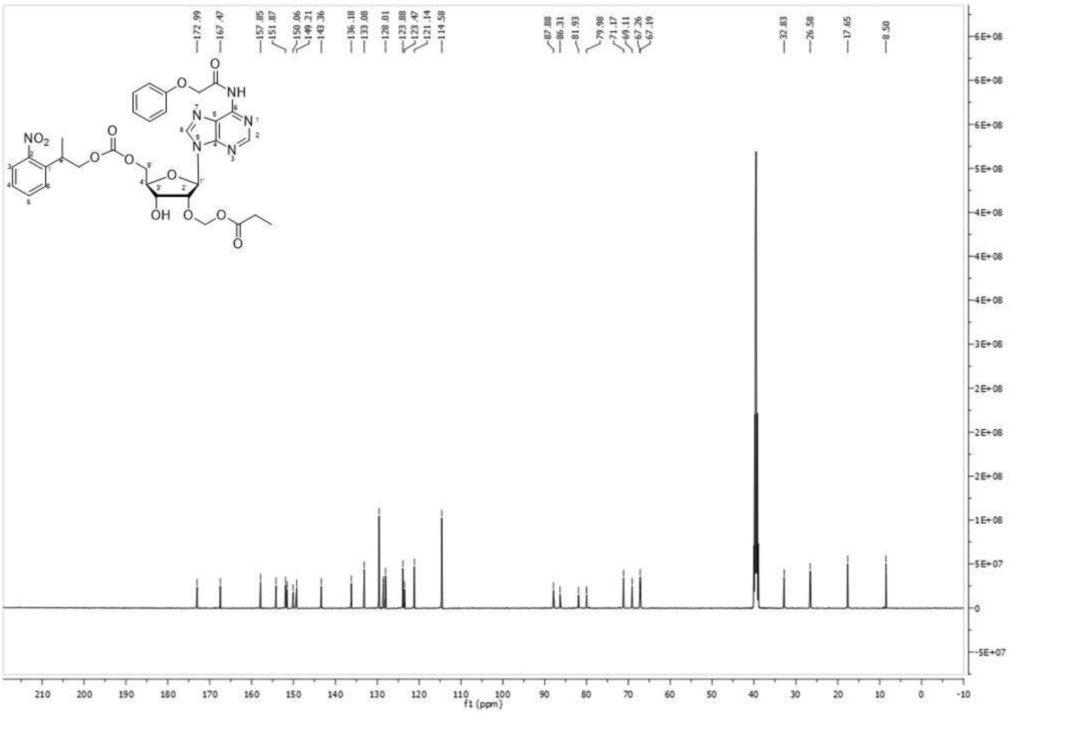

^{1}H-NMR spectrum of 5′-*O*-2-(2-nitrophenyl)propyloxycarbonyl-2′-*O*- propionyloxymethyl N^2-*i*PrPac-guanosine (**4d**)

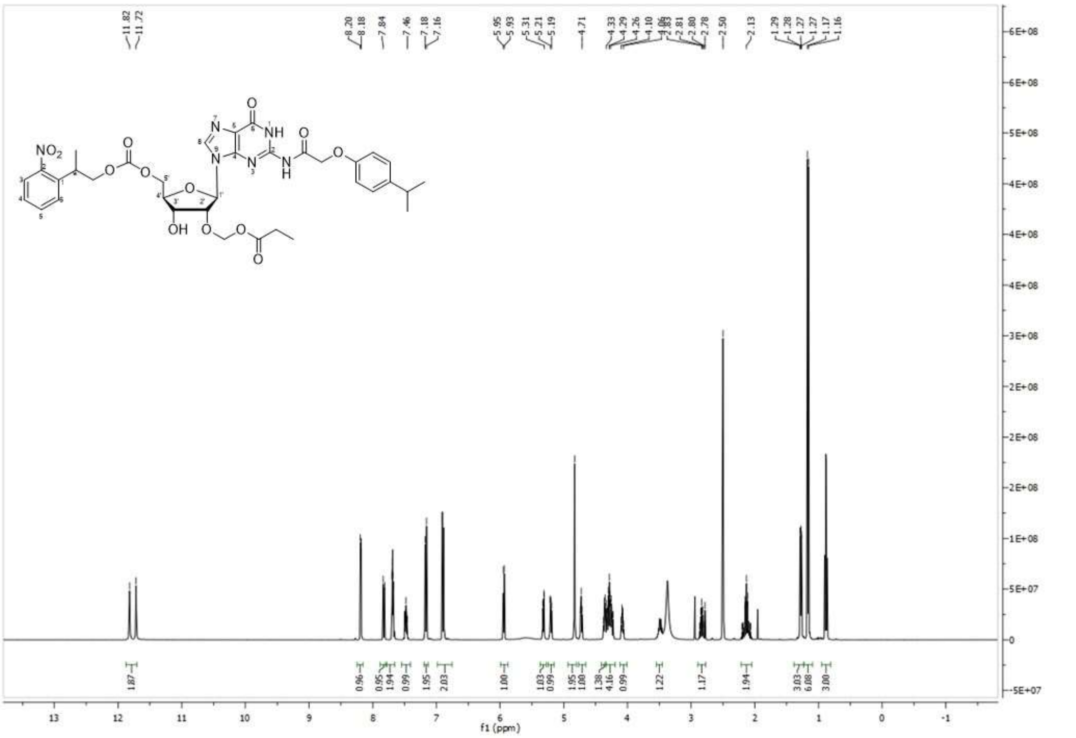

^{13}C-NMR spectrum of 5′-*O*-2-(2-nitrophenyl)propyloxycarbonyl-2′-*O*- propionyloxymethyl N^2-*i*PrPac-guanosine (**4d**)

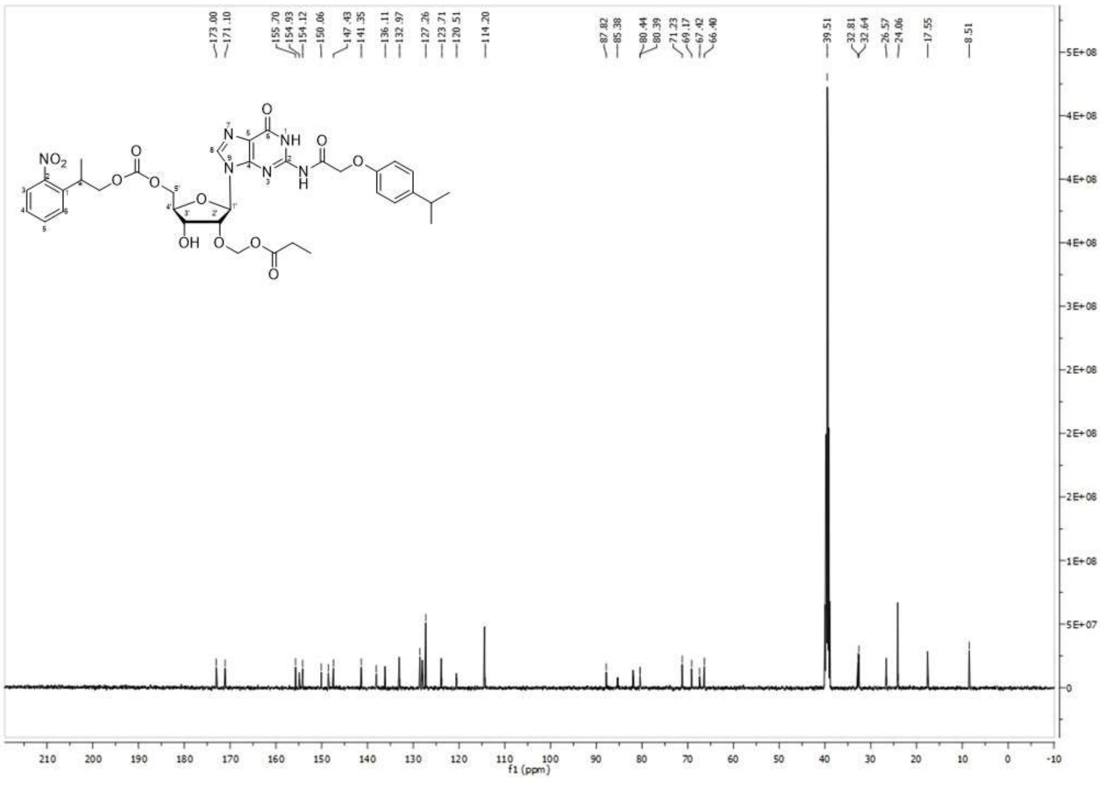

^{31}P-NMR spectrum of 5′-*O*-2-(2-nitrophenyl)propyloxycarbonyl-2′-*O*-propionyloxymethyl-3′-*O*-(2-cyanoethyl)-(*N,N*-di*iso*propyl)-phosphoramidite-uridine (**5a**)

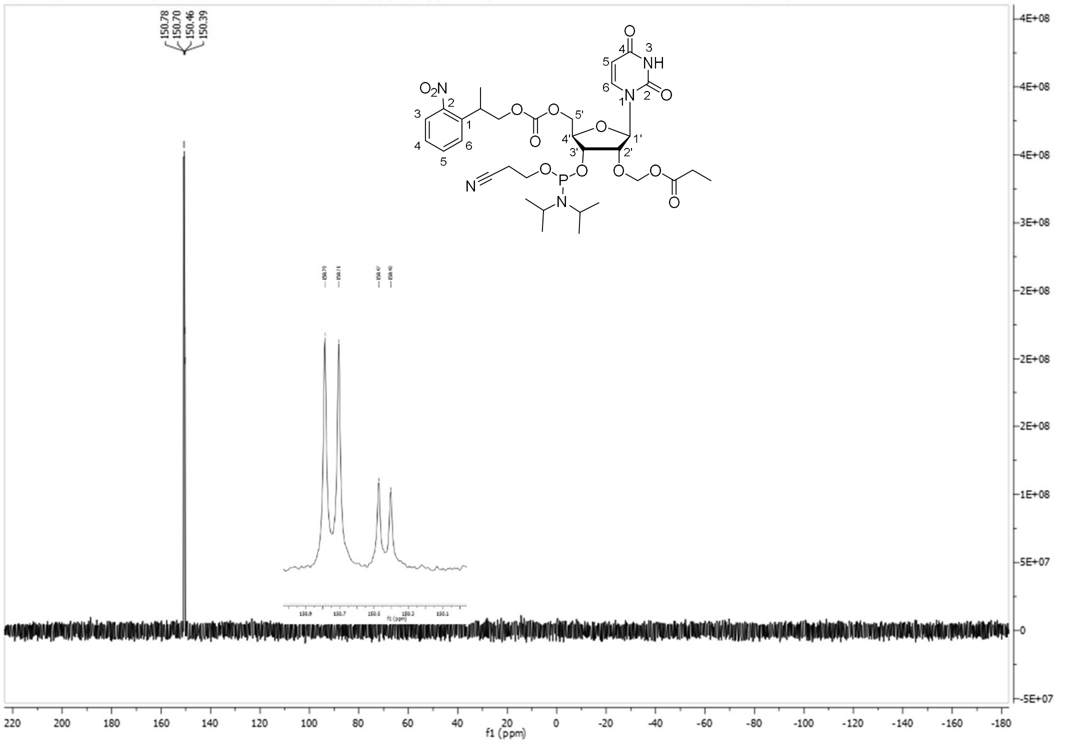

^{31}P-NMR spectrum of 5′-*O*-2-(2-nitrophenyl)propyloxycarbonyl-2′-*O*-propionyloxymethyl-3′-*O*-(2-cyanoethyl)-(*N,N*-di*iso*propyl)-phosphoramidite-*N*^{4}-acetyl-cytidine (**5b**)

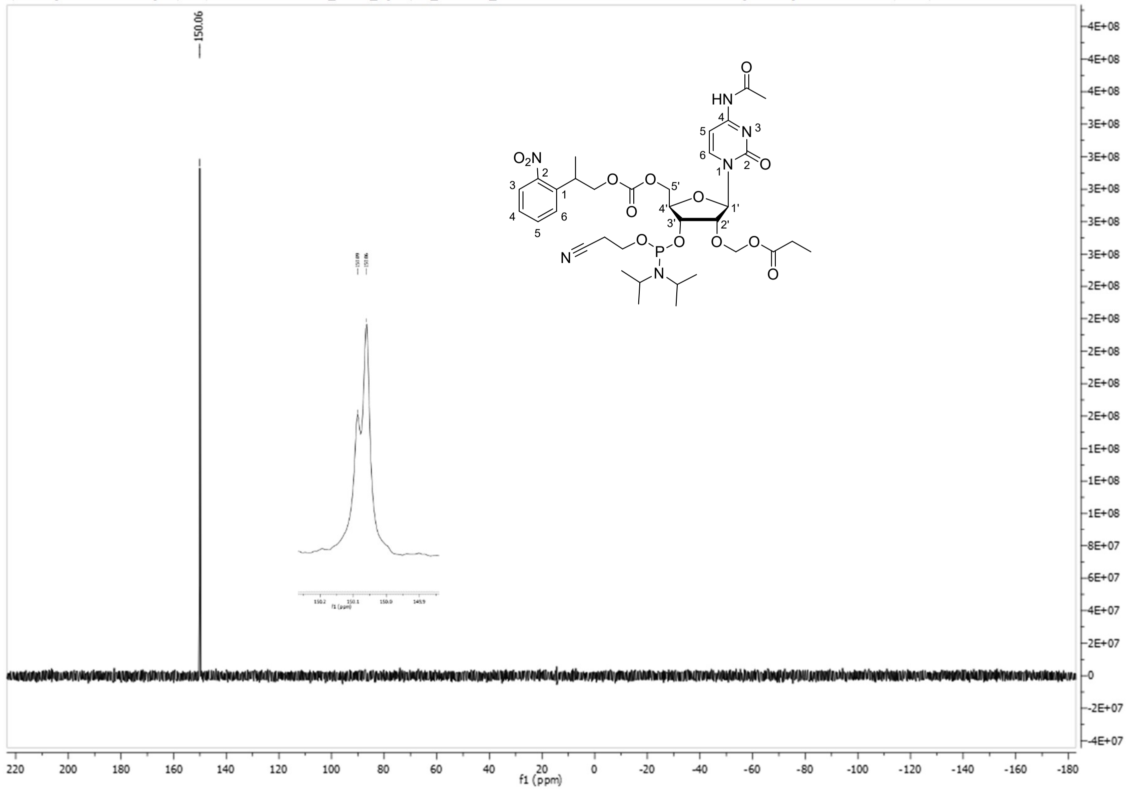

^{31}P-NMR spectrum of 5′-*O*-2-(2-nitrophenyl)propyloxycarbonyl-2′-*O*-propionyloxymethyl-3′-*O*-(2-cyanoethyl)-(*N,N*-di*iso*propyl)-phosphoramidite-N^6-Pac-adenosine (**5c**)

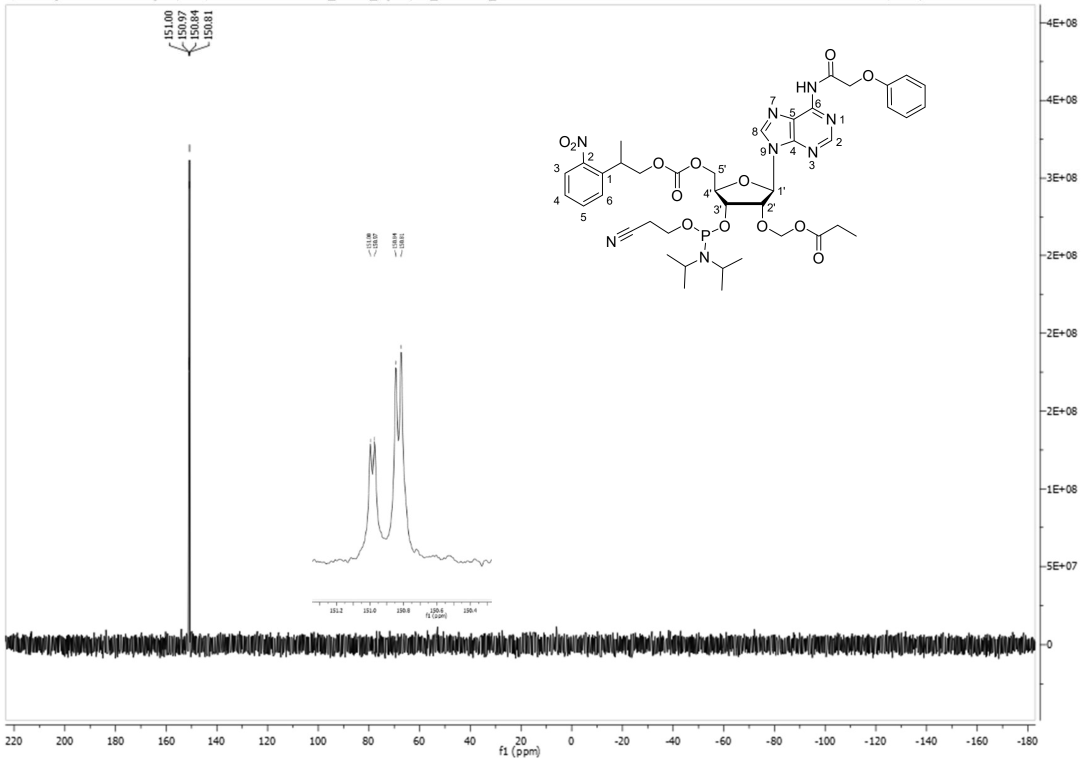

^{31}P-NMR spectrum of 5′-*O*-2-(2-nitrophenyl)propyloxycarbonyl-2′-*O*-propionyloxymethyl-3′-*O*-(2-cyanoethyl)-(*N,N*-di*iso*propyl)-phosphoramidite-N^2-*i*PrPac-guanosine (**5d**)

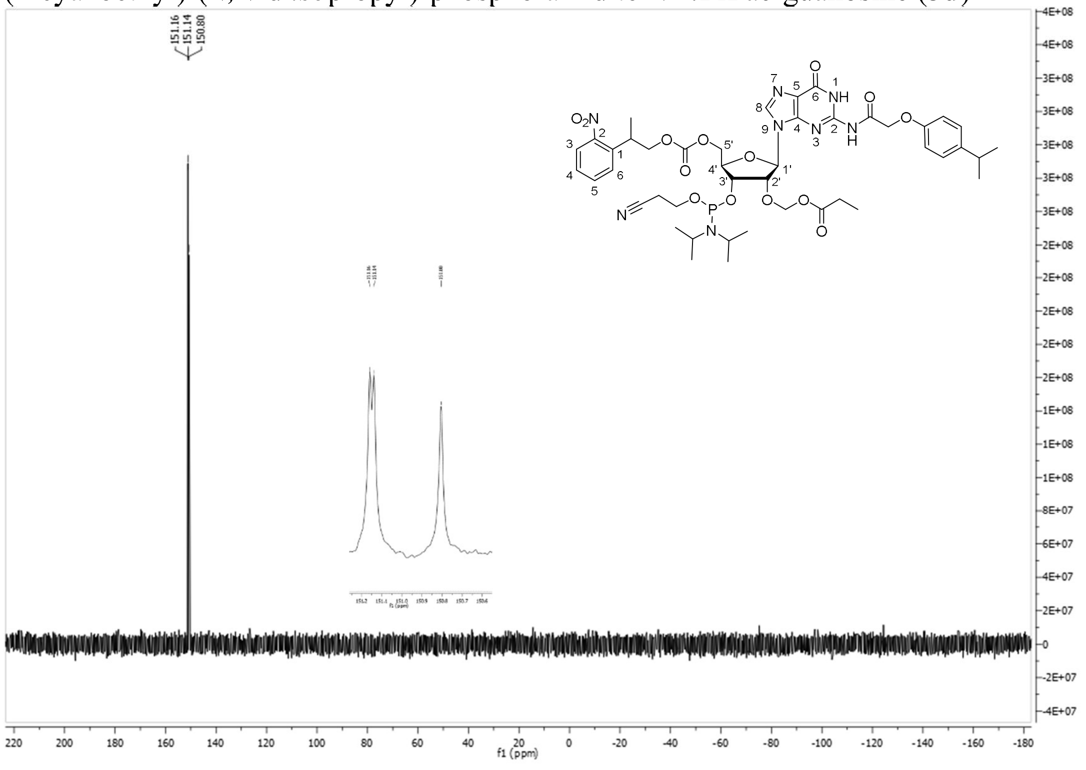

^{1}H-NMR spectrum of 5′-*O*-2-(2-nitro-4-ethyl-5-thiophenyl-phenyl)propyloxycarbonyl-3′-*O*-TBDMS-2′-*O*-propionyloxymethyl uridine (**6a**)

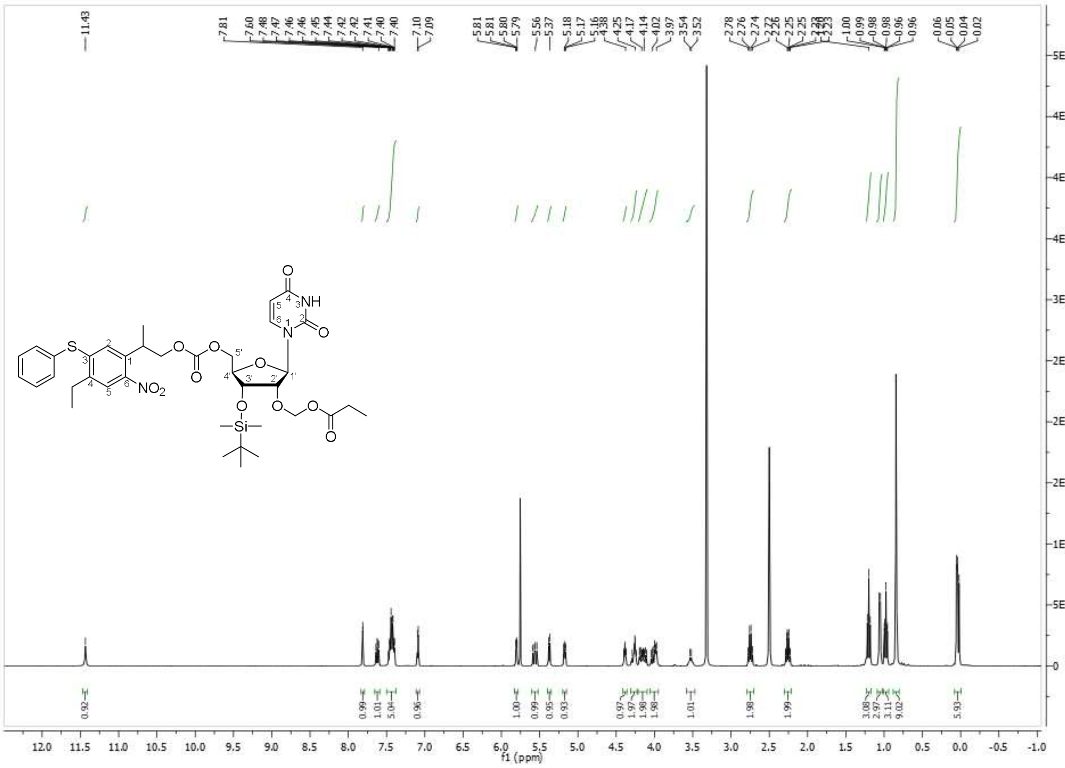

^{13}C-NMR spectrum of 5′-*O*-2-(2-nitro-4-ethyl-5-thiophenyl-phenyl)propyloxycarbonyl-3′-*O*-TBDMS-2′-*O*-propionyloxymethyl uridine (**6a**)

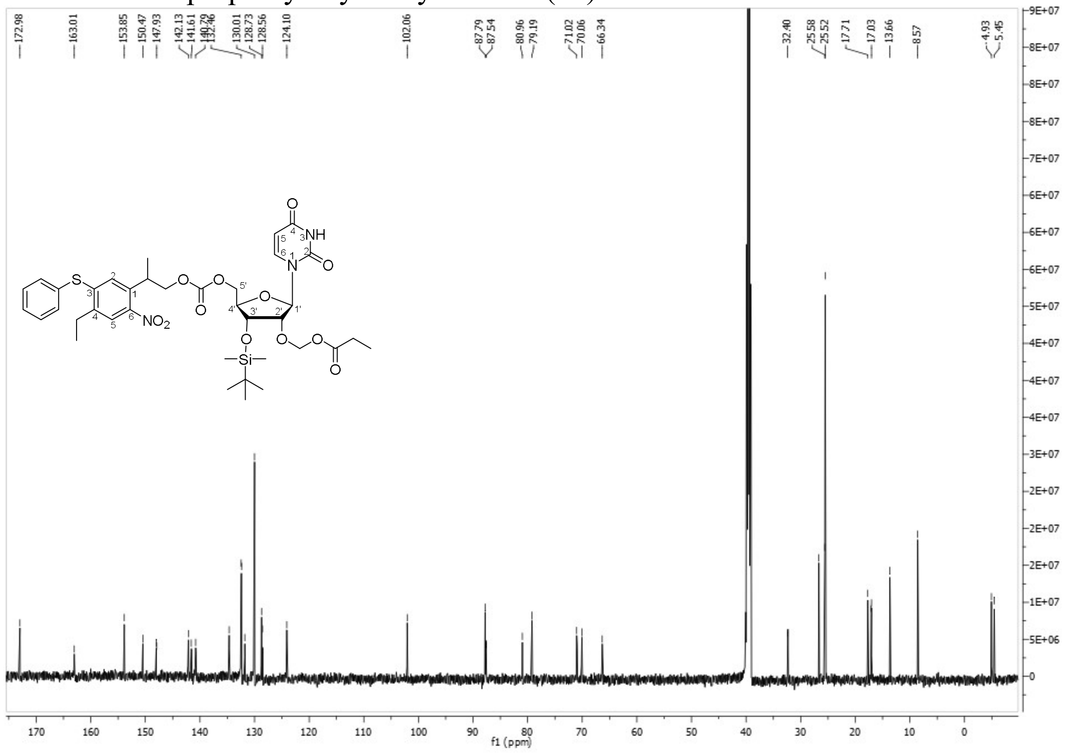

^{1}H-NMR spectrum of 5′-*O*-2-(2-nitro-4-ethyl-5-thiophenyl-phenyl)propyloxycarbonyl-3′-*O*-TBDMS-2′-*O*-propionyloxymethyl *N*^{4}-acetyl-cytidine (**6b**)

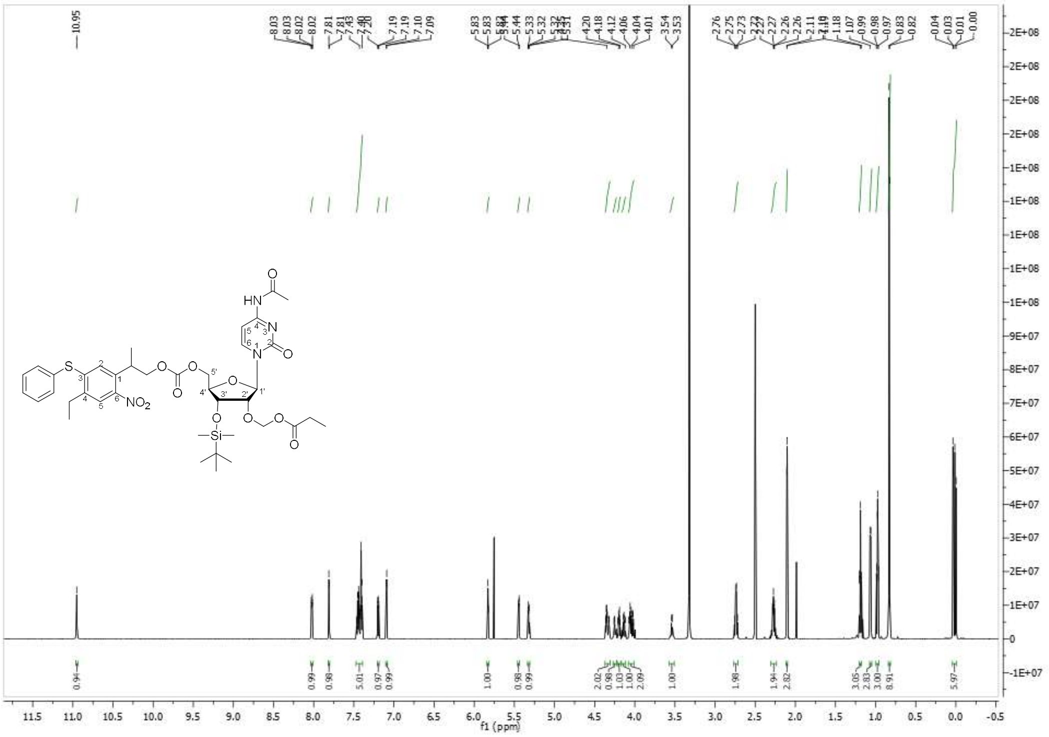

^{13}C-NMR spectrum of 5′-*O*-2-(2-nitro-4-ethyl-5-thiophenyl-phenyl)propyloxycarbonyl-3′-*O*-TBDMS-2′-*O*-propionyloxymethyl *N*^{4}-acetyl-cytidine (**6b**)

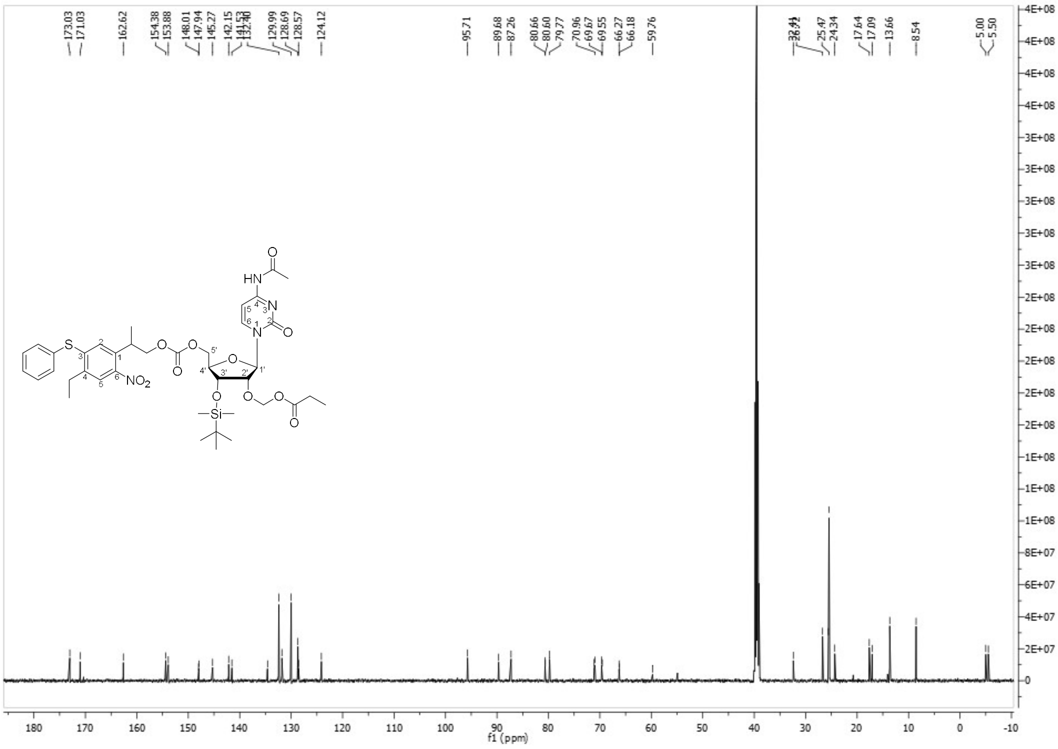

^{1}H-NMR spectrum of 5′-*O*-2-(2-nitro-4-ethyl-5-thiophenyl-phenyl)propyloxycarbonyl-3′-*O*-TBDMS-2′-*O*-propionyloxy-methyl-N^6-Pac-adenosine (**6c**)

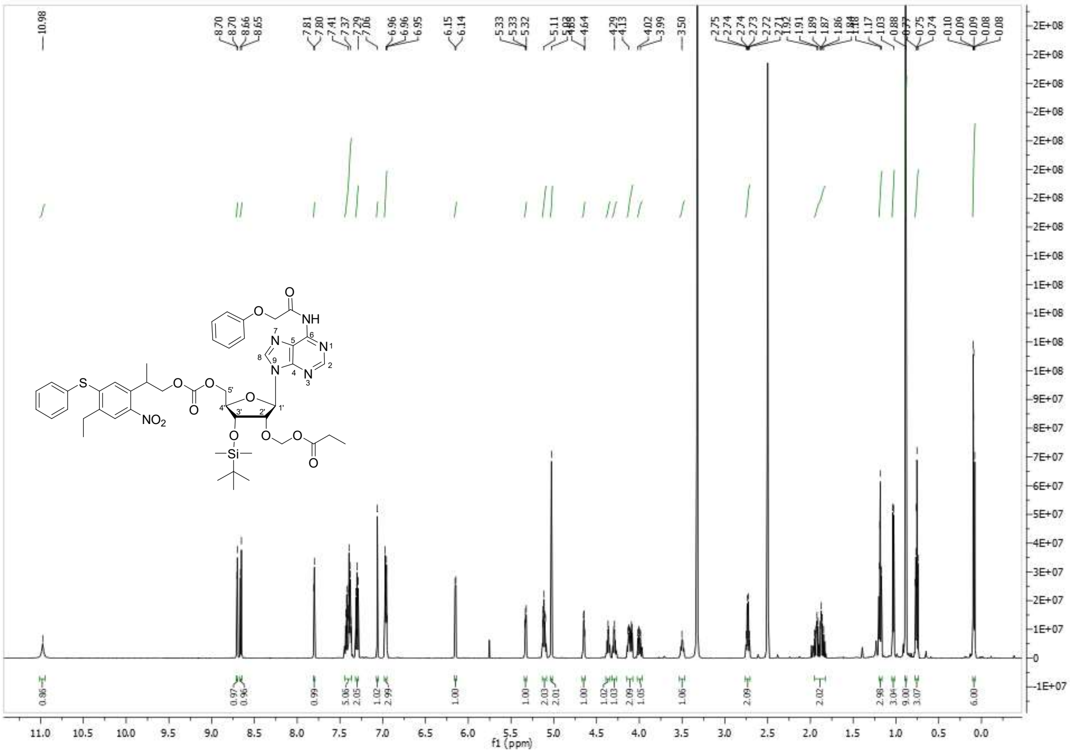

^{13}C-NMR spectrum of 5′-*O*-2-(2-nitro-4-ethyl-5-thiophenyl-phenyl)propyloxycarbonyl-3′-*O*-TBDMS-2′-*O*-propionyloxy-methyl-N^6-Pac-adenosine (**6c**)

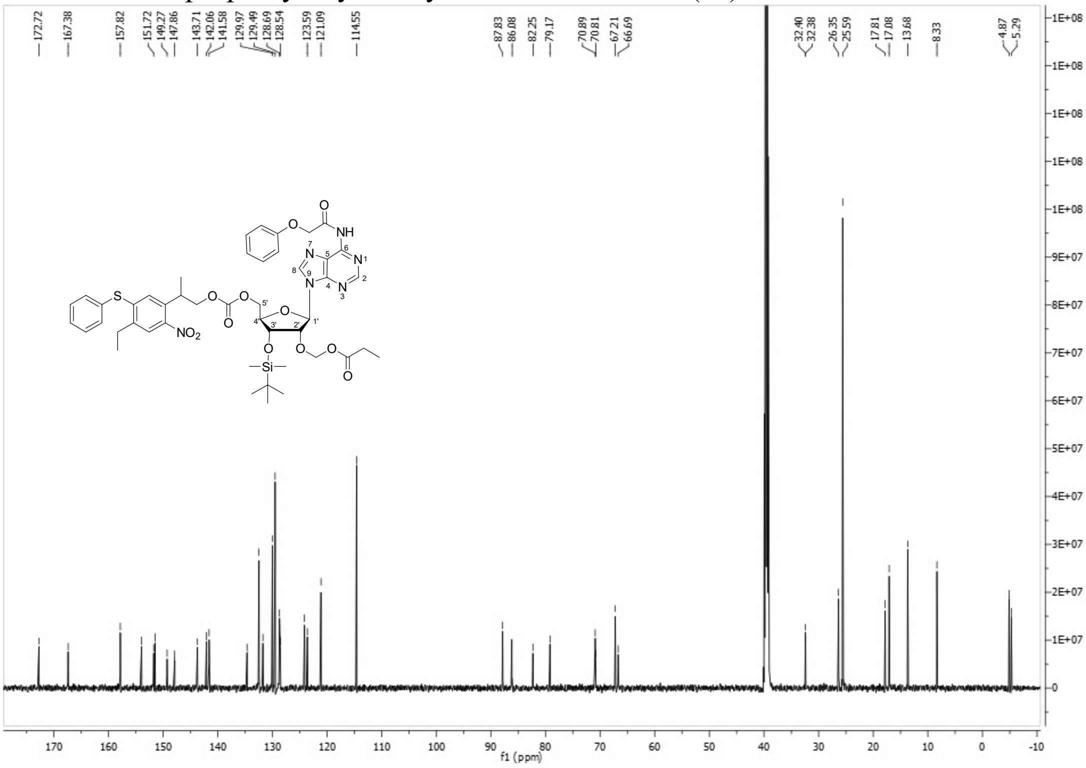

^{1}H-NMR spectrum of 5′-*O*-2-(2-nitro-4-ethyl-5-thiophenyl-phenyl)propyloxycarbonyl-3′-*O*-TBDMS-2′-*O*-propionyloxy-methyl-*N*^{2}-*i*PrPac-guanosine (**6d**)

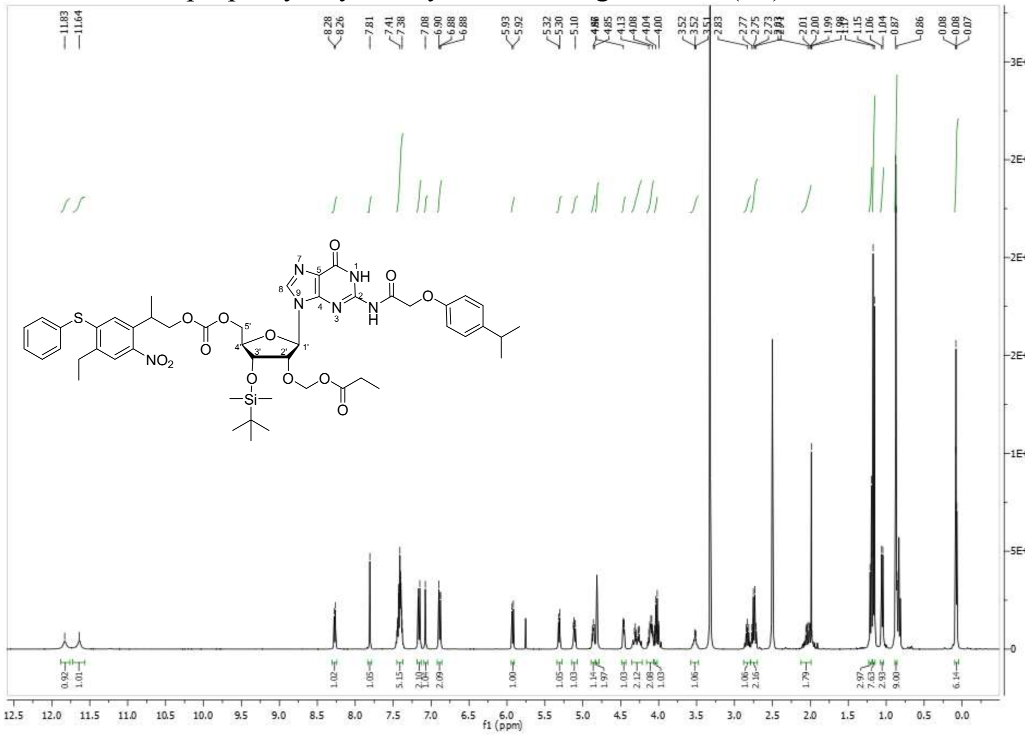

^{13}C-NMR spectrum of 5′-*O*-2-(2-nitro-4-ethyl-5-thiophenyl-phenyl)propyloxycarbonyl-3′-*O*-TBDMS-2′-*O*-propionyloxy-methyl-*N*^{2}-*i*PrPac-guanosine (**6d**)

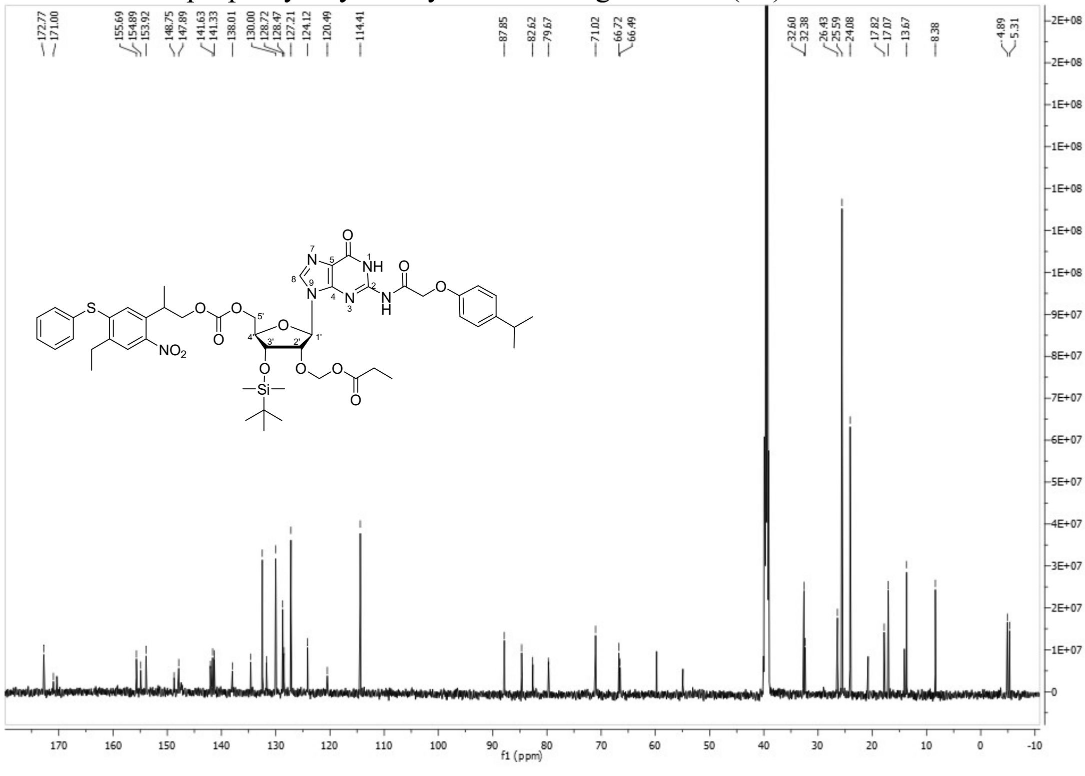

^{1}H-NMR spectrum of 5′-*O*-2-(2-nitro-4-ethyl-5-thiophenyl-phenyl)propyloxycarbonyl-2′-*O*-propionyloxymethyl uridine (**7a**)

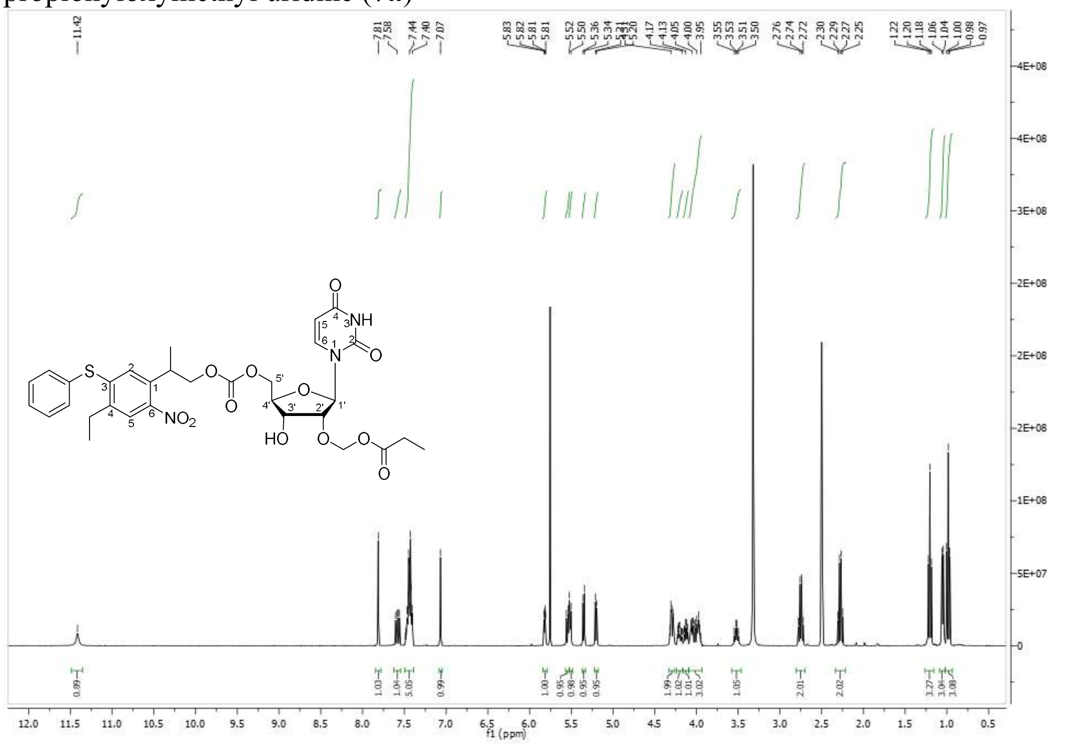

^{13}C-NMR spectrum of 5′-*O*-2-(2-nitro-4-ethyl-5-thiophenyl-phenyl)propyloxycarbonyl-2′-*O*-propionyloxymethyl uridine (**7a**)

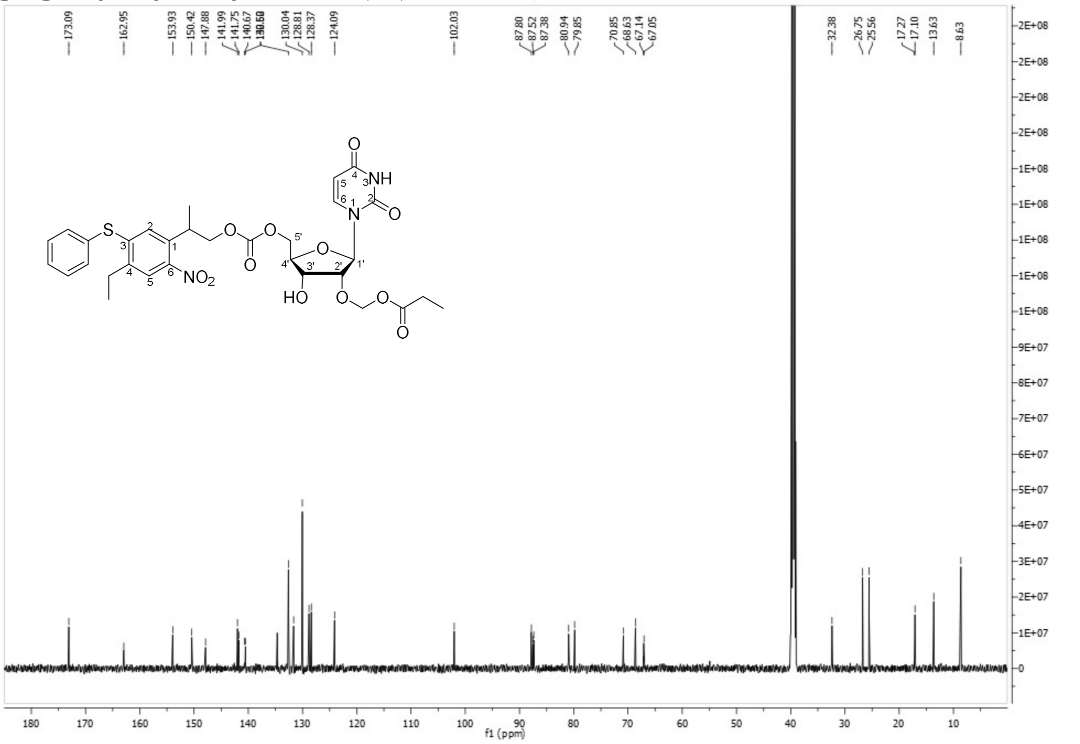

^{1}H-NMR spectrum of 5′-*O*-2-(2-nitro-4-ethyl-5-thiophenyl-phenyl)propyloxycarbonyl-2′-*O*-propionyloxymethyl *N*^{4}-acetyl-cytidine (**7b**)

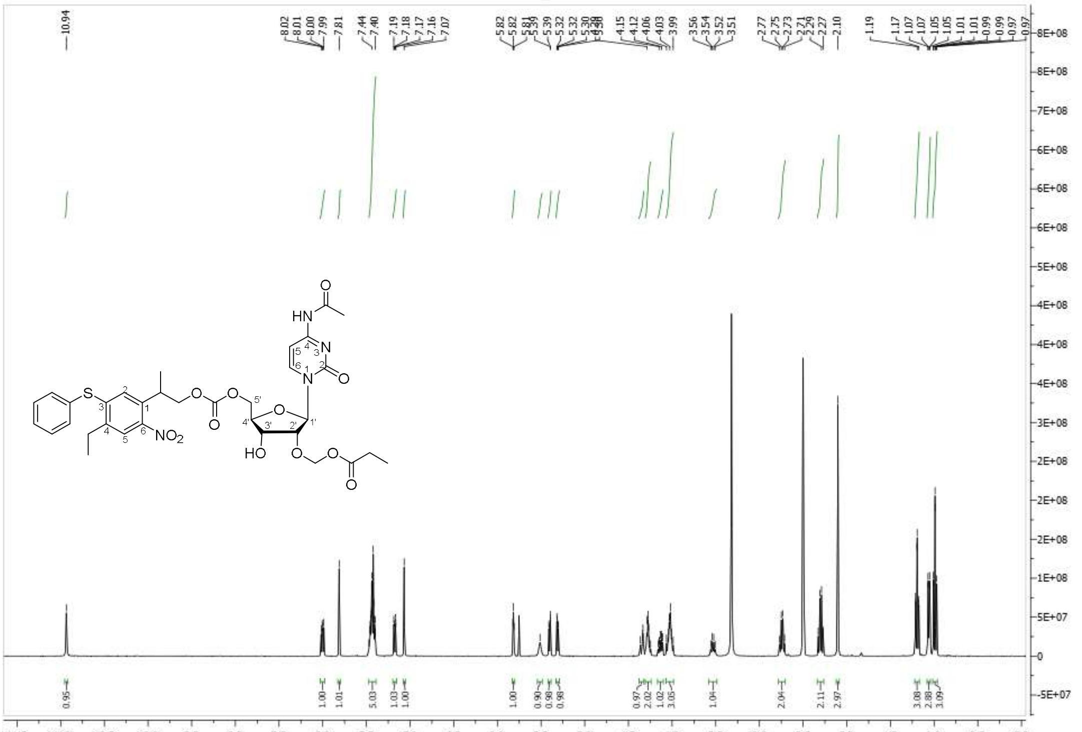

^{1}H-NMR spectrum of 5′-*O*-2-(2-nitro-4-ethyl-5-thiophenyl-phenyl)propyloxycarbonyl-2′-*O*-propionyloxymethyl *N*^{4}-acetyl-cytidine (**7b**)

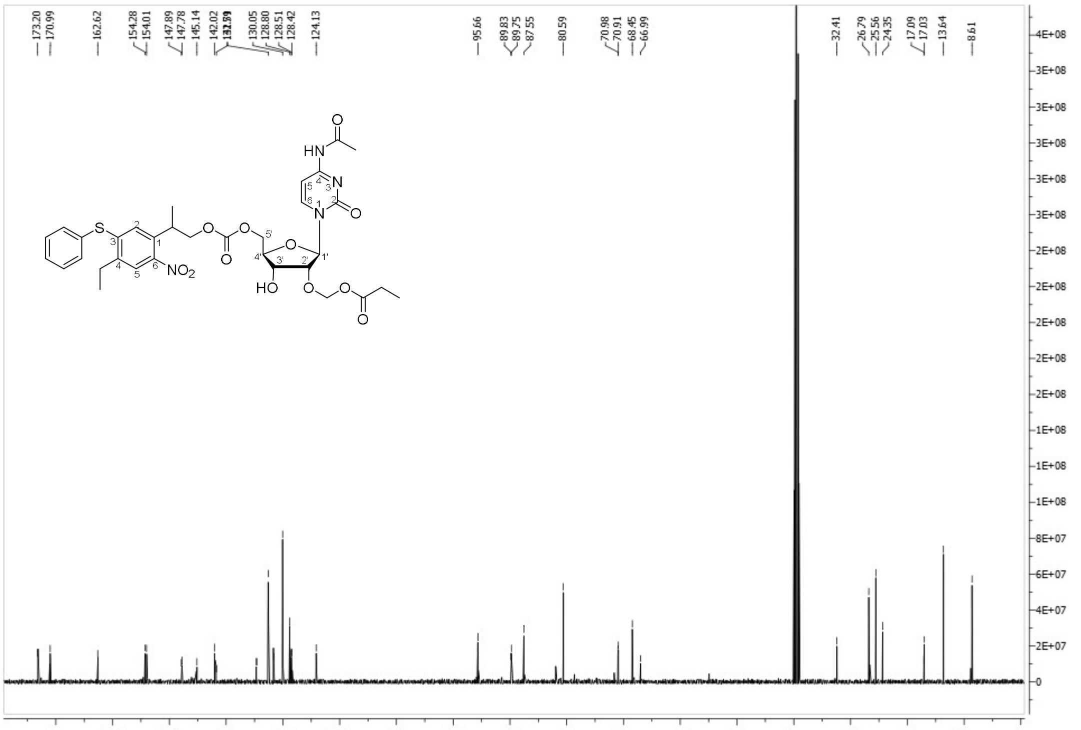

^{1}H-NMR spectrum of 5′-*O*-2-(2-nitro-4-ethyl-5-thiophenyl-phenyl)propyloxycarbonyl-2′-*O*-propionyloxymethyl-*N*[6]-Pac-adenosine (**7c**)

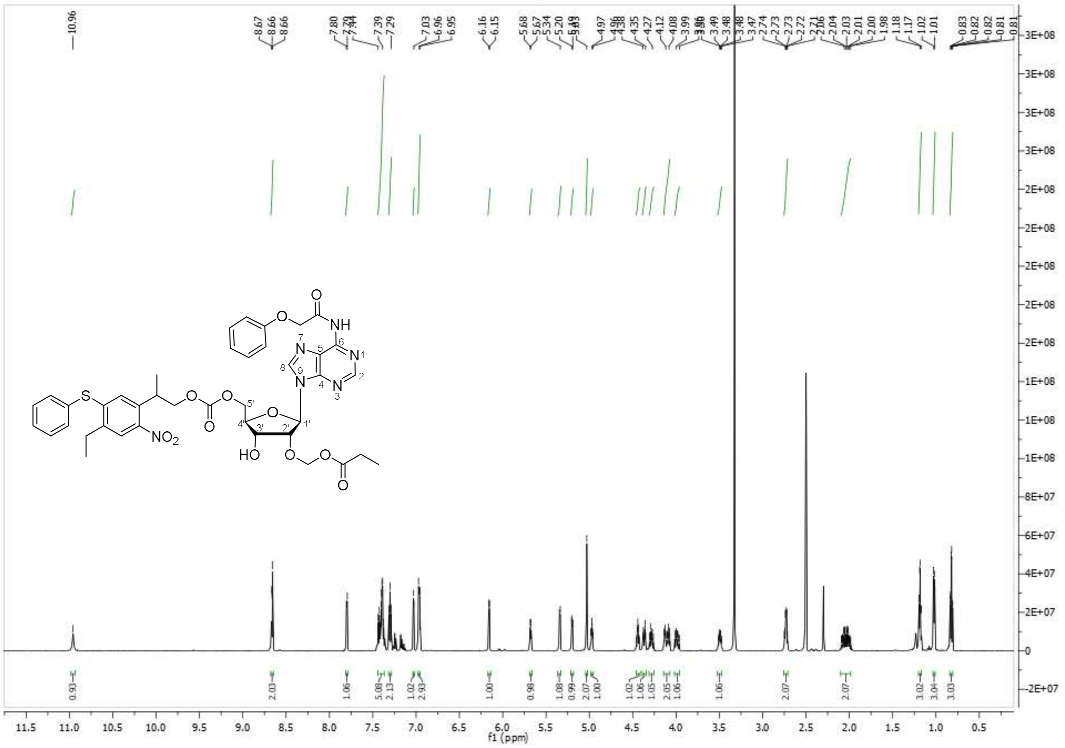

^{13}C-NMR spectrum of 5′-*O*-2-(2-nitro-4-ethyl-5-thiophenyl-phenyl)propyloxycarbonyl-2′-*O*-propionyloxymethyl-*N*[6]-Pac-adenosine (**7c**)

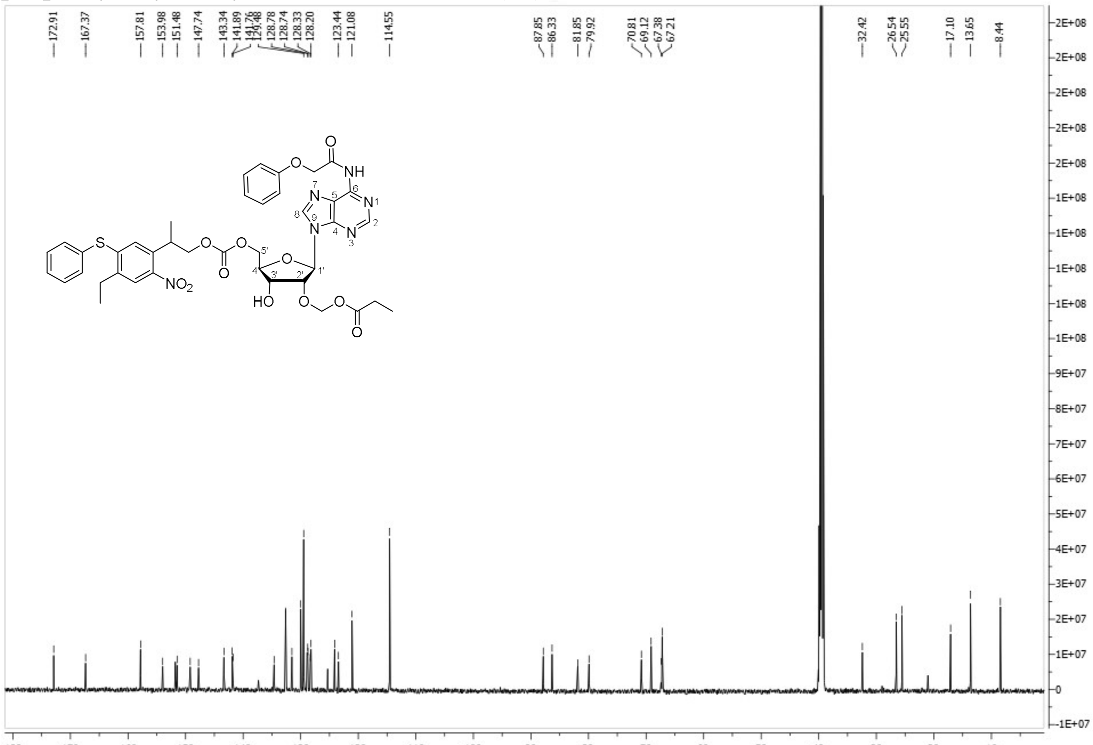

^{1}H-NMR spectrum of 5′-*O*-2-(2-nitro-4-ethyl-5-thiophenyl-phenyl)propyloxycarbonyl-2′-*O*-propionyloxymethyl-*N*^{2}- *i*PrPac-guanosine (**7d**)

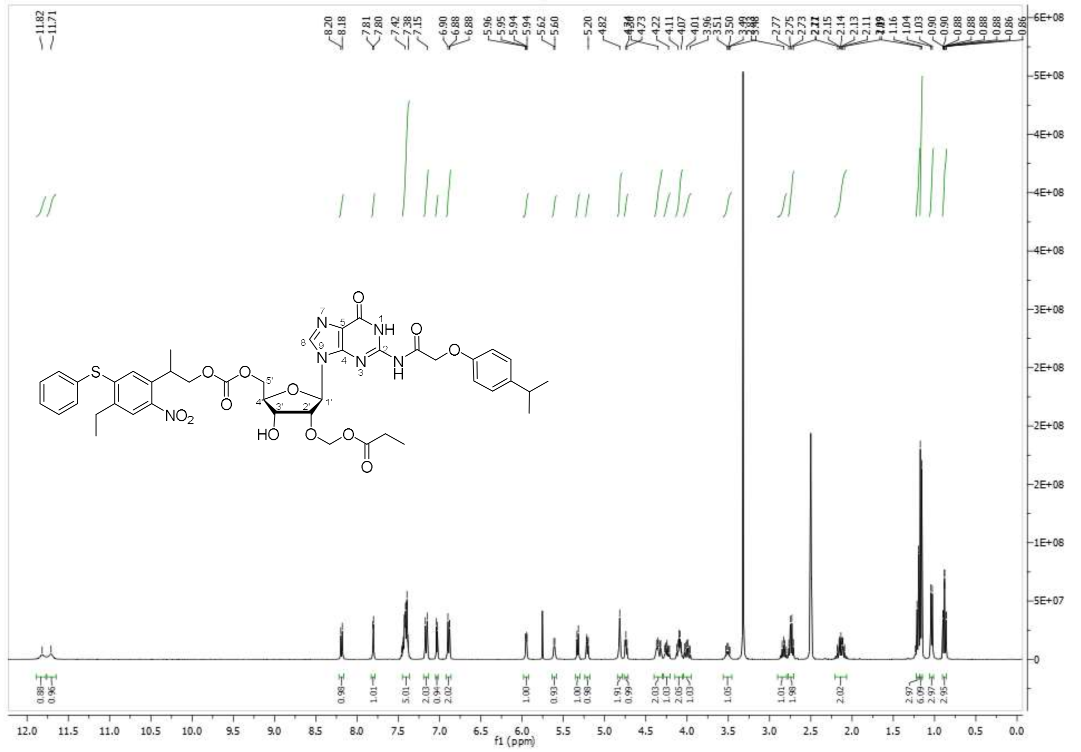

^{13}C-NMR spectrum of 5′-*O*-2-(2-nitro-4-ethyl-5-thiophenyl-phenyl)propyloxycarbonyl-2′-*O*-propionyloxymethyl-*N*^{2}- *i*PrPac-guanosine (**7d**)

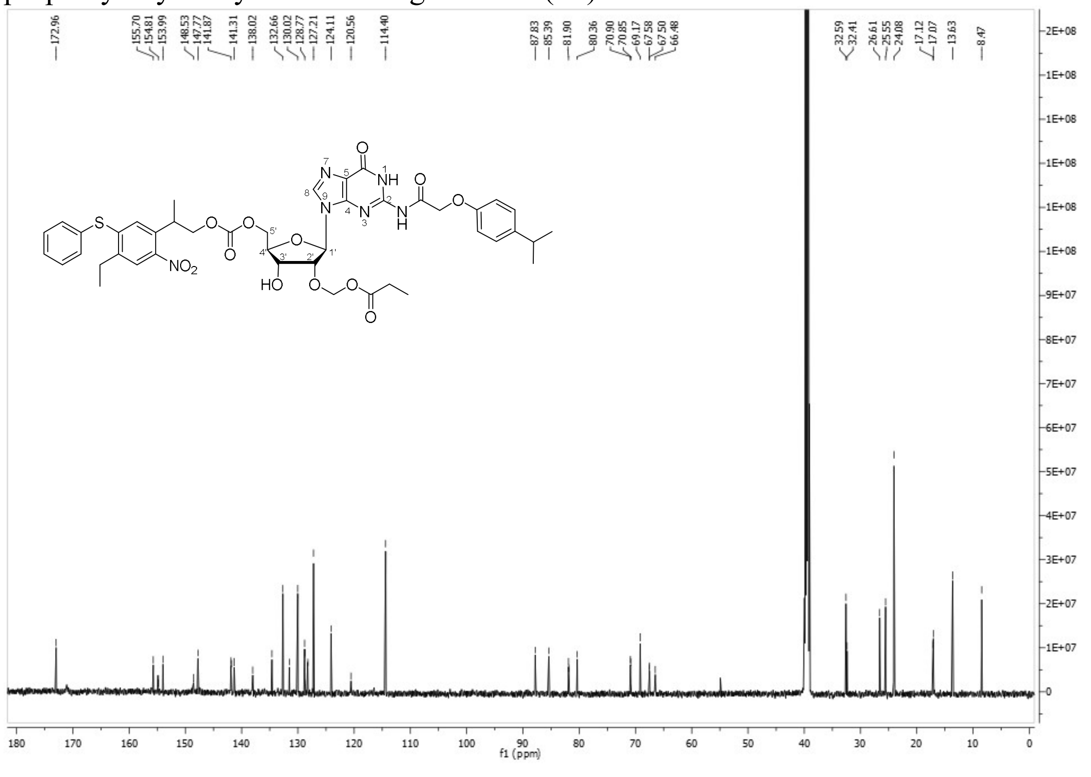

^{31}P-NMR spectrum of 5′-*O*-2-(2-nitro-4-ethyl-5-thiophenyl-phenyl)propyloxycarbonyl-2′-*O*-propionyloxymethyl-3′-*O*-(2-cyanoethyl)-(*N,N*-di*iso*propyl)-phosphoramidite uridine (**8a**)

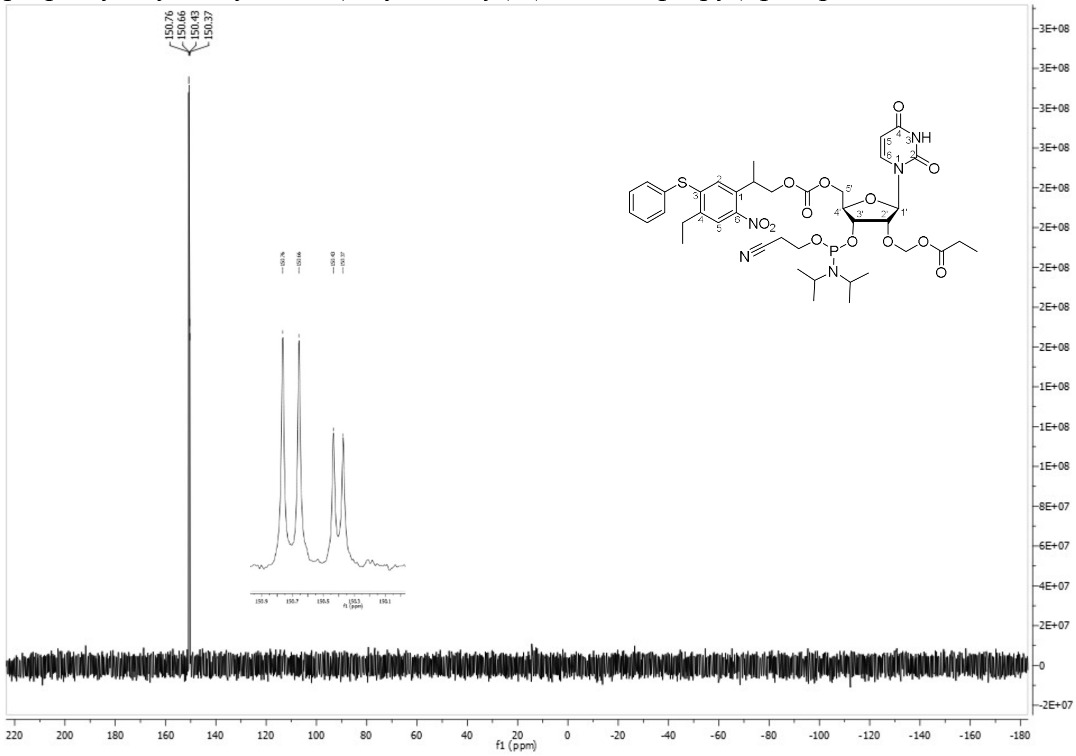

^{31}P-NMR spectrum of 5′-*O*-2-(2-nitro-4-ethyl-5-thiophenyl-phenyl)propyloxycarbonyl-2′-*O*-propionyloxymethyl-3′-*O*-(2-cyanoethyl)-(*N,N*-di*iso*propyl)-phosphoramidite N^4-acetyl-cytidine (**8b**)

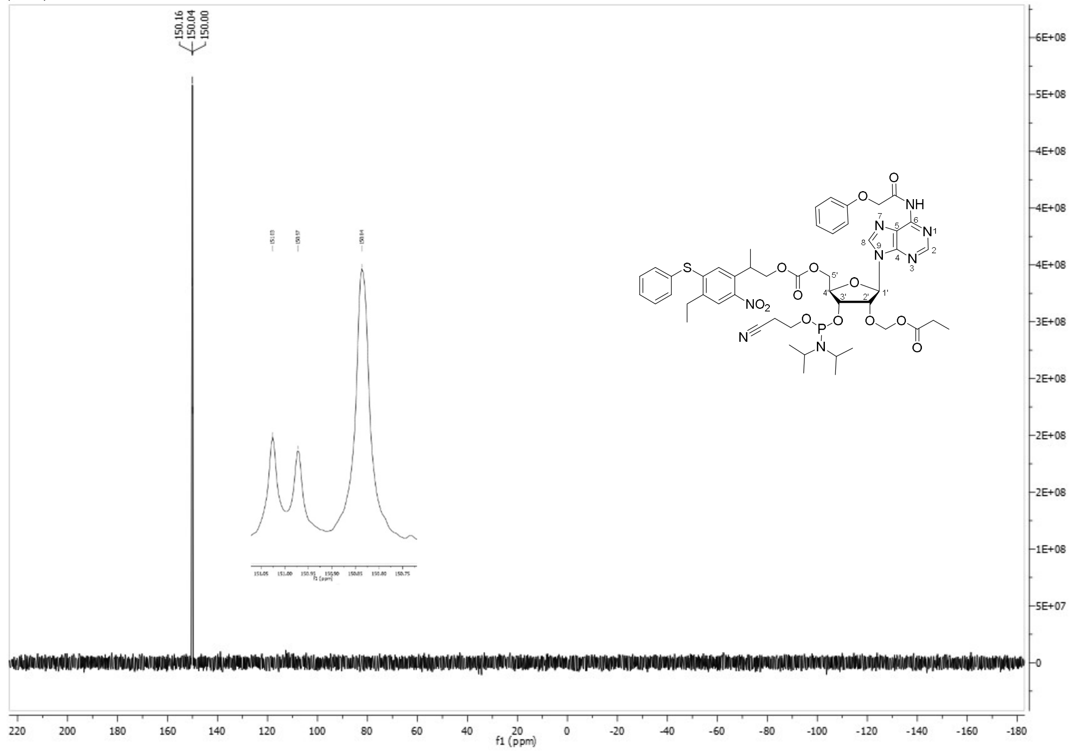

^{31}P-NMR spectrum of 5′-*O*-2-(2-nitro-4-ethyl-5-thiophenyl-phenyl)propyloxycarbonyl-2′-*O*-propionyloxymethyl-3′-*O*-(2-cyanoethyl)-(*N,N*-di*iso*propyl)-phosphoramidite *N*^{6}-Pac-adenosine (**8c**)

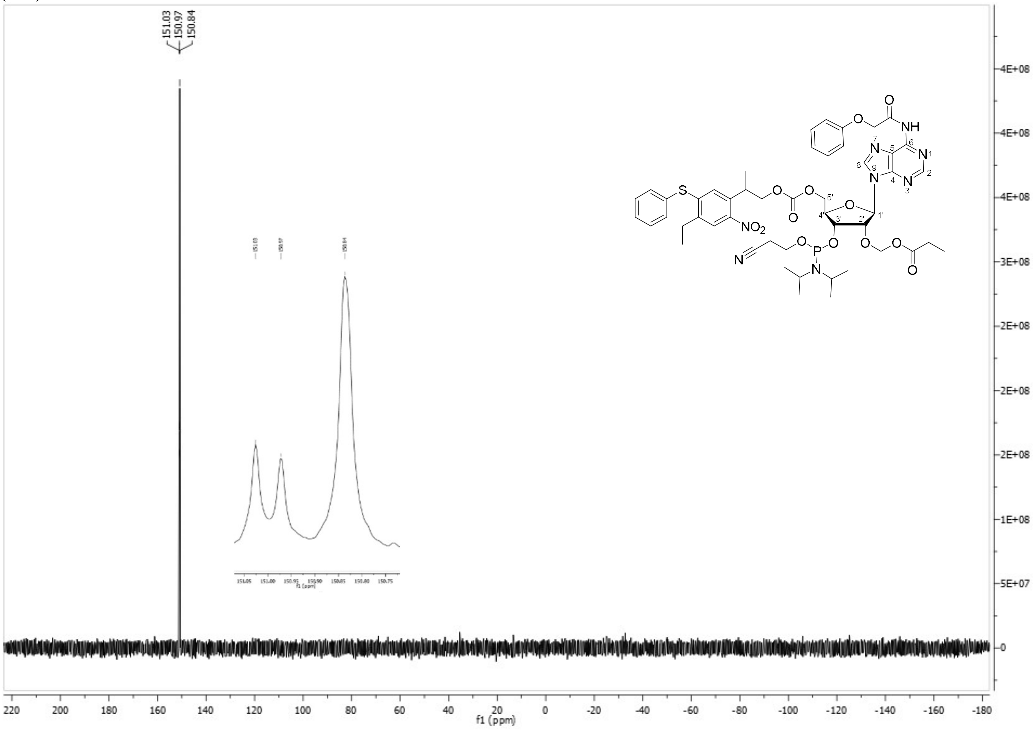

^{31}P-NMR spectrum of 5′-*O*-2-(2-nitro-4-ethyl-5-thiophenyl-phenyl)propyloxycarbonyl-2′-*O*-propionyloxymethyl-3′-*O*-(2-cyanoethyl)-(*N,N*-di*iso*propyl)-phosphoramidite *N*^{2}-*i*PrPac-guanosine (**8d**)

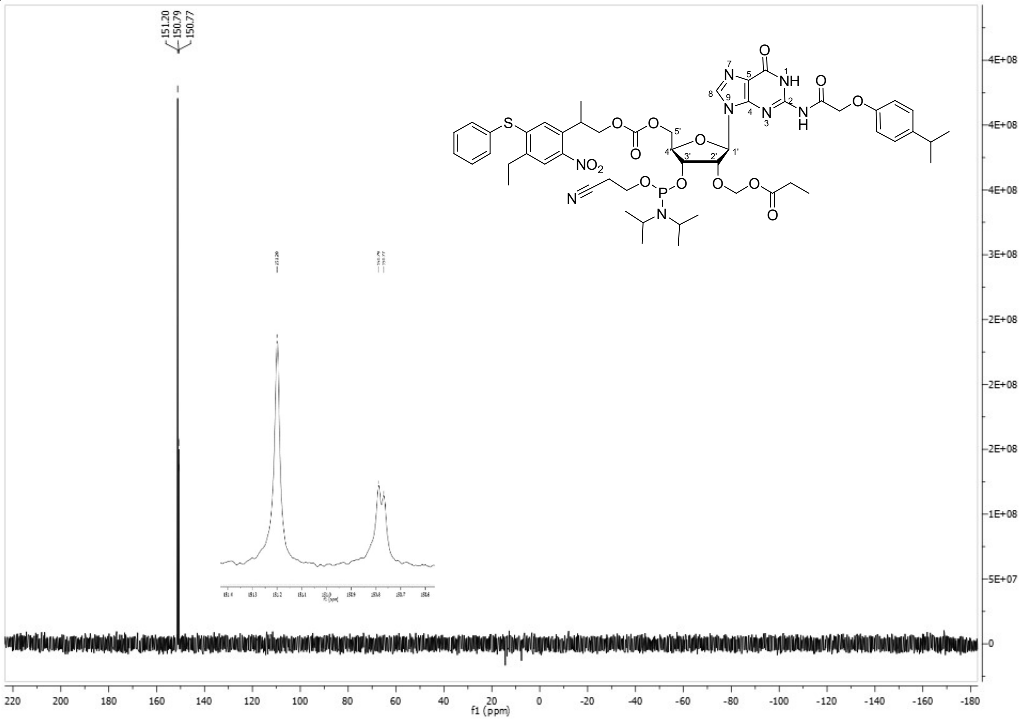

Materials and Methods for solid-phase oligonucleotide synthesis

Analytical high-performance anion-exchange chromatography was performed on a Thermoscientific Ultimate 3000 system equipped with a LPG3400RS pump or HPG3200BX pump, a DAD 3000 detector and a WPS-3000TBRS autosampler, column oven TCC-3000SD. IEX-HPLC was performed on DNAPac PA200 columns (4x250 mm, ThermoScientific). The following HPLC solvent systems were used: 5% CH_3CN in 25 mM Tris-HCl buffer pH 8 (eluent A) and 5% CH_3CN containing 400 mM of $NaClO_4$ in 25 mM Tris-HCl buffer pH 8 (eluent B). Flow rate was 1 mL/min for analytical. UV detection was performed at 260 nm. Chromeleon software was used. MALDI-TOF mass spectra were recorded on an AXIMA Assurance (Shimadzu Biotech, UK) equipped with 337 nm nitrogen laser, using a saturated solution of 2,4,6-trihydroxyacetophenone in a solution of acetonitrile/ammonium citrate (0.1 M) (1:1; v/v). The samples were mixed with the matrix in a 1:5 ratio (v/v) crystalized on 384-well stainless steel plate and analyzed.

Oligonucleotide synthesis was performed on LCAA-CPG solid support (Biosearch Technologies) at 1 μmole scale using an automated DNA synthesizer (Applied Biosystems 394) with TWIST synthesis columns (Glen Research) and oligonucleotide synthesis reagents from Biosearch Technologies. 2'-O-PrOM phosphoramidites were purchased from ChemGenes (MA, USA). The solid support was treated with a solution of Et_3N/CH_3CN (2:3) in anhydrous conditions for 1h30 min at room temperature. Then, the ORN was cleaved from solid support using a EDA/EtOH (1:1) solution for 1 h at room temperature. The solvents were evaporated under vacuum and coevaporated with toluene. The oligonucleotide was analyzed by IEX-HPLC and characterized by MALDI-TOF MS.
